# Trained Immunity Exacerbates Inflammatory Arthritis Progression via Promoting Synovial Fibroblast Ferroptotic Resistance

**DOI:** 10.1002/advs.202504245

**Published:** 2025-11-19

**Authors:** Haibo Su, Baoying Zhang, Qiudi Deng, Jiaxin Huang, Jinyu Feng, Yuan Fu, Yuejun Huang, Weikun Deng, YingJun Su, Huisheng Liu, Ning‐yi Shao, Zhenhui Zhang, Jianwei Dai

**Affiliations:** ^1^ Department of Critical Care Medicine GMU‐GIBH Joint School of Life Sciences The Guangdong‐Hong Kong‐Macao Joint Laboratory for Cell Fate Regulation and Diseases State Key Laboratory of Respiratory Disease Guangzhou Key Laboratory of Prevention and Treatment of Multiple Organ Dysfunction Syndrome the Second Affiliated Hospital Guangzhou Medical University Dongfengxi Road 195 Guangzhou Guangdong 510260 China; ^2^ Guangzhou National Laboratory Xingdao North Ring Road 9 Guangzhou Guangdong 510006 China; ^3^ School of Biomedical Engineering Guangzhou Medical University Dongfengxi Road 195 Guangzhou Guangdong 510180 China; ^4^ Department of Biomedical Sciences Faculty of Health Sciences University of Macau Taipa Macau Macau SAR 999078 China

**Keywords:** ferroptosis, inflammatory arthritis, innate immune memory, trained immunity, trained macrophages

## Abstract

Trained immunity induced by β‐glucan insult drives the functional reprogramming of macrophages to the hyperinflammatory status, contributing to developing or maintaining inflammatory diseases. Inflammatory arthritis is characterized by an idiopathically hyperinflammatory response, a phenotype similar to that of trained immunity, and its etiology involves environmental factors such as β‐glucan exposure. However, whether trained immunity contributes to inflammatory arthritis progression, as well as the reciprocal interactions, remains elusive. The study shows that β‐glucan‐induced experimental trained immunity heighten inflammation and arthritis severity in collagen‐induced arthritis (CIA) rat model. Trained macrophages by β‐glucan, upon adoptive transfer, further intensify symptoms. In arthritis progression, trained macrophages reduce fibroblast‐like synoviocytes’ (FLS) lipid peroxidation, lessening sensitivity to iFSP1‐induced ferroptosis through interleukin‐1 beta (IL‐1β)/N‐acetyltransferase 10 (NAT10)/ferroptosis suppressor protein 1 (FSP1) mRNA ac4C modification. A therapeutic approach targeting trained immunity, combining low‐dose iFSP1 and Remodelin, mitigates arthritis severity and restores ferroptosis sensitivity. Additionally, this interplay between CIA induction and β‐glucan training creates a feedback loop reinforcing trained immune memory, accelerating disease deterioration. The findings highlight trained immunity induced by endogenous or exogenous insult, such as β‐glucan, as an unexplored mechanism of inflammation dysregulation in the pathogenesis of inflammatory arthritis, opening avenues for the therapeutic approaches by targeting trained immunity.

## Graphical abstract

β‐glucan‐induced trained immunity reprograms macrophages toward hyperinflammation, exacerbating inflammatory arthritis. In a collagen‐induced arthritis model, trained macrophages intensify disease severity and confer ferroptosis resistance in synovial cells via IL‐1β/NAT10‐mediated FSP1 mRNA modification. Targeting this pathway with combined iFSP1 and Remodelin therapy alleviates arthritis, revealing trained immunity as a novel therapeutic target for autoimmune disorders.

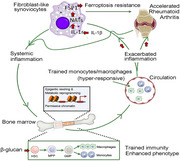

## 1 Introduction

Trained immunity has been defined as the long‐term functional reprogramming of macrophages, induced by *Candida albicans*, β‐glucan*, Bacillus Calmette‐Guérin* (BCG), or other endogenous/exogenous insults, macrophages then revert to a quiescent state but display modified inflammatory responses upon subsequent challenges.^[^ ^1^, ^2^ ^]^ This evolutionary adaptation is thought to enhance resistance to reinfection; however, it may also be detrimental and contribute to pathologies of inflammatory diseases.^[^ ^3^, ^4^, ^5^, ^6^ ^]^ Trained macrophages exacerbated the fibro‐inflammatory phenotype of endometriotic cells, thereby promoting lesion growth in a murine model of endometriosis.^[^ ^7^ ^]^ Macrophage training is associated with accelerated inflammation and fibrosis in systemic sclerosis in mice.^[^ ^8^ ^]^ Trained immunity is implicated in acute rejection episodes.^[^ ^9^ ^]^ Mutations in macrophages have been demonstrated to induce trained immunity, leading to tissue damage associated with neoplasms.^[^ ^10^ ^]^ β‐glucan‐induced training immunity accelerates the development of systemic lupus erythematosus‐like syndrome in mice.^[^ ^1^ ^]^ Although persistent hyperactivation of the immune response owing to trained immunity is hypothesized to contribute to various inflammatory diseases, the precise mechanisms and extent of its involvement remain unclear.^[^ ^11^, ^12^, ^13^ ^]^

Inflammatory arthritis, including disorders of incompletely understood etiology, such as rheumatoid arthritis (RA), is characterized by persistent synovial inflammation.^[^ ^14^ ^]^ The trained immunity phenotype is observed in macrophages from patients with inflammatory arthritis^[^ ^15^ ^]^: for example, macrophages from patients with RA exhibit features consistent with a trained immunity phenotype. Functionally, these macrophages demonstrate enhanced responsiveness to stimuli such as LPS, leading to increased production of pro‐inflammatory cytokines.^[^ ^16^ ^]^ Metabolically, they display increased glucose uptake, elevated oxygen consumption, and accumulation of metabolites (including glutamate and fumarate), indicating a hypermetabolic state.^[^ ^16^, ^17^, ^18^ ^]^ Hypomethylation of the TNF‐α promoter has been observed.^[^ ^19^ ^]^

β‐glucan is a classic stimulant that induces trained immunity,^[^ ^1^, ^2^ ^]^ and is suspected to be associated with the pathological development of inflammatory arthritis, including RA.^[^ ^20^ ^]^ SKG mice, an autoimmune arthritis model, exhibit few symptoms in a clean environment. However, a single intraperitoneal injection of zymosan (a fungal β‐glucan) or purified Curdlan and laminin (a bacterial β‐glucan) can trigger severe chronic arthritis in SKG mice in this sterile environment, but only cause transient inflammation in normal mice. Blocking Dectin‐1, a major β‐glucan receptor, can prevent SKG arthritis triggered by β‐glucan.^[^ ^21^ ^]^ β‐glucan from *Candida albicans* can accelerate the progression of collagen‐induced arthritis (CIA)‐induced arthritis in mice.^[^ ^22^ ^]^ Fungal β‐glucan purified from zymosan can accelerate the deterioration of CIA symptoms.^[^ ^23^ ^]^ However, whether and how trained immunity drives pathological inflammation in RA remains unresolved: endogenous or exogenous insults, including β‐glucan exposure stimuli, reprogram macrophages into a trained phenotype that contributes to inflammatory arthritis progression.^[^ ^11^, ^24^, ^25^ ^]^

Ferroptosis is a key mode of cell death during the progression of inflammatory arthritis, including RA.^[^ ^26^ ^]^ The ferroptosis resistance of Fibroblast‐like synoviocytes (FLS) derived from patients with RA is a characteristic of disease progression, as reflected by decreased levels of ACSL4 but increased levels of GPX4 and SLC7A11.^[^ ^27^ ^]^ Single‐cell RNA sequencing has also identified a ferroptosis‐resistant fibroblast subset in the synovium of patients with RA and CIA mice.^[^ ^28^ ^]^ This observation requires further validation, and the underlying mechanism of ferroptosis resistance in FLS remains largely unknown. Inflammatory cytokines may be a potential mechanism of ferroptotic resistance via the regulation of lipid peroxidation and ferroptosis.^[^ ^29^, ^30^ ^]^ Increased production of IL‐1β, TNF, IL‐6, and TGF‐β was detected in the supernatant of trained macrophages co‐cultured with fibroblasts, implying potential crosstalk between trained innate immune cells and fibroblasts that may act as a second stimulus to enhance inflammatory cytokine production.^[^ ^8^ ^]^ However, a causal link between trained immunity and ferroptosis resistance in FLS under conditions of inflammatory arthritis has not been reported. Thus, we hypothesized that trained macrophages, as they circulate throughout the body to reach the joints, might enhance inflammatory cytokine production through potential interactions with FLS, thereby endowing FLS with ferroptosis resistance.

In this study, we investigated the impact of trained immunity on the outcome of inflammatory arthritis, as modeled via experimental trained immunity induced by β‐glucan insults and CIA in rats. Thus, we demonstrate that both in vivo training and the adoptive transfer with trained macrophages exacerbated inflammation and disease severity. Furthermore, we found that these effects pertain to IL‐1β signaling resulting from the crosstalk between β‐glucan‐trained macrophages and FLS from CIA rats, thereby contributing to the ferroptosis‐resistant phenotype in FLS. Our findings not only shed new light on the dynamic role of trained immunity by which environmental factors, such as β‐glucan exposure, drive the pathogenesis of arthritis via promoting ferroptosis resistance of synovial FLS, but also pave the way for original therapeutic strategies based on trained macrophages to counteract ferroptosis and autoimmune arthritis.

## 2 Results

### 2.1 Trained Immunity Affects Inflammatory Arthritis Development in an Established CIA Rat Model

The proposed in vitro training scheme for trained immunity induced by β‐glucan from *C. albicans* was adapted to rat bone marrow‐derived macrophages (BMDMs) (Figure S1A, Supporting Information). Results showed that β‐glucan‐trained macrophages increased cytokine production, glycolysis, and epigenetic changes (Figure S1B–D, Supporting Information) upon LPS re‐stimulation compared to untrained macrophages, indicating trained immunity responses. In vivo, enhanced protective effects were found in β‐glucan‐trained rats to lethal candidiasis (Figure S1E,F, Supporting Information).

**Figure 1:**
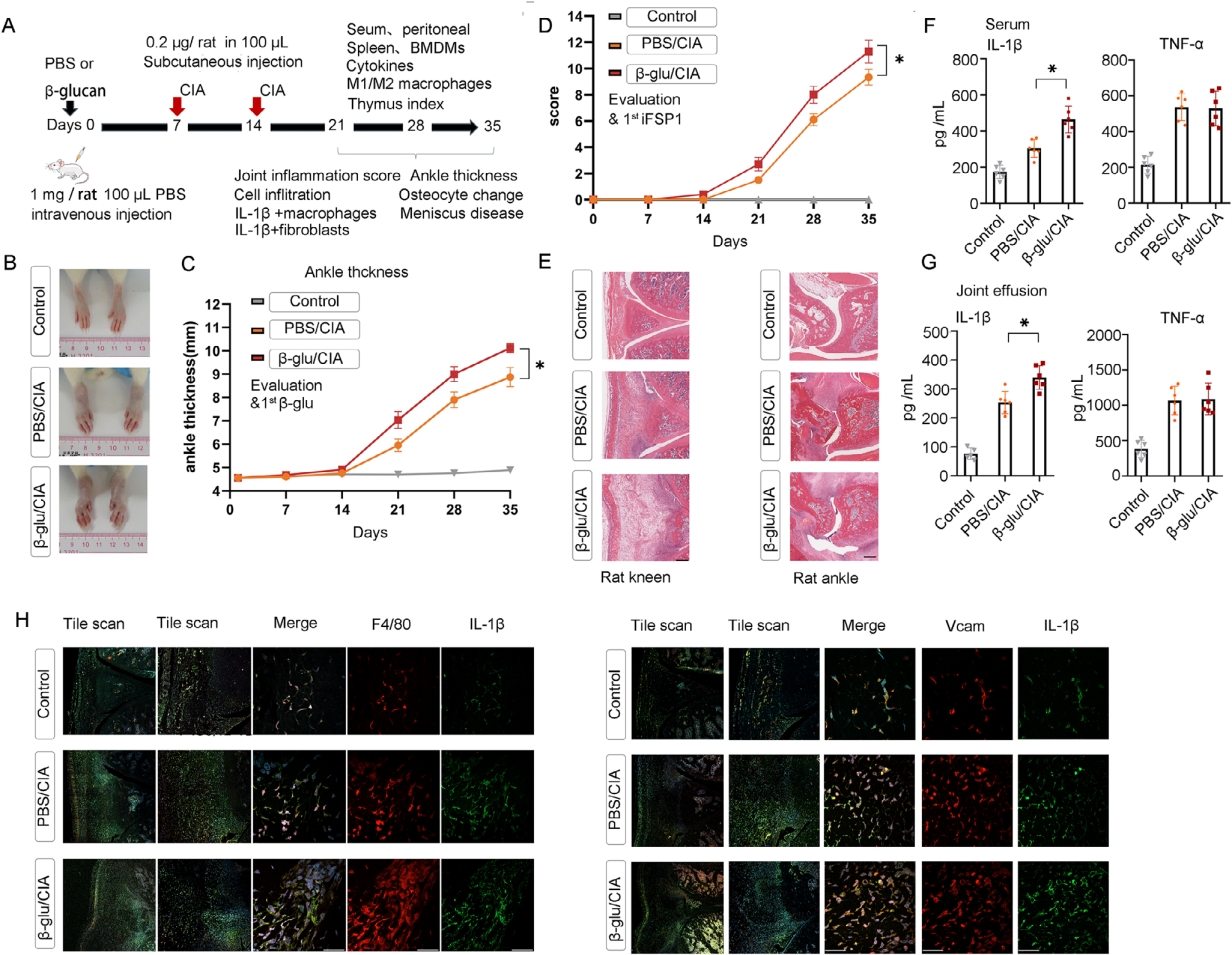
Trained immunity affects inflammatory arthritis development in an established CIA rat model. A) Schematic experimental induction of trained immunity in the CIA rat model, B) Representative images of the hind paw of rats with or without β‐glucan training on day 35 (*n* = 1), C) Hind paw thickness was calibrated from the second immunisation onwards (*n* = 6), D) Arthritis scores were monitored according to A (*n* = 6), E) Hematoxylin and eosin (H&E) images of representative knee and ankle in rats at day 35, scale bars, 200 µm., F,G) Levels of IL‐β and TNF‐a in the serum (G) or joint effusion (H) of rats (*n* = 6)., H) Representative immunofluorescence assays and the proportion of IL‐β^+^VCAM^+^ or IL‐β^+^/F4/80^+^ cells in the joints of rats, representative sections per rat, scale bars, 50 µm., In C, D, F, G, data are presented as mean ± SD, C & D,^*^ *p* <0.05 by a Two‐Way ANOVA; F, G, ^*^ *p* <0.05 by a One‐way ANOVA. See also in Figure S2 (Supporting Information).

To evaluate whether trained immunity induced by environmental factors such as anterior antigenic β‐glucan exposure contributes to inflammatory arthritis severity, the rats were euthanized to analyze the pathological feature changes of inflammatory arthritis on day 35 of the experiment (**Figure** **1**A). We found that β‐glucan/CIA rats exhibited greater severity than PBS/CIA rats or the control group, characterized by joint swelling (Figure 1B–D; Figure S2A, Supporting Information). Histological analysis revealed more severe synovial inflammation and bone destruction in β‐glucan/CIA rats (Figure 1E; Figure S2B,C, Supporting Information). IL‐1β has been implicated in the pathogenesis of inflammatory arthritis.^[^ ^31^ ^]^ We observed that β‐glucan/CIA rats showed an increase in IL‐1β level in serum and joint effusion, compared to PBS/CIA group (Figure 1F,G). Additionally, trained immunity strikingly increased the population of IL‐1β^+^/VCAM^+^ FLS and synovial IL‐1β^+^/F4/80^+^ macrophages (Figure 1H). These data suggest that experimental trained immunity by anterior antigenic β‐glucan insults worsens the inflammatory arthritis progression.

### 2.2 Adoptive Transfer of Trained Macrophages Exacerbates Inflammatory Arthritis Progression Associated with Phenotypic Program of Fibroblast‐Like Synoviocytes

**Figure 2:**
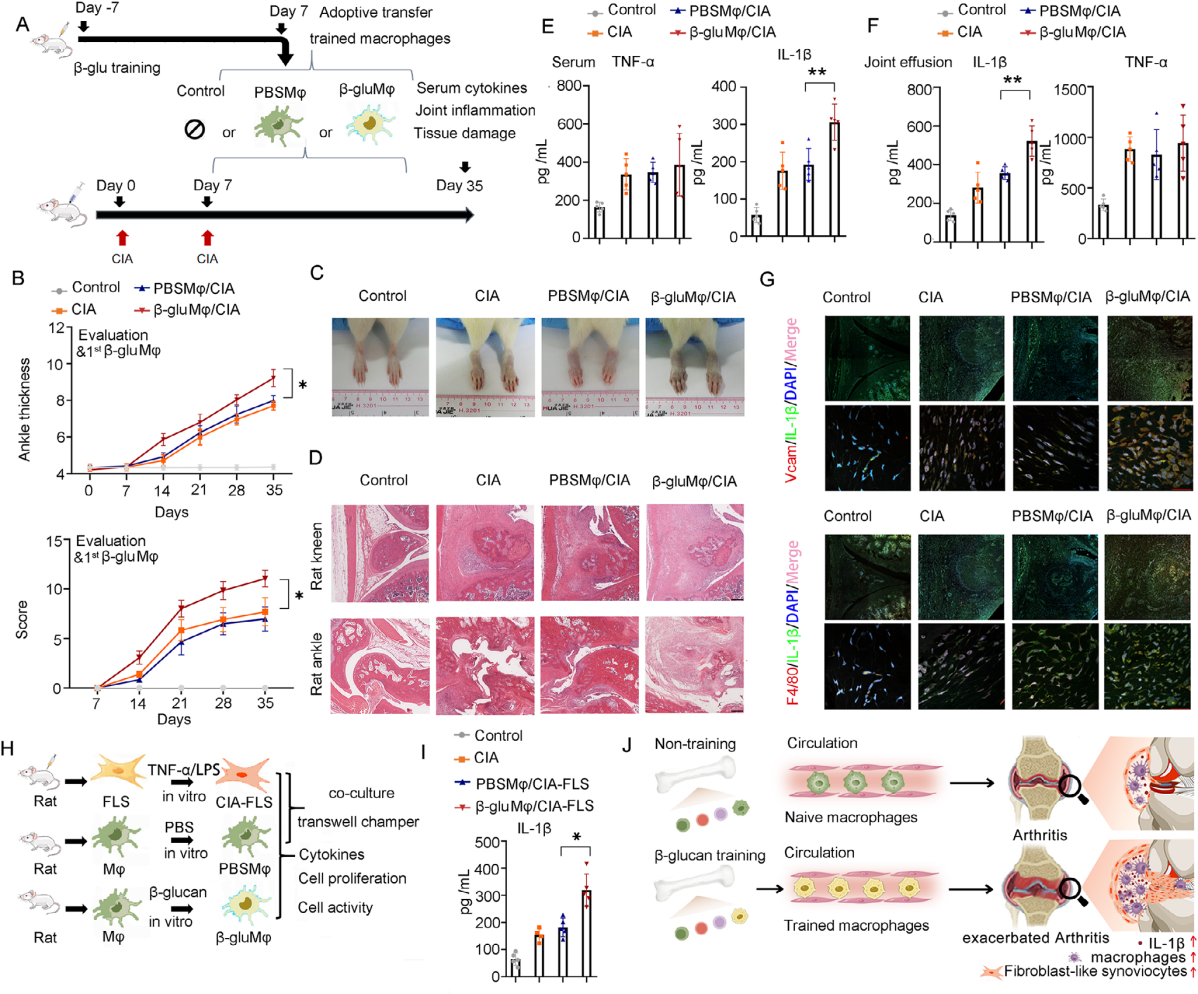
Adoptive transfer of trained macrophages aggravates inflammatory arthritis progression associated with phenotypic program of fibroblast‐like synoviocytes, A) Experimental setup for adoptive transfer of trained macrophages into CIA rat via intraperitoneal injection, B) Arthritis score was monitored, and the hind paw was calibrated from the second immunisation onwards (*n* = 5), C) Images of the hind paw of rats (*n* = 1), D) Hematoxylin and eosin images of representative knee and ankle from rats, scale bars, 200 µm, E,F) The levels of IL‐β and TNF‐a in the serum (E) or joint effusion (F) of rats (*n* = 5), G) Immunofluorescence assays of IL‐β^+^VCAM^+^ or IL‐β^+^/F4/80^+^ cells in the joint of rats, scale bars, 50 µm, H) Experimental setup of the co‐culture model in a transwell chamber using PBS‐Mφ or β‐glu‐Mφ and rat CIA‐FLS. Macrophages and CIA‐FLS were seeded at a ratio of 1:1 (≈1.0 × 10^5^ cells), and were separated using a co‐culture chamber for 72 h. Macrophages were placed in the upper chamber, and CIA‐FLS were located in the lower chamber, I) IL‐β production in the supernatant of CIA‐FLS after 72 h co‐culture incubation (*n *= 4 or 5), J) Training immunity to exacerbate rheumatoid arthritis schematic, In B, E, F, I, data are presented as mean ± SD, E, F, I,^*^ *p* <0.05, ^**^ *p* <0.01 by a One‐way ANOVA; B, ^*^ *p* <0.05 by a Two‐Way ANOVA. See also in Figures S3 and S4 (Supporting Information).

To determine the role of macrophage training in the progression of inflammatory arthritis, we treated CIA rats with one injection of rat β‐glucgan‐trained BMDMs (β‐glu‐Mφ) (**Figure** **2**A). Peritoneal injection of β‐glu‐Mφ accelerated the severity by increasing the arthritis score and the ankle circumference in CIA rats, compared to treatment with PBS‐trained macrophages (PBS‐Mφ) (Figure 2B,C; Figure S3A, Supporting Information). β‐glu‐Mφ/CIA rats showed increased hyperplasia and cartilage and bone destruction in the joint (Figure 2D; Figure S3B,C, Supporting Information). IL‐1β level increased in the serum and joint effusion of β‐glu Mφ/CIA rats compared to the PBS‐Mφ/CIA group (Figure 2E,F). In addition, the number of IL‐1β^+^/VCAM^+^ FLS was higher in β‐glu‐Mφ/CIA rats than that in the PBS‐Mφ/CIA group (Figure 2G; S3D, Supporting Information).

In vitro, rat β‐glu‐Mφ and rat CIA‐FLS were further co‐cultured in a transwell system (Figure 2H; Figure S4A, Supporting Information). Compared with CIA‐FLS or PBS‐Mφ/CIA‐FLS, the level of IL‐1β was increased in the supernatant of β‐glu‐Mφ/CIA‐FLS (Figure 2I; Figure S4B, Supporting Information). Human β‐glucgan‐trained Thp‐1 (β‐glu‐hMφ) had a similar inflammatory effect on RA‐hFLS (Figure S4C,D, Supporting Information). Thus, macrophage training aggravates the severity of arthritis, which may be partly associated with the enhancement of IL‐1β signaling and the phenotypic remodeling of FLS in the joint (Figure 2J).

### 2.3 Trained Immunity Suppresses Ferroptotic Resolution for Severity of Inflammatory Arthritis

Ferroptosis is a programmed necrosis resulting from the accumulation of lipid peroxides and has been shown to be an important cause of arthritis, such as RA.^[^ ^32^ ^]^ Remarkably, our analysis showed that the levels of 8‐hydroxy‐2‐deoxyguanosine (8‐OHDG) and 4‐hydroxynonenal (4‐HNE), two major by‐products of lipid peroxidation, were decreased in the hyperplastic rheumatoid synovium of β‐glu‐Mφ/CIA rats, compared to PBS‐Mφ/CIA group (Figure S5A, Supporting Information). Immunofluorescence analysis indicated a decreased proportion of 4‐HNE^+^/VCAM^+^ FLS in the synovium of β‐glu‐Mφ/CIA rats (Figure S5B, Supporting Information). The expression of ferroptosis suppressor protein 1 (FSP1) was notably enhanced in VCAM^+^ FLS of the β‐glu‐Mφ/CIA group (Figure S5C, Supporting Information). These data indicate that experimentally trained immunity influences FLS ferroptosis in inflammatory arthritis.

**Figure 3:**
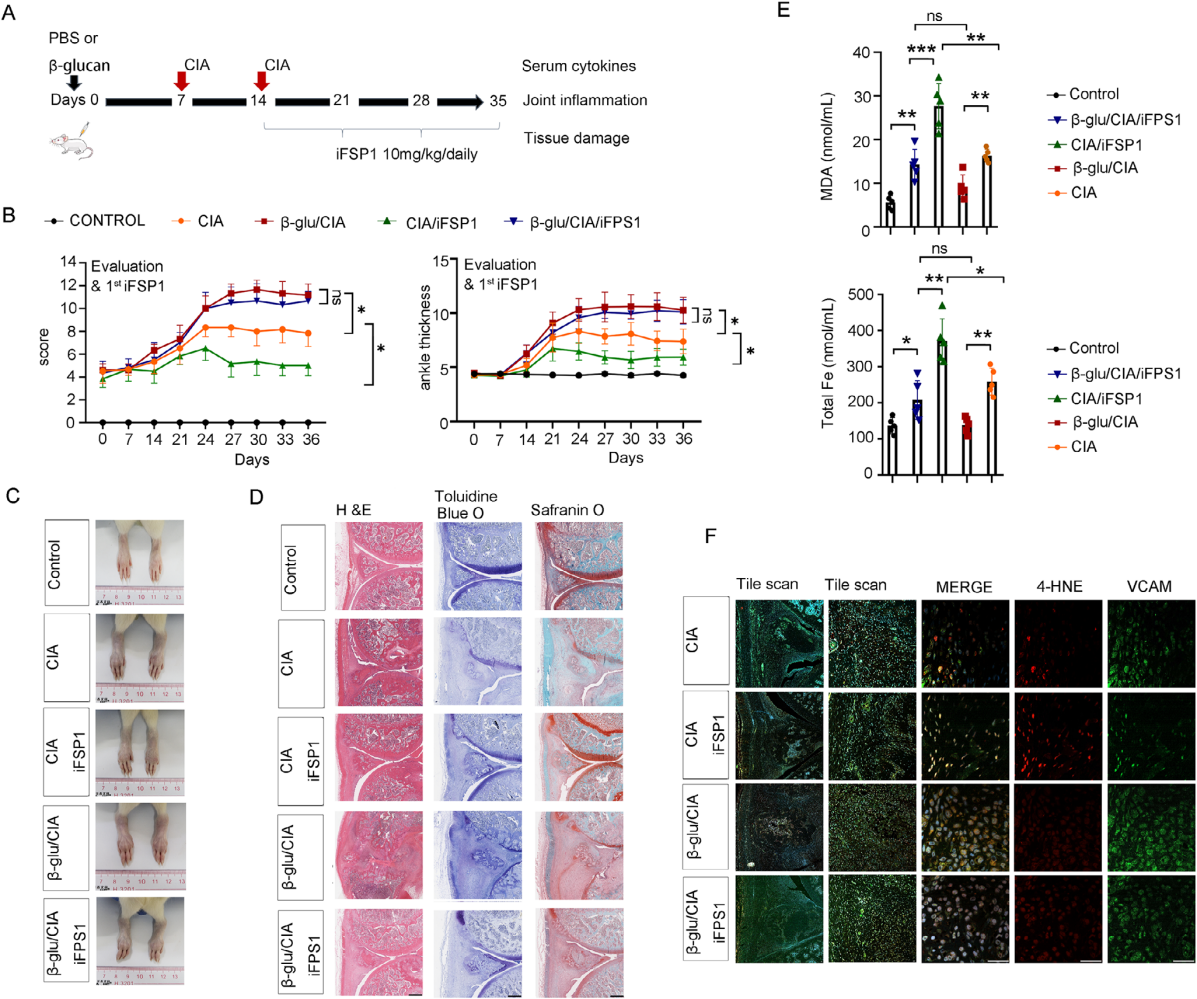
Trained immunity aggravates inflammatory arthritis in CIA rats, potentially involving ferroptosis resistance, A) Schematic representation of the experimental induction of trained immunity in CIA treated with iFSP1 (10.0 mg kg^−1^ daily), B) Arthritis index and paw thickness were scored and compared with CIA (*n* = 5), C) Representative photographs of morphology in different groups (*n* = 1), D) H&E, toluidine blue O, and safranin O staining of representative joints in different groups, scale bars, 200 µm, E) Concentration of MDA and Total iron in the joint fluid of CIA rat (*n* = 5), F) Representative immunofluorescence studies of 4‐HNE^+^VCAM^+^ cells in the joints of rats, scale bars, 50 µm, In B, E, data are presented as mean ± SD, B, ^*^ *p* <0.05 by a Two‐Way ANOVA; E, ^*^ *p* <0.05, ^**^ *p* <0.01, ^***^ *p* <0.001 by a One‐way ANOVA, ns, none sense. See also in Figures S5 and S6 (Supporting Information).

To determine whether a causal relationship between β‐glucan‐induced trained immunity and ferroptosis of FLS was involved in the pathogenesis of CIA, iFSP1 (10.0 mg kg^−1^ daily), a ferroptosis agonist that induces lipid peroxidation bursts and ferroptosis, was used to treat β‐glucan/CIA rats or PBS/CIA, respectively (**Figure** **3**A). We found that iFSP1 treatment attenuated the severity of synovial inflammation and accelerated its resolution in CIA rats. In contrast, little difference was noted in joint swelling and inflammation between β‐glucan/CIA/iFSP1 rats and β‐glucan/CIA group (Figure 3B,C; Figure S6A, Supporting Information). CIA/iFSP1 rats showed a reduced cartilage, bone damage, and pannus formation; however, the features of RA severity showed little change between β‐glucan/CIA/iFSP1 rats and β‐glucan/CIA group (Figure 3D; Figure S6B,C, Supporting Information). The levels of MDA and total Fe were lower in the joint effusion of β‐glucan/CIA/iFSP1 rats compared to CIA/iFSP1 group (Figure 3E). The alleviation of CIA symptoms caused by iFSP1 was accompanied by a marked accumulation of 4‐HNE in VCAM^+^ FLS in CIA/iFSP1 rats compared to CIA rats. Conversely, no change was observed in the synovial tissue of β‐glucan/CIA/iFSP1 and β‐glucan/CIA rats (Figure 3F). Thus, trained immunity drives the progression of inflammatory arthritis potentially through ferroptosis resistance.

### 2.4 Trained Macrophages Interacts with Arthritis Synovial FLS to Drive NAT10‐Mediated‐ ac4C Modification of FSP1 mRNA for Ferroptotic Resistance in a IL‐1β Dependent Manner

**Figure 4:**
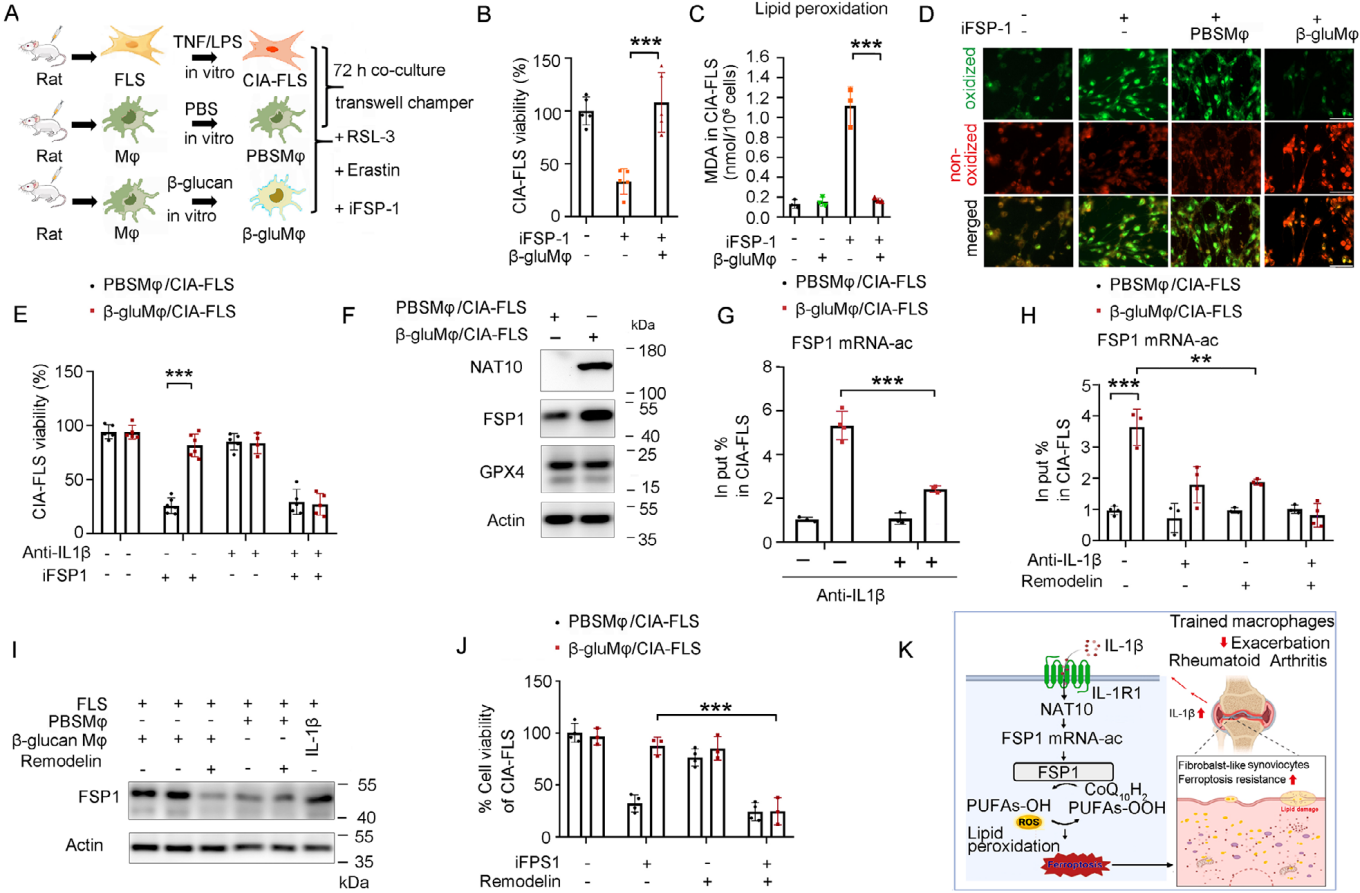
IL‐1β signal from the cross‐talk between trained macrophages and CIA‐FLS empowered CIA‐FLS a ferroptosis‐resistant phenotype, A) CIA‐FLS and PBS‐Mφ or β‐glu‐Mφ were seeded at a density of 1.0 × 10^6^ (MDA or lipid ROS) or 1.0 × 10^5^ (cell viability) cells/well on the surface of a transwell culture plate at a ratio of 1:1, and were separately co‐cultured for 72 h, CIA‐FLS then was treated with iFPS1, B) Cell viability of CIA‐FLS treated with iFPS1 (2.0 µm) was assessed by measuring CCK‐8 levels at 18 h (*n* = 5), C,D) After co‐cultured for 72 h, CIA‐FLS Cells were treated for 4 h (iFPS1, 1.0 µm), lipid peroxidation (MDA) (C) (*n* = 3) and lipid ROS accumulation (D) of CIA‐FLS was measured, E) CIA‐FLS and PBS‐Mφ or β‐glu‐Mφ were co‐cultured in the presence of anti‐IL‐1β (4.5 µg mL^−1^) for 72 h, Cell viability of CIA‐FLS was assayed after treated with iFPS1 (2.0 µm) at 18 h (*n* = 4–6), F) Western blot analysis of NAT10, FSP1, GPX4, or Actin expression in CIA‐FLS co‐cultured with PBS‐Mφ or β‐glu‐Mφ in a 1:1 ratio for 72 h (*n* = 2), G,H) Levels of ac4C FSP1 mRNA in CIA‐FLS co‐cultured with PBS‐Mφ or β‐glu‐Mφ in the presence of anti‐IL‐1β (4.5 µg mL^−1^) ± Remodelin (25.0 µm) according to A (*n* = 3–4), I) CIA‐FLS co‐cultured with trained macrophages in a 1:1 ratio for 72 h in the presence of 25.0 µm Remodelin ± 4.5 µg mL^−1^ anti‐IL‐1β, Western blot analysis of FSP1 or Actin expression, J) CIA‐FLS were separately co‐cultured with PBS‐Mφ or β‐glu‐Mφ s at a ratio of 1:1 in the presence or absence of Remodelin (25.0 µm) for 72 h, Cell viability of CIA‐FLS was analyzed followed by treatment with 2 µm iFPS1 at 18 h according to A (*n* = 3–4), K) Schematic diagram, In B, C, E, G, H, J, data are presented as mean ± SD, B, C, G ^**^ *p* <0.01, ^***^ *p* <0.01 by a One‐way ANOVA; E, H, J, ^**^ *p* <0.01, ^***^ *p* <0.01 by a Two‐Way ANOVA. See also in Figures S7–S9 (Supporting Information).

To investigate whether and how macrophage training by β‐glucan exposure affects the susceptibility of arthritis synovial FLS to ferroptosis, rat β‐glu‐Mφ were co‐cultured with rat CIA‐FLS for 72 h in vitro, and then rat CIA‐FLS were stimulated with ferroptosis inducers (**Figure** **4**A). We found that the viability of β‐glu‐Mφ/CIA‐FLS was higher than that of PBS‐Mφ/CIA‐FLS when exposed to iFSP1 (Figure 4B). The level of MDA and lipid ROS was reduced when β‐glu‐Mφ were co‐cultured with CIA‐FLS (Figure 4C,D). Additionally, crosstalk between β‐glu‐Mφ and CIA‐FLS protects arthritis synovial FLS from ferroptosis via FSP1/CoQ10 axis, not the GSH/GPX4 pathway (Figure S7A,B, Supporting Information).^[^ ^33^ ^]^

IL‐1β has been shown to prevent tumor cells from ferroptosis, and we found that IL‐1β conferred a resistance to ferroptosis in CIA‐FLS (Figure S8A–C, Supporting Information). In this context, we also observed that blocking IL‐1β, not IL‐6 or TNF, increased the ferroptosis of CIA‐FLS, which was inhibited by β‐glu‐Mφ treatment (Figure 4E; Figure S7C,D, Supporting Information). These data indicated that IL‐1β signal from the cross‐talk between trained macrophages and CIA‐FLS empowered CIA‐FLS a ferroptosis‐resistant phenotype.

We further found that β‐glu‐Mφ treatment increased the protein level of FSP1 in CIA‐FLS (Figure 4F), whereas the level of FSP1mRNA showed little change (Figure S7E, Supporting Information), as well as IL‐1β stimulation (Figure S8D,E, Supporting Information). Previously, N4‐acetylation (ac4C) of FSP1 mRNA was shown to enhance the translation efficacy of the FSP1 protein and has been associated with ferroptotic resistance.^[^ ^34^ ^]^ We observed that IL‐1β signaling originated from cell crosstalk between β‐glu‐Mφ and CIA‐FLS enhanced the enrichment of ac4C on FSP1 mRNA in CIA‐FLS (Figure 4G; Figure S8F, Supporting Information). N‐acetyltransferase 10 (NAT10) is the only nucleolar acetyltransferase known to mediate the N4‐acetylcytidine (ac4C) modification of mRNA and is crucial for the translation efficiency of FSP1.^[^ ^34^, ^35^ ^]^

Notably, macrophages training upregulated NAT10 expression in CIA‐FLS in vitro and in vivo (Figure 4F; Figure S9A,B, Supporting Information). Additionally, IL‐1β increased the protein and mRNA levels of NAT10 to promote the ac4C modification on FSP1 mRNA for the ferroptotis resistance in CIA‐FLS (Figure S8D–H, Supporting Information). Thus, we inferred that macrophage training induced IL‐1β/NAT10‐mediated ac4C modification on FSP1 mRNA, thereby protecting arthritis synovial FLS from ferroptosis. Chemical inhibition or genetic knockdown of NAT10 reversed the increased ac4C level on FSP1 mRNA (Figure 4H; Figure S9C,D, Supporting Information) and the elevated level of FSP1 protein in β‐glu‐Mφ‐primed CIA‐FLS (Figure 4I; Figure S9E, Supporting Information). Moreover, inhibition of NAT10 reduced cell viability in β‐glu‐Mφ/CIA‐FLS (Figure 4J; Figure S9F, Supporting Information). Thus, macrophage training activated the IL‐1β/NAT10/ac4C/FSP1 axis to inhibit ferroptosis of arthritis synovial FLS, indicating a causal link between trained immunity and ferroptotic resistance that contributes to the worsening of inflammatory arthritis (Figure 4K).

### 2.5 Therapeutic Targeting of Trained Immunity, Combination of Remodelin with Ferroptosis Inducer Promotes FLS Ferroptosis and Relieves Inflammatory Arthritis Symptoms

**Figure 5:**
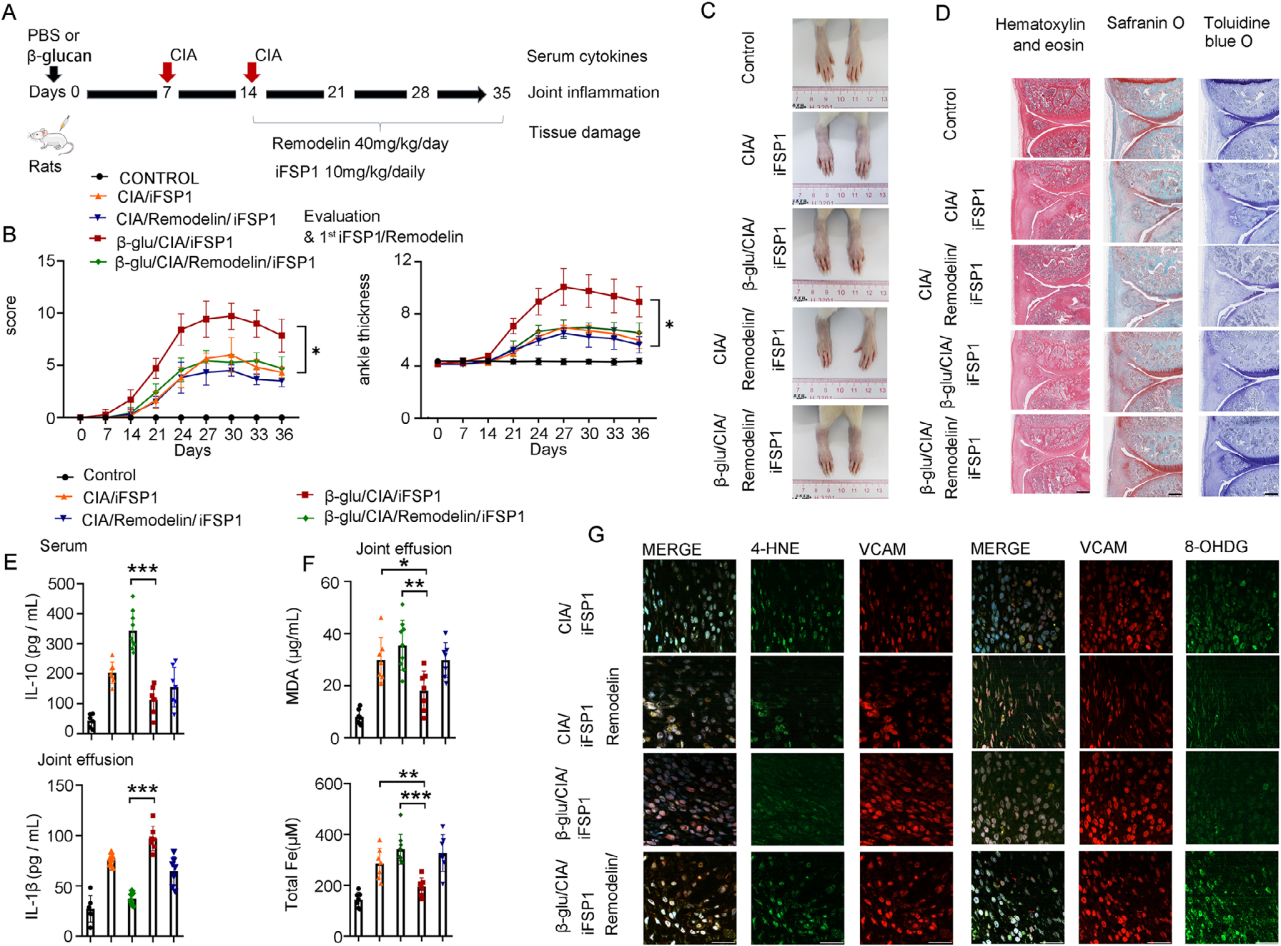
Therapeutic targeting of trained immunity, combination of Remodelin with ferroptosis inducer triggered synovial fibroblast ferroptosis and relived inflammatory arthritis symptoms, A) Experimental induction of trained immunity in CIA rat, followed by intraperitoneal injection of iFSP1 (10.0 mg kg^−1^ daily) and/or Remodelin (40.0 mg kg^−1^ daily), B) Arthritis index and paw thickness were scored in a blinded manner. ^*^Compared with CIA (^*^ *p* <0.05, ANOVA followed by multiple comparisons) (*n* = 7–9), C) Representative photographs of morphology in different groups (*n* = 3), (D) H&E, toluidine blue O, and safranin O staining of representative joints in different groups, scale bars, 200 µm, E) The levels of IL‐β and IL‐10 in the serum or joint effusion of rats (*n* = 7–9), F) Concentration of MDA and Total iron in the joint fluid of CIA rat (*n* = 7–9), G) Representative photographs of 4‐HNE^+^VCAM+ or 8‐OHDG^+^VCAM^+^ cells in the joints of rats, scale bars, 50 µm, In B, E, F, data are presented as mean ± SD, B, ^*^ *p* <0.05 by a Two‐Way ANOVA; E, F,^*^ *p* <0.05, ^**^ *p* <0.01, ^***^ *p* <0.001 by a One‐way ANOVA. See also in Figures S10 and S11 (Supporting Information).

To counteract the protective effect of trained immunity against ferroptosis and its contribution to the exacerbation of inflammatory arthritis, we explored a therapeutic intervention combining iFSP1 and Remodelin. Following macrophage training, CIA rats were treated with a low dose of iFSP1 (10.0 mg kg^−1^ daily) and/or a reduced dose of Remodelin (40.0 mg kg^−1^ daily) for 3 weeks (**Figure** **5**A). Although iFSP1 alone had little effect on the severity of joint swelling, iFSP1 combined with Remodelin alleviated the severity of synovial inflammation and blocked disease development in β‐glu/CIA rats (Figure 5B,C). Dual therapy also reduced cartilage and bone damage (Figure 5D; Figure S10A–D, Supporting Information). Additionally, the production of IL‐1β decreased, while IL‐10 levels increased in joint effusion (Figure 5E). The levels of MDA and total Fe were higher in the joint effusion of β‐glu/CIA rats treated with both iFSP1 and Remodelin than that of β‐glucan/CIA/iFSP1 rats (Figure 5F). The levels of 4‐HNE and 8‐OHDG in VCAM^+^ FLS were elevated in the hyperplastic synovium of β‐glu/CIA rats treated with both iFSP1 and Remodelin (Figure 5G; Figure S11A,B, Supporting Information). Thus, targeting the trained immunity by inhibiting NAT10 activity can synergize with ferroptotic induction to alleviate the symptoms of inflammatory arthritis.

### 2.6 CIA‐Induced Maladaptive Inflammation Contributes to an Enhanced Macrophage Training, Contributing to the Severity of Arthritis

**Figure 6:**
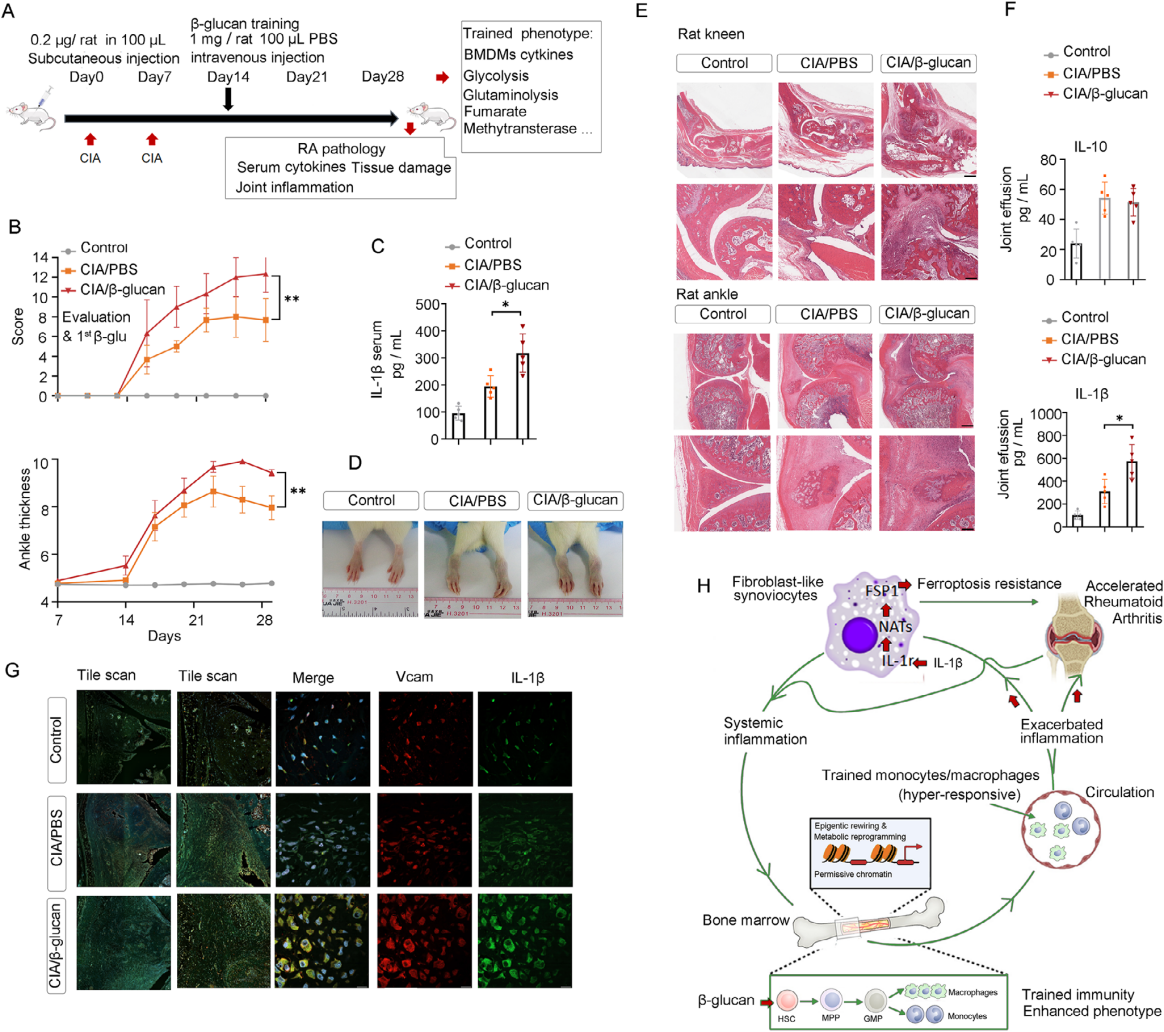
CAI‐induced maladaptive inflammation enhances macrophages training, contributing to severity of arthritis, A) CIA rat model was initiated on day 0, followed by the induction of β‐glucan‐mediated trained immunity on day 14, B) Arthritis index and paw thickness were scored. ^*^Compared with CIA (^**^ *p* <0.01, ANOVA followed by multiple comparisons) (*n* =5), C) The levels of IL‐β in the serum of rats (*n* = 5), D) Representative photographs of morphology in different groups (*n* =3), E) H&E staining of representative joints in different groups, scale bars, 200 µm, F) The level of IL‐β and IL‐10 in the joint effusion of rats (*n* = 5), G) Immunofluorescence assays of IL‐β^+^VCAM^+^ in the joints of rats, scale bars, 50 µm, H) The pathological progression of rheumatoid arthritis aggravated by trained immunity, In B, C, F, data are presented as mean ± SD, B, ^**^ *p* <0.01 by a Two‐Way ANOVA, C, F, ^*^ *p* <0.05 by a One‐way ANOVA. See also in Figures S12 and S13 (Supporting Information).

To investigate the potential bidirectional relationship between inflammatory arthritis and trained immunity, we conducted a study in rats involving β‐glucan training, following CIA induction or a control treatment (**Figure** **6**A). We found that macrophages from β‐glucan‐trained rats that underwent CIA induction (CIA/β‐glu) exhibited elevated levels of the pro‐inflammatory cytokine IL‐1β upon re‐exposure to LPS, compared to β‐glu rats (Figure S12A, Supporting Information). Metabolite accumulation was observed in the glycolysis, TCA cycle, and glutamine synthesis pathways during trained immunity (Figure S12B, Supporting Information). The levels of glutamine, lactate, and fumarate were higher in macrophages from CIA/β‐glu rats than in the other groups (Figure S12C, Supporting Information). Additionally, we observed increased activity of histone modifications H3K4me3 and H3k27ac in macrophages from the CIA/β‐glu group (Figure S12D, Supporting Information). These results indicated that CIA‐induced maladaptive inflammation contributes to enhanced macrophage training.

CIA/β‐glu rats developed more severe arthritis (Figure 6B), increased inflammation (Figure 6C), and worsened joint histopathology (Figure 6D,E; Figure S13A–C, Supporting Information), compared to untrained CIA rats. These CIA/β‐glu rats also demonstrated a marked increase in IL‐1β levels within joint effusions (Figure 6F) and a greater population of IL‐1β^+^/VACM^+^ FLS in the joints, surpassing the levels observed in β‐glu or CIA/PBS groups (Figure 6G; Figure S13D,E, Supporting Information). This evidence suggests that the maladaptive inflammation triggered by CIA potentiated the β‐glucan‐induced training of macrophages, further exacerbating the progression of inflammatory arthritis. Our findings confirm the existence of a reciprocal relationship between inflammatory arthritis and trained immunity induced by environmental factors such as β‐glucan exposure (Figure 6H), which may have implications for the pathogenesis and potential therapeutic strategies in RA and other autoimmune disorders.

## 3 Discussion, Conclusion, and Limitations

We have provided evidence that trained innate immune cells act as a central hinge perpetuating hyperinflammation through the circulation circuit between bone marrow derived trained macrophages and peripheral tissues, contributing to the development of inflammatory diseases, exemplified here by the β‐glucan‐induced trained immunity–collagen‐induced arthritis axis. In this context, we established an experimentally trained immune rat model induced by β‐glucan from *C. albicans*. We found that trained immunity enhanced the inflammatory response and exacerbated the progression of arthritis. We identified a bidirectional loop between trained immunity and inflammatory arthritis, where CIA‐induced changes in innate immune cells promote a maladaptive inflammatory phenotype, which in turn amplifies the experimental β‐glucan‐induced trained immune memory. For the first time, we report that trained immunity is linked to ferroptosis resistance. This sheds light on the induction of trained immunity by endogenous or exogenous insults, including β‐glucan exposure impacts the progression of inflammatory arthritis or other inflammatory disease.

The current study elucidates a novel, unrecognized causal link between centrally trained immunity and synovial ferroptosis resistance, an under‐investigated mechanism in the pathogenesis of inflammatory arthritis. Ferroptosis, a form of programmed cell death triggered by lipid peroxides and partially caused by abnormal inflammation,^[^ ^36^, ^37^ ^]^ is a key contributor to RA.^[^ ^27^, ^32^ ^]^ Our data indicate that the trained macrophages reduce the synovial biomarkers of lipid peroxidation and iron levels in the synovial fluid of CIA rats. Macrophage training decreased ferroptosis‐related characteristics in the synovium of CIA rats treated with iFSP1, an inducer of ferroptosis. This suggests that trained immunity enhances ferroptosis resistance, which may accelerate the onset and progression of inflammatory arthritis.

We demonstrated that trained macrophages drives FLS to ferroptosis resistance via IL‐1β signaling, where FLS acted as a secondary inflammatory trigger for the trained macrophages, increasing their activation status and IL‐1β production according to the type of the immune training. Consequently, IL‐1β signaling contributes to the resistance of FLS ferroptosis by acting as a potentiator. Recent studies have generally focused on the ferroptosis resistance of FLS, which appears to be associated with the progression of inflammatory arthritis, such as RA.^[^ ^38^ ^]^ Ling et al. found increased expression of GPX4 and SLC7A11 but decreased expression of ACSL4 in FLS from patients with RA.^[^ ^27^ ^]^ Wu et al. identified a ferroptosis‐resistant FLS subset of the synovium of patients with RA and CIA mice.^[^ ^28^ ^]^ Thus, FLS in RA exhibit ferroptosis‐resistant characteristics. We found that β‐glucan‐trained macrophages empowered CIA‐FLS with an enhanced ferroptosis‐resistant phenotype. Furthermore, IL‐1β signaling from the cross‐talk between β‐glu‐trained macrophages and FLS is the key factor in the ferroptosis resistance of FLS. Recently, TNF from the communication between macrophages and FLS provided protection against ferroptosis in a subset of FLS.^[^ ^36^ ^]^

We found that trained immunity activated an uncovered IL‐1β/NAT10/FPS1 axis to understand how FLS can withstand ferroptotic stress in joints with inflammatory arthritis.^[^ ^32^, ^39^ ^]^ IL‐1β is particularly significant in inflammation and the progression of RA.^[^ ^31^ ^]^ Most studies of IL‐1β conducted to date have focused on its regulatory effects on immune or inflammatory cells, yet the mechanisms beneath ferroptosis remain unclear. In fact, IL‐1β enhances Nicotinamide Nucleotide Transhydrogenase activity (NNT) activity through K1042 acetylation, inhibiting ferroptosis in tumor cells.^[^ ^40^ ^]^ Interleukin‐33 (IL‐33), a member of the IL‐1β family, is secreted by macrophages, and inhibits ferroptosis in endometriosis.^[^ ^41^ ^]^ We found that stimulation of IL‐1β augmented the expression of NAT10 and ac4C on FSP1 mRNA in FLS. While FSP1 is recognized to suppress ferroptosis through CoQ10 regeneration and NADPH pathway modulation,^[^ ^34^, ^35^ ^]^ our study reveals an upstream epigenetic control point where IL‐1β/NAT10 axis increased the ac4C modification of FSP1 mRNA, thereby amplifying its cytoprotective effects. NAT10/FSP1 signaling induces ferroptosis resistance via ac4C modification in colon cancer cells.^[^ ^34^, ^35^ ^]^ Although we establish that NAT10‐driven FSP1 upregulation is critical for ferroptosis resistance, a detailed investigation of downstream FSP1 effectors (e.g., the CoQ10‐GPX4 axis vs alternative pathways) remains an important future direction.

We have established a therapeutic approach for inflammatory arthritis development accelerated by endogenous or exogenous insults, including β‐glucan exposure, which addresses both trained immunity and ferroptosis. Strategies targeting the reversal of trained immunity‐induced changes offer opportunities for the development of promising therapeutic tools for treating chronic inflammatory diseases.^[^ ^24^, ^42^, ^43^ ^]^ However, the targeting of trained immunity to counteract inflammatory arthritis remains poorly understood. Ferroptosis has been suggested as a therapeutic target for inflammatory arthritis, and several anti‐rheumatic drugs have been associated with ferroptosis resistance.^[^ ^32^ ^]^ However, few in vitro and animal models have shown their implications in the pathogenesis of inflammatory arthritis.^[^ ^44^, ^45^ ^]^ We found that trained immunity by β‐glucan induced the ferroptosis resistance in FLS to worsen inflammatory arthritis progression through IL‐1β/NAT10/FPS1 axis. Thus, targeting ferroptosis resistance mediated by trained immunity, with a combination of a lower dose of iFSP1 and a reduced dose of Remodelin, can induce ferroptosis in FLS and suppress inflammatory arthritis progression deteriorated by β‐glucan exposure. Moreover, a neutralizing anti‐IL‐1β monoclonal antibody has demonstrated efficacy in clinical trials for inflammatory diseases^[^ ^46^ ^]^; however, the role of IL‐1β inhibition in inflammatory disease therapy remains fully unexplored. We found that blocking IL‐1β disrupts the NAT10/FSP1 signaling, thereby reversing the ferroptosis‐resistant phenotype of FLS, which offers insights into the molecular basis of blockage of IL‐1β in the treatment of inflammatory arthritis.

In conclusion, our data indicates that trained immunity induced by β‐glucan insult exacerbates the severity of inflammatory arthritis by disrupting the normal ferroptosis of synovial FLS. Under ferroptosis stress, IL‐1β signaling, arising from the cross‐talk between trained macrophages and CIA‐FLS, inhibits ferroptosis in FLS. Heterogeneous resistance to ferroptosis in CIA‐FLS, mediated by NAT10‐induced ac4C modification of FSP1 mRNA, leads to subsequent immune dysregulation and exacerbates joint degradation in CIA. By therapeutically targeting trained immunity, combining a NAT10 antagonist with a low dose of iFSP1 (a ferroptosis inducer) restores ferroptosis in FLS and alleviates the progression of arthritis under macrophage training. Our findings highlight how anterior antigenic exposure caused by acute or chronic infections can shape the immune system toward an immunostimulatory status through trained immunity, which could influence both the onset and progression of inflammatory arthritis or other autoimmune diseases, implicating trained immunity.

First, while serum and synovial factors in patients with RA (e.g., HMGB1 or plate‐bound IgG) are known to induce trained immunity, whether they regulate arthritis progression via mechanisms similar to β‐glucan requires further investigation. Second, whether the phenotype we observed in β‐glucan‐trained macrophages exists in primary macrophages derived from patients with RA PBMCs and whether they similarly influence patient‐derived FLS in vivo remains unclear. Third, given the heterogeneity of synovial fibroblasts, future work should determine if the IL‐1β/NAT10/FSP1 axis operates preferentially in FLS subsets with distinct ferroptosis sensitivities. Fourth, although we established the role of trained immunity in FLS ferroptosis resistance, its relationship with ferroptosis in other joint cells (e.g., chondrocytes and osteoclasts) remains unknown. Finally, single‐cell approaches (ATAC‐seq and metabolomics) of hematopoietic progenitors could reveal how inflammatory arthritis sustains trained immunity, a question that extends to other inflammatory diseases.

## 4 Experimental Section

### Induction of Trained Immunity in a CIA Rat Model

Male Wistar rats, aged 6–8 weeks and weighing 180–200 g on average, were purchased from Sibeifu (Beijing) Biotechnology Co., Ltd. under license no: SCXK (Beijing, China) 2019–0010. All animals were maintained under constant standard environmental conditions (23 ± 1 °C, 55 ± 5% humidity, and a 12/12 h light/dark cycle) for 1 week prior to the experiments. For Figure 1A, rats were trained with β‐glucan (1.0 mg rat^−1^, Invivogen, #tlrl‐wgp) in 100.0 µL PBS by intravenous route on day 0, and then injected subcutaneously with 200.0 µL emulsion (equivalent amounts of incomplete Freund's adjuvant (Chondrex, Redmond, USA) and bovine type II collagen (Chondrex, Redmond, USA)) (0.2 µg rat^−1^) unilaterally at the base of the tail on day 7. On day 14, 100.0 µL of emulsion and bovine type II collagen was applied to the other side. The control group (PBS mice) received equal volumes of sterile PBS. On day 35, the remaining animals were euthanized to assess inflammatory arthritis severity markers.

The development of arthritis was monitored, and the arthritis score was assessed every 3 days.^[^ ^47^ ^]^ The degree of inflammation for each paw was scored from 0 to 4 according to the following scale: 0 = no inflammation, 1 = paw with detectable swelling in a single digit, 2 = paw with swelling in more than one digit, 3 = paw with swelling of all digits and the instep, and 4 = severe swelling of the paw and ankle. The arthritic scores of the four paws were summed. At the end of the study, blood samples were taken from mice under deep anaesthesia, and all the mice were euthanized by an intraperitoneal injection of pentobarbital.

For Figure 3A, the establishment of a trained immunity model (β‐glucan or PBS) was initiated on day 0, the establishment of a CIA rat model was initiated on day 7, CIA was re‐enforced on day 14, and ferroptosis induction was initiated by daily intraperitoneal injection of iFSP1 (10.0 mg kg^−1^ daily, MedChemExpress, # HY‐1360571) on day 14, the experiment was terminated on day 35.

For Figure 5A, the establishment of a trained immunity rat model (β‐glucan or PBS) was started on day 0, the establishment of a CIA rat model was started on day 7, CIA was re‐enforced again on day 14, and ferroptosis induction was started by daily intraperitoneal injection of iFSP1 (10.0 mg kg^−1^ daily) on day 14 in the presence or absence of Remodelin (40.0 mg kg^−1^ daily, MedChemExpress, #HY‐16706), the experiment was terminated on day 35.

For Figure 6A, the establishment of a CIA rat model was initiated on day 0, CIA was re‐enforced again on day 7, followed by the establishment of β‐glucan trained immunity on day 14.

### Adoptive Transfer of Trained Macrophages in CIA Rats

For Figure 2A, the establishment of a trained immunity model (β‐glucan or PBS) was started on day ‐7, trained macrophages were isolated from BMDMs of rats on day 7. Wistar rats (male, 6–8 weeks, 180–200 g) were injected subcutaneously with 200.0 µL of emulsion and bovine type II collagen (0.2 µg rat^−1^) unilaterally at the base of the tail on day 0. On day 7, 100.0 µL of emulsion and bovine type II collagen was were applied to the other side. On day 7 and 14, Control or CIA rats received a weekly intraperitoneal injection of 200.0 µL sterile PBS, β‐glu‐Mφ/CIA rats received the injection of 1.0 x 10^7^ β‐glucan‐trained macrophages in 200.0 µL of sterile PBS, PBS‐Mφ/CIA rats received an injection of 1.0 × 10^7^ PBS‐treated macrophages (untrained macrophages) in 200.0 µL of sterile PBS. On day 35, the animals were killed, and analysis of the inflammatory arthritis severity markers was performed.^[^ ^8^ ^]^

### Training of Bone Marrow‐Derived Macrophages In Vitro

Macrophages (1.0 × 10^6^) were stimulated with Gibco RPMI 1640 medium or β‐glucan at 5.0 µg mL^−1^ for 24 h in the presence of recombinant granulocyte‐macrophage colony‐stimulating factor (GM‐CSF) at 40.0 ng mL^−1^. The cells were then washed and rested in the culture medium containing 10% fetal bovine serum for 6 days. All cells were cultured in complete RPMI 1640 medium supplemented with 10% FCS and incubated at +37 °C in an atmosphere containing 5% CO2. Cells were then harvested by brief trypsin‐EDTA (Gibco) treatment, counted, and used either for co‐culture experiment or intraperitoneal injection.

### Rat Trained Macrophages–Fibroblast‐Like Synoviocytes Co‐Culture

Fibroblast‐like synoviocytes isolated from rats were stimulated with 10.0 ng mL^−1^ TNF‐α and 10.0 ng mL^−1^ LPS for 24 h to generate transformed CIA‐FLS. These cells and trained macrophages (in vitro training) were seeded at a density of 1.0 × 10^5^ cells/well on the surface of a transwell culture plate at a ratio of 1:1, and were separated using a co‐culture chamber. Trained macrophages were placed in the upper chamber, and fibroblast‐like synoviocytes were located in the lower chamber. Trained macrophages were cultured alone in complete RPMI medium to assess the basal activation status. Fibroblast‐like synoviocytes were seeded in DMEM supplemented with 10% FBS, 1% streptomycin‐penicillin, and 1% of ciprofloxacin. The cells were then incubated for 72 h at 37 °C in 5% CO2. Supernatants were collected and stored at −80 °C for further cytokine measurement.^[^ ^8^ ^]^

### ac4C RNA Immunoprecipitation (RIP) Assay and RT‐Qpcr

ac4C‐RIP was performed using an RNA immunoprecipitation kit (Genelily Biotech, #GK‐5044) according to the manufacturer's instructions. Briefly, a total of 10 µg anti‐ac4C antibody (Abcam, #ab252215,) or anti‐rabbit IgG antibody was pre‐incubated with 50 µL Protein A/G magnetic beads in IP buffer (150 mm NaCl, 0.1% NP‐40, 10 mm Tris‐HCl, pH 7.4) at room temperature for 0.5 h. Then, 300 µg of RNA fragments were added to the pre‐incubated beads and incubated at 4 °C for 3 h. After washing the beads with buffer, the RNA was extracted, and then the bound ac4C‐modified RNA was eluted for analysis by RT‐qPCR as previously described.^[^ ^34^ ^]^ Equal amounts of RNA fragments (not subjected to immunoprecipitation) were used as the input controls.

### MDA Measurement in Cells

CIA‐FLS and PBS‐Mφ or β‐glu‐Mφ were seeded at a density of 1.0 × 10^6^ cells well^−1^ on the surface of a transwell culture plate at a ratio of 1:1, and were separately co‐cultured for 72 h, CIA‐FLS were then treated for 4 h (iFPS1, 1.0 µm), and lipid peroxidation (MDA) of CIA‐FLS was measured. The MDA assay kit (Beyotime; #S0131S) was used to detect the concentration of intracellular MDA. According to the manufacturer's in‐structions, colorimetry was used to detect MDA in cell lysates based on the color reaction of MDA and thiobarbituric acid (TBA). The MDA level was measured by Multi‐Mode Microplate Readers (BioTek Instruments; Thermo Fisher Scientific) at 532 nm.

### Data Treatment and Statistical Analysis

Data were analyzed using GraphPad Prism 8.01 software. Data were presented as mean ± standard deviation except were stated otherwise. All experiments that underwent error analysis were performed using two or more independent measurements. Statistical analyses were performed using an unpaired two‐tailed Student's *t*‐test for two‐group comparisons. When variances were not equal or the data were ranked, the Mann–Whitney test was applied. One‐way ANOVA was used to confirm significant main effects and differences among three or more groups, followed by Tukey's multiple comparisons for post‐hoc tests. For the grouped data, two‐way ANOVA was used to analyze the levels of significant main effects, followed by Bonferroni's multiple comparisons for post‐hoc tests. Survival curves were compared using the log‐rank (Mantel–Cox) test. For all experiments, ^*^ *p* <0.05, ^**^ *p* <0.01, and ^***^ *p* <0.001 were considered significant. Sample size: Explicit ^*^ *n* ^*^values provided for each experiment (e.g., ^*^ *n* ^*^ = 3 biological replicates) in the Figure legends.

### Ethical Approval Statement

All animal experiments should comply with the National Institutes of Health guide for the care and use of Laboratory animals (NIH Publications No. 8023, revised 1978). Animal experiments were performed as per ethical guidelines according to the rules of the Committee on Animal Research and Ethics, this research project has been approved by the Institutional animal care and use committee (GY‐2023‐259), Guangzhou Medical University, China.

## Conflict of Interest

The authors declare no conflict of interest.

## Author Contributions

H.S., B.Z., Q.D., and J.H. contributed equally to this work. HS and JD, resources; QD, BZ, JH, data curation; QD, BZ, JH, software; QD, BZ, JH, formal analysis; BZ, JH, JF, YH, YF, JS and WD, validation; BZ, JH, YH, JF, YF, YH investigation; HS, QD, BZ, JH, ZZ and JD, visualization; QD, BZ, JH, YF, YH, methodology; HS and QD, writing of the original draft; HS and JD, writing the review and editing; HS and JD, funding acquisition; HS, NS, HL, ZZ and QD, project administration; HS, QD, ZZ and JD, conceptualization; HS, NS, HL, ZZ and JD, supervision. The order among co‐first authors is determined by their contribution to performing experiments and writing the manuscript.

## Supporting information



Supporting Information

## Data Availability

The data that support the findings of this study are available in the supplementary material of this article.
