# Supplementary material for: Trained Immunity Exacerbates Inflammatory Arthritis Progression via Promoting Synovial Fibroblast Ferroptotic Resistance
Source: Adv Sci (Weinh). 2025 Nov 19;12(46):e04245. doi: 10.1002/advs.202504245 (PMC12697851; doi:10.1002/advs.202504245)

Supporting Information

Trained Immunity Exacerbates Inflammatory Arthritis Progression via Promoting Synovial Fibroblast Ferroptotic Resistance

Haibo Su[1#*], *Baoying Zhang*[1#], *Qiudi Deng*[1#], *Jiaxin Huang*[1#], *Jinyu Feng*[1], *Yuan Fu*[1], *Yuejun Huang*[1], *Weikun Deng*[1], *Junying Su*[1], *Huisheng Liu*[2,3], *Ning-yi Shao*[4], *Zhenhui Zhang*[1*], *Jianwei Dai*[1*]

[1] Department of Critical Care Medicine, GMU-GIBH Joint School of Life Sciences, the Guangdong-Hong Kong-Macao Joint Laboratory for Cell Fate Regulation and Diseases, State Key Laboratory of Respiratory Disease, Guangzhou Key Laboratory of Prevention and Treatment of Multiple Organ Dysfunction Syndrome, the Second Affiliated Hospital, Guangzhou Medical University, Guangzhou, 510260, China.

[2] Guangzhou National Laboratory, Guangzhou 510006, China.

[3] School of Biomedical Engineering, Guangzhou Medical University, Guangzhou 510180, China.

[4] Department of Biomedical Sciences, Faculty of Health Sciences, University of Macau, Taipa, Macau Special Administrative Region of China 999078, China.

[#] The authors contributed equally to the work

*To whom Correspondence should be addressed:

Haibo Su: Department of Critical Care Medicine, GMU-GIBH Joint School of Life Sciences, the Guangdong-Hong Kong-Macao Joint Laboratory for Cell Fate Regulation and Diseases, State Key Laboratory of Respiratory Disease, Guangzhou Key Laboratory of Prevention and Treatment of Multiple Organ Dysfunction Syndrome, the Second Affiliated Hospital, Guangzhou Medical University, Guangzhou, 510260, China.

E-mail: suhaibo899@gzhmu.edu.cn.

Zhenhui Zhang: Department of Critical Care Medicine, GMU-GIBH Joint School of Life Sciences, the Guangdong-Hong Kong-Macao Joint Laboratory for Cell Fate Regulation and Diseases, State Key Laboratory of Respiratory Disease, Guangzhou Key Laboratory of

Prevention and Treatment of Multiple Organ Dysfunction Syndrome, the Second Affiliated Hospital, Guangzhou Medical University, Guangzhou, 510260, China.
E-mail: zzhmed@gzhmu.edu.cn.
Jianwei Dai: Department of Critical Care Medicine, GMU-GIBH Joint School of Life Sciences, the Guangdong-Hong Kong-Macao Joint Laboratory for Cell Fate Regulation and Diseases, State Key Laboratory of Respiratory Disease, Guangzhou Key Laboratory of Prevention and Treatment of Multiple Organ Dysfunction Syndrome, the Second Affiliated Hospital, Guangzhou Medical University, Guangzhou, 510260, China.
E-mail: daijw@gzhmu.edu.cn.

Funding: This work was supported by the National Natural Science Foundation of China (Grant Numbers: 82271871, 42130611, 32270790, 82300056, 82104492); the Natural Science Foundation of Guangdong Province, China (Grant Number: 2021A1515010913); Project of the State Key Laboratory of Respiratory Disease, Guangzhou Medical University (Grant Numbers: SKLRD-Z-202223, SKLRD-Z-202303, SKLRD-OP-202214, SKLRD-OP-202303); Tertiary Education Scientific research project of Guangzhou Municipal Education Bureau (Grant Number: 202235403); R&D Program of Guangzhou laboratory (Grant Number: SRPG22–021); GMU-GIBH Joint School of Life Sciences, the Guangdong-Hong Kong-Macao Joint Laboratory for Cell Fate Regulation and Diseases, Guangzhou Medical University (Grant Number: 2022LSYS008); Special Project of Guangdong Province Education Department in Key Fields (Grant Numbers: 2022ZDZX2050); Guangzhou Key Laboratory of Prevention and Treatment of Multiple Organ Dysfunction Syndrome(Grant Numbers: 2025A03J3350) the Open Research Funds from the Sixth Affiliated Hospital of Guangzhou Medical University, Qingyuan People's Hospital, China (Grant Numbers: 202201-2011, 202301-307).

Keywords: trained immunity, innate immune memory, trained macrophages, inflammatory arthritis, ferroptosis

This file contains: Supplementary Methods, Supplementary Figures S1–S13, Ethic approval, Raw data-blots, Raw data-Paws microscope images, Raw data-H&E microscope images, Raw data- Safraing staining microscope images, Raw data- Tuliudine Blue O staining microscope images, Raw data-Immunohistochemical microscope images, Raw data- Immunofluorescent staining microscope images.

1. Methods and Materials

Immunofluorescence and Immunohistochemical staining

After the samples were washed with PBS three times, sample or slices were permeabilized with 0.1% Triton X-100 for 30 min, blocked with 5% bovine serum albumin (BSA) for 2 h at room temperature and probed with primary antibodies overnight at 4 °C. The primary antibodies targeted the following proteins or modifications: 8-OHdG (1:500, Abcam, #ab48508), 4-HNE (1:500, Abcam, #ab48506), F4/80(1:1000, Abcam, #ab300421, Santa cruz, #sc-377009), VCAM-1(1:1000, Abcam, #ab134047, ab316315, Proteintech, #66294-1-Ig), IL-1β (1:500, Novus, #IMG-6826A), NAT10 (1:1000, Abcam, #ab194297), Ki-67 (1:1000, Abcam, #ab279653), AIFM2/FSP1 (1:500, Cell Signaling Technology, #24972). Then, the cells were exposed to secondary antibodies (Alexa Fluor® 568(1:2000, Abcam, #ab175470, #ab175473) or Alexa Fluor® 488(1:2000, Abcam, #ab15007, #ab150113) and stained with 4, 6-diamidino-2-phenylindole (DAPI). Images were acquired using a confocal laser scanning microscope (LSM800, ZEISS, Germany).[1]

Immunohistochemical staining was performed using a streptavidin–peroxidase kit (ZSGB-Bio, China). slices were permeabilized with 0.1% Triton X-100 for 30 min, blocked with 5% bovine serum albumin (BSA) for 2 h at room temperature and probed with primary antibodies overnight at 4 ◦C. Then, the slices were exposed to secondary antibodies rabbit IgG Isotype Control (Invitrogen, #31235), and mouse IgG Isotype Control (Invitrogen, #14-4714-82) with a standard avidin-biotin HRP detection system according to the instructions of the manufacturer (HRP-DAB Cell & Tissue Staining Kit, R&D Systems, Minneapolis, MN).[2]

Bone marrow derived macrophage isolation and culture

Bone Marrow Derived Macrophages (BMDMs) were cultured using the medium (RPMI with 20% L929 cell supernatant containing GM-CSF required to macrophage differentiation, 10% heat inactivated Fetal Bovine Serum (FBS), 1% antibiotic/antimycotic, 1% HEPES, and 1% sodium pyruvate) (Eurobio Ingen. Les Ulis, France). At day 7, cells were removed from plastic dishes by incubation in $Ca2^{+}$/$Mg2^{+}$-free PBS and collected by brief trypsin-EDTA (Gibco) treatment. Cells were spun at 1000 rpm for 10 min to form a pellet and then re suspended in complete RPMI media.

Isolation of fibroblast-like synoviocytes

Synovial tissue of rats were harvested and minced into small pieces. Synovial fibroblasts (RASFs) were isolated by digestion with 2 mg/ml collagenase (Sigma-Aldrich Ref C2674) and

1.0 mg/ml of dispase (Liver Digest Medium, Gibco) for 4 h at 37 °C. The cell suspension was then filtered in a 100-µm cell strainer, washed three times in DMEM and cultured in the presence of DMEM supplemented with 10% FBS, 2% L-glutamine (Gibco), 1% streptomycin-penicillin, and 1% of Ciprofloxacin (Panpharma 2 mg/ml) at 37 °C in a humidified atmosphere of 5% CO2. When the cells had grown to 80% confluence, they were detached with 0.25% trypsin, split in a 1:3 ratio, and reseeded in DMEM under the same conditions. To eliminate nonadherent cells, the plated cells were washed thoroughly with PBS. Isolated fibroblast-like synoviocytes (FLSs) were cultured in DMEM supplemented with 10% FBS. After ~1 week incubation at 37 °C in an atmosphere of 5% CO2, fibroblasts were detached by brief trypsin-EDTA (Gibco) treatment and resuspended in DMEM. Collected fibroblast were counted and used for co-culture experiments.

Human β-glu-hMφ and RA-hFLS co-culture

hFLS cells were stimulated with 100 ng/mL LPS and 10 ng TNF-α for 24 h to produce transformed RA-hFLS. Thp-1 in vitro training was performed by 10 µg/mL for 24 h treatment to generate β-glu-hMφ. RA-hFLS and β-glu-hMφ were seeded on the surface of a transwell culture plate at a ratio of 1:1, and were separated using a co-culture chamber. β-glu-hMφ were placed in the upper chamber, and RA-hFLS were located in the lower chamber. β-glu-hMφ were cultured alone in complete RPMI medium to assess the basal activation status. RA-hFLS were seeded in DMEM supplemented with 10% FBS, 1% streptomycin-penicillin and 1% of ciprofloxacin. The cells were then incubated for 72 h at 37 °C in 5% CO2. Supernatants were collected and stored at −80 °C for further cytokine measurement

Measurement of MDA and iron level in synovial fluid

Synovial fluid samples from CIA rats were centrifuged at 1000 × g for 10 min and filtered through a 0.2 µm filter to remove cells and debris. The intracellular MDA levels were assessed using the lipid peroxidation MDA Detection Kit (Beyotime, #S0131M) following the manufacturer’s instructions. In brief, a thiobarbituric acid (TBA) solution was incubated with synovial fluid samples and MDA standards at 100 °C for 15 min. The MDA-TBA adduct was quantified colorimetrically (OD = 532 nm).

Total iron levels in synovial fluid samples of CIA rats were assessed by the Iron Assay Kit (Abcam, #ab83366) according to the manufacturer’s instructions. In brief, an iron reducer was added to the sample and standard wells. The mixture was incubated at 37 °C for 30 min, and iron was added and incubated for an additional 60 min. The output was assessed on a

colorimetric microplate reader (OD = 593 nm).

ELISA cytokine detection

Assessment of IL-1β (Abcam, #ab255730), IL-10 (Abcam, #ab214655), TNF-α (Abcam, #ab236715), MMP2 (Abcam, #ab213910), MMP9 (Invitrogen, #EEL130), IL-6 (Abcam, #ab234570), MCP1 (Abcam, #ab219045), MIP-3-β (Biosciences, #IT7499) was performed by specific rat ELISA kits. Concentrations were calculated from a standard curve according to the manufacturer's protocol.[3]

Flow Cytometry

Central or peripheral immune cells were used for M1 or M2 ratio statistics by flow cytometry. Cells were then incubated with the appropriate labeled antibody at 4 °C for 30 min in the dark in PBS with 2% normal FBS. Flow cytometry was performed using a FACS Fortessa II flow cytometer (BD Biosciences), according to standard techniques. For the characterization of splenic, blood, peritoneal and bone marrow derived macrophages, the antibodies used were: Anti-Rat CD11b (1:50, BD Biosciences, #562108,), Anti-Rat CD86 (1:50, BD Biosciences, #551396), Anti-Rat CD206 (1:50, Santacruz, #sc-376108). Cells were washed twice and red blood cells were lysed in ACK buffer (eBioscience). After two additional washes, cells were resuspended in FACS buffer and analyzed via FACSCalibur (BD Biosciences).

Western blotting

For Western blot analysis, we used a BCA Protein Assay Kit (Beyotime, China) to measure the protein concentration. The proteins were mixed with sample buffer, boiled at 100 ◦C for 7 min for denaturation, and separated via 10% SDS-PAGE. The proteins were transferred onto nitrocellulose membranes by a membrane transfer system (Bio-Rad, USA). Then, 5% milk was used to block the membranes for 2 h at room temperature. The membranes were incubated overnight at 4 ◦C with the following primary antibodies: GAPDH (1:1000, Cell Signaling Technology, #97166), GPX4 (1:1000, Proteintech, #67763-1-Ig), NAT10 (1:1000, Abcam, #ab194297), AIFM2/FSP1 (1:1000, Cell Signaling Technology, #24972), β-actin (1:2000; Proteintech, #66009-1-lg). The membranes were washed with TBS-Tween and incubated with the proper secondary antibody. The blots were visualized by a chemiluminescence detection system.[4]

Methtransferase and Demethtransferase Activity Assay

Nuclear extract was performed by EpiQuik Nuclear Extraction Kit *(*Epigentek, #OP-0002-01*).* Briefly, 1 × 10^7 cell were resuspended in 500 µl cell lysis buffer (10 mM HEPES; pH 7.5, 10 mM KCl, 0.1 mM EDTA, 1 mM DTT, 0.5% Nonidet-40, and 0.5 mM PMSF along with the protease inhibitor cocktail [Sigma]) and allowed to swell on ice for 20 min with intermittent mixing. Tubes were vortexed and then centrifuged at 12,000 × *g* at 4°C for 10 min. The pelleted nuclei were washed twice with the cell lysis buffer and resuspended in 25 µL ice-cold nuclear extraction buffer (20 mM HEPES [pH 7.5], 400 mM NaCl, 1 mM EDTA, 1 mM DTT, and 1 mM PMSF with protease inhibitor cocktail) and incubated in ice for 30 min with intermitted sonication. Nuclear extract was collected by centrifugation at 12,000 × *g* for 15 min at 4°C. The supernatant was used immediately in a fluorometric Activity Quantification Assay Kit (Abcam), performed following the instructions of the company (KDM6/JARID Activity Quantification Assay Kit, Abcam, #ab156910; KDM5/JARID Activity Quantification Assay Kit, Abcam, #ab113464; Histone H3 (K9) Methyltransferase Activity Quantification Assay Kit, Abcam, #ab113453; Histone H3 (K4) Methyltransferase Activity Quantification Assay Kit; Abcam, #ab113452; Histone H3 (K27) Methyltransferase Activity Quantification Assay Kit; Abcam, #ab113454; Histone Acetyltransferase Activity Assay Kit, Abcam, #ab204536).[5]

Metabolic status analysis

Quantification Colorimetric Kit from the cell lysate according to manufacturer's protocol. At least one million cells were lysed in 100 µL 0.5% Triton-X in PBS. Metabolite concentrations were determined by commercial assay kits for succinate, fumarate, glutamate, malate, α-ketoglutarate, following the instructions of the manufacturer. All of these assay kits were obtained (Lactate Assay Kit, Sigma-aldrich, #MAK064; Glutamine Assay Kit; Sigma-aldrich, # MAK438-1KT; Fumarate Assay Kit, Sigma-aldrich, #MAK060-1KT; Alpha Ketoglutarate (alpha KG) Assay Kit, Abcam, #ab83431; NADP/NADPH Assay Kit, Sigma-aldrich, # MAK479-1KT; NAD/NADH Assay Kit, Sigma-aldrich, # MAK468-1KT; Glucose Uptake Assay Kit, Abcam, #ab136956).[6]

Neutralizing and Priming assay

For in vitro neutralization, 4.5 µg/mL anti-IL-1β neutralizing antibody (Thermofisher, #16-7012-81) with or without 10.0 ng/ml rat recombinant IL-1β (R&D Systems, #501-RL-050/CF). 3.5 µg/mL anti-IL-1R (Thermo Fisher Scientific, #PA5-47661) + 3.5 µg/mL anti-IL-1R (Abcam, #ab253848); 3.5 µg/mL anti-IL-6R (Thermo Fisher Scientific, #16-1261-85) + 3.5 µg/mL anti-IL-6R (R&D Systems, # AF-227-SP); 3.5 µg/mL anti-gp130 (Thermo Fisher Scientific, #MA5-

23817) + 3.5 μg/mL anti-gp130 (Abcam, #ab283713); 3.5 μg/mL anti-TNFR1 (Thermo Fisher Scientific, #16-1202-81) + 3.5 μg/mL anti-TNFR1 (Abcam, # ab244075); 4.5 μg/mL anti-TNFR2 (Thermo Fisher Scientific, #MA5-29837). For in vitro priming, 10.0 ng/ml rat recombinant IL-1β, 10.0 ng/ml rat recombinant IL-6 (R&D Systems, #506-RL-050/CF), or 5 ng/ml rat recombinant TNF-α (R&D Systems, #510-RT-050/CF) was used.[2,7]

Trained immunity in vivo models

Wistar rats, male, 8 ~ 12 weeks old, were trained with intravenous injections of 1.0 mg/rat of β-glucan particles at week 0, then lethally infected with *C. albicans* at ~ 3×10^7 cfu intravenously at week 4. PBS was used as the control. 3 or 7 days later, rats BMDMs were challenged with 10 μg of LPS i.p., and blood was collected to assess serum TNFα, IL-1β, and IL-6 levels. Alternatively, rats were monitored daily for general health and survival, following institutional guidance. Data presented are the combined survival data (Kaplan-Meier) from 2 independent experiments. A log-rank test was used to assess the statistical significance between the groups.

Hematoxylin-eosin staining, Toluidine Blue O and Safranin-O assay

The ankle specimens were fixed in 4% paraformaldehyde for 48 h and sectioned after calcium removal for 45 days. Each glass slide was stained with H&E, toluidine Blue O or safranin O for general histological evaluation.

Measurement of cell viability and lipid ROS in cells

Cell viability was as-sessed by the Enhanced Cell Counting Kit-8 (Beyotime; C0041). CIA-FLS and PBS-Mφ or β-glu-Mφ were seeded on the surface of a transwell culture plate at a ratio of 1:1, and were separately co-cultured for 72 h, CIA-FLS then was treated with iFPS1 (2 μM). Cell viability of CIA-FLS was assessed by measuring CCK-8 levels at 18 h. The supernatant was removed and 100 μL of a complete medium containing 10 μL CCK-8 solution was added to each well. The plates were incubated at 37 °C for 1 h. A microplate reader (BioTek Instruments; Thermo Fisher Scientific) was used to measure the absorbance at 450 nm. For analysis of lipid ROS, cells were stained with 5 μM BODIPY-C11 (Invitrogen) for 30 min at 37 °C by confocal laser microscopy.

Actinomycin D treatment

Transcription was blocked by adding actinomycin D at 5 μg/mL (GlpBio, #GC16866) to the

cell culture 30 medium for 0 h, 3 h, 6 h, 9 h and 12 h.

Real-time quantitative PCR (RT-qPCR)

Total mRNA was extracted using a FastPure Cell/Tissue Total RNA Isolation Kit V2 (Vazyme, #RC112-01). The concentration and purity of mRNA was assessed using a Nanodrop spectrophotometer. Isolated mRNA (1 μg) was subjected to reverse transcription-polymerase chain reaction (PCR) to generate cDNA using the Evo M-MLV Reverse Transcription Reagent Premix (Accurate Biology, #AG11706). cDNA was then subjected to RT-qPCR using the SYBR Green Pro Taq HS premixed qPCR Kit (Accurate Biology, #AG11701). Glyceraldehyde-3-phosphate dehydrogenase (GAPDH) was used as an internal control. Relative gene expression was normalized to GAPDH mRNA expression and analyzed using the comparative CT method (ΔΔCT). The following primers were synthesized and used: Rat ferroptosis suppressor protein 1 (FSP1) qPCR primer pair was purchased from SinoBiological Company (Sinobiological, #RP300882); Human ferroptosis suppressor protein 1 qPCR Primer Pair purchased from Beyotime Company (Beyotime, #QH56669S); GAPDH qPCR primer pair was purchased from SinoBiological Company (Sinobiological, #RP300644, #HP100003). Human NAT10 qPCR primer pair was purchased from SinoBiological Company (Sinobiological, # HP103655). Rat NAT10 qPCR primer pair was designed by Deepseek and NCBI (Forward Primer: 5′-GGT TTC CTA TGG GCT GAC CC -3′; Reverse Primer: 5′-CGT CTT CGG AAA TCG GTC CA -3′). Human GPX4 qPCR primer pair was purchased from Beyotime Company (Beyotime, #QH15281S). Rat GPX4 qPCR primer pair was designed AI by Deepseek and NCBI (Forward Primer: 5′- ATG GAT GAA AGT CCA GCC CAA-3′; Reverse Primer: 5′-GTC CTT CTC TAT CAC CTG GGG -3′). Human GCLC qPCR Primer Pair was purchased from Beyotime Company (Beyotime, #QH14961S). Rat GCLC qPCR primer pair was designed AI by Deepseek and NCBI (Forward Primer: 5′- TCC ACT GTC CAA GGT TGA CG-3′; Reverse Primer: 5′-CTT GCT ACA CCC ATC CAC CA-3′). Human SLC7A11 qPCR Primer Pair was purchased from Beyotime Company (Beyotime, #QH14961S). Rat SLC7A11 qPCR primer pair was designed AI by Deepseek and NCBI (Forward Primer: 5′- TCG TCC TTT CAA GGT GCC TC -3′; Reverse Primer: 5′-TGA TAA GGA AGC CAA CCC CG -3′).

Statistical analysis

Data were analyzed using Graphpad Prism 8.01 software. Data were presented as mean ± standard deviation except were stated otherwise. All experiments that undergo error analysis were carried out in two or more independent measurements. Statistical analysis was performed

using unpaired two-tailed Student's t-test for two-group comparisons. When variances were not equal or the data were ranked, the Mann-Whitney test was applied. One-way ANOVA was applied to confirm the significant main effects and differences among three or more groups followed by Tukey's multiple comparisons for post hoc tests. For the grouped data, two-way ANOVA was used to analyze the levels of significant main effects followed by Bonferroni's multiple comparisons for post hoc tests. Comparison of survival curves was performed using the log-rank test (Mantel-Cox) test. For all experiments, * $p < 0.05$, ** $p < 0.01$, or *** $p < 0.001$ was considered statistically significant. Sample size: Explicit *n*-values provided for each experiment (e.g., *n* = 3 biological replicates) in the Figure legends.

References:

1. H. Su, S. Weng, L. Luo, *et al.* "Mycobacterium tuberculosis hijacks host macrophages-derived interleukin 16 to block phagolysosome maturation for enhancing intracellular growth," *Emerging Microbes & Infections* (2024): 2322663, https://doi.org/10.1080/22221751.2024.2322663
2. J. Wu, Z. Feng, L. Chen, et al., "TNF Antagonist Sensitizes Synovial Fibroblasts to Ferroptotic Cell Death in Collagen-Induced Arthritis Mouse Models," Nature Communications (2022): 676, https://doi.org/10.1038/s41467-021-27948-4
3. X. Peng, Y. Zhou, B. Zhang, *et al.*, "Mucosal recombinant BCG vaccine induces lung-resident memory macrophages and enhances trained immunity via mTORC2/HK1-mediated metabolic rewiring," *The Journal of Biological Chemistry* (2024):105518, https://doi.org/ 10.1016/j.jbc.2023.105518
4. R. W. Arts, B. Novakovic, R. Ter Horst, *et al.* "Glutaminolysis and Fumarate Accumulation Integrate Immunometabolic and Epigenetic Programs in Trained Immunity," *Cell Metabolism* (2016): 807-819, https://doi.org/10.1016/j.cmet.2016.10.008
5. H. Su, J. Huang, S. Weng, *et al*., "Glutathione synthesis primes monocytes metabolic and epigenetic pathway for β-glucan-trained immunity," *Redox Biology* (2021): 102206, https://doi.org/ 10.1016/j.redox.2021.102206
6. H. Su, Z. Liang, S. Weng, *et al.*, "miR-9-5p regulates immunometabolic and epigenetic pathways in β-glucan-trained immunity via IDH3α," *JCI Insight* (2021): e144260, 144260, https://doi.org/ 10.1172/jci.insight.144260
7. M. Li, S. Jin, Z. Zhang, *et al.*, "Interleukin-6 facilitates tumor progression by inducing ferroptosis resistance in head and neck squamous cell carcinoma," *Cancer Letters* (2022): 28-40, https://doi.org/10.1016/j.canlet.2021.12.011

2. Supplementary Figure legends

Supplementary Figure 1

Trained immunity in vitro and *in vivo*

(A) Schematic representation of *in vitro* trained immunity experimental setup. BMDMs were trained with 5.0 μg/mL β-glucan for 24 h. On day 6, cells were stimulated with medium or 1.0 ng/mL of LPS.

(B) IL-1β, IL-6 and TNFα production were analyzed in the supernatants of BMDMs response to LPS according to A (n = 5 to 6).

(C) The levels of glucose uptake, lactate production were examined in BMDMs response to LPS according to A (n = 6).

(D) The KDM5 activity was tested in BMDMs response to LPS according to A (n = 4).

(E) *In vivo* training rats model via β-glucan intravenous injection.

(F) Survival curves of rats trained with β-glucan followed with a lethal *C. albican* infection (n= 9 to 12).

In **B** to **D**, single dots correspond to individual mice, means ± SD are shown, $^{*}p < 0.05$ by unpaired Student's t-test; **F**, A pool of 2 experiments is shown, including 9 to 12 mice per group as indicated, $^{**}p < 0.01$ by log-rank test.

Supplementary Figure 2

Trained immunity affects inflammatory arthritis development in an established CIA rat model

(**A**) Images of the hind paw of rats, corresponding to **Figure 1 B** (n=3).

(**B** & **C**) Hematoxylin and eosin images of representative knee (**B**) and ankle (**C**) in rats at day 35, corresponding to **Figure 1 E**, scale bars, 200 μm.

Supplementary Figure 3

Adoptive transfer of trained macrophages aggravates inflammatory arthritis progression associated with phenotypic program of fibroblast-like synoviocytes

(**A**) Images of the hind paw of rats corresponding to **Figure 2 C** (n=3).

(**B** & **C**) Hematoxylin and eosin images of representative knee (**B)** and ankle (**C)** in rats at day 35, corresponding to **Figure 2 D**, scale bars, 200 μm.

(**D**) Immunofluorescence assays and the proportion of IL-β^{+}VCAM^{+} or IL-β^{+}/F4/80^{+} cells in the joint of rats, corresponding to **Figure 2 G**, scale bars, 50 μm.

Supplementary Figure 4

Trained immunity enhances proinflammatary response in FLSs

(**A**) Experimental setup of the co-culture model in a transwell chamber using PBS-Mφ or β-glu-Mφ and rat CIA-FLS. Macrophages and CIA-FLS were seeded at a ratio of 1:1 (approximately 1×10^5 cells), and were separated using a co-culture chamber for 72 h. Macrophages were placed in the upper chamber, and CIA-FLS were located in the lower chamber.

(**B**) The production of IL-6 and IL-10 was assessed by ELISA. Supernatant was collected after 72 h of co-culture incubation, according to **A** (n =4).

(**C**) RA-hFLS and β-glu-hMφ co-culture were seeded at a ratio of 1:1 (approximately 1×10^5 cells), and were separated using a co-culture chamber for 48 h. Macrophages were located in the upper chamber, and CIA-FLS were located in the lower chamber.

(**D**) Cytokines production analysis by ELISA according to **A** (n =3).

In **B**, **D**, means ± SD of are shown. $^{*}p < 0.05$, $^{***}p < 0.001$ by Ordinary one-way ANOVA, ns, none sense.

Supplementary Figure 5

Trained immunity reduces ferroptotic hallmarks in CIA Rat

(**A**) Immunohistochemical staining for 8-OHDG and 4-HNE in the inflamed joint tissue of CIA rat with adoptive transfer of PBS-Mφ or β-glu-Mφ, scale bars, 200 μm.

(**B**) Tile scan showed the whole scene of the fluorescent staining for VCAM and 4-HNE in the inflamed joint synovium of CIA rat with adoptive transfer of PBS-Mφ or β-glu-Mφ, scale bars, 50 μm.

(**C**) Tile scan showed the whole scene of the fluorescent staining of CIA rat joint synovium labelled with 13 anti-VCAM, anti-FSP1, and DAPI, scale bars, 50 μm.

Supplementary Figure 6

Trained immunity suppresses ferroptotic resolution for severity of arthritis in CIA Rats

(**A**) Three representative panoramic photographs of morphology in different groups, corresponding to **Figure 3C** (n = 3).

(**B**, **C**) Panoramic hematoxylin and eosin (H&E), toluidine blue O, and safranin O staining of representative joints in different groups, corresponding to **Figure 3 D**, Scale bars, 200 μm.

Supplementary Figure 7

IL-1β signal from the cross-talk between trained macrophages and CIA-FLS empowered CIA-FLS a ferroptosis-resistant phenotype

(A) CIA-FLS and PBS-Mφ or β-glu-Mφ were seeded at a density of 1 × 10^5 cells/well on the surface of a transwell culture plate at a ratio of 1:1, and were separately co-cultured for 72 h, CIA-FLS then was treated with iFPS1 (2.0 µM), Erastin (5.0 µM), or RSL3 (0.5 µM). Cell viability of CIA-FLS was assessed by measuring CCK-8 levels at 18 h (iFPS1), 24 h (Erastin), or 12 h (RSL3) (n=5).

(**B**) CIA-FLS with GPX4 KD were co-cultured with trained macrophages in the presence or absence of 100.0 µM Trolox for 72 h, cell viability of CIA-FLS were analyzed (n = 4).

(**C**) CIA-FLS and PBS-Mφ or β-glu-Mφ were co-cultured in the presence of anti-IL-1R (3.5 µg/mL) for 72 h, cell viability of CIA-FLS was assessed at 18 h (2.0 µM iFPS1) (n=4).

(**D**) In the presence of the blocking antibodies to anti-IL-6R (3.5 µg/mL), anti-gp130 (3.5 µg/mL), anti-TNFR1 (3.5 µg/mL), or anti-TNFR2 (4.5 µg/mL) for 72 h, followed by iFPS1 (2.0 µM), cell viability was assessed at 18 h (n=5 to 6).

(E) q-PCR analysis of FSP1, NAT10, GPX4, or GCLC mRNA as described in **A** (n=3).

In **A** to **E**, means ± SD are shown, **A**, **B**, $^{*}p < 0.05$, $^{**}p < 0.01$, $^{***}p < 0.01$ by Ordinary one-way ANOVA; **C** to **E**, $^{*}p < 0.05$, $^{**}p < 0.01$, $^{***}p < 0.01$ by Two-way ANOVA, ns, none sense.

Supplementary Figure 8

IL-1β signal protects inflammatory arthritis synovial fibroblasts from ferroptosis via promoting NAT10-mediated N4-acetylation on FSP1 mRNA

(**A**) Primed with IL1-β (10.0 ng/ml) or TNFα (5.0 ng/ml) for 72 h, viability of CIA-FLS (1 × 10^5 cells/well) was examined at 18 h (iFPS1, 0 to 2.0 µM) (n=5).

(**B**) After primed, lipid peroxidation (MDA) were measured in CIA-FLS (1 × 10^6 cells/well) after treatment with iFPS1 (1.0 µM) ± ferrostatin-1 (Fer1) (1.5 µM) for 4 h (n=4).

(**C**) With anti-IL-1β (4.5 µg/mL) ± IL-1β (10.0 ng/mL) for 72 h, CIA-FLS viability was assessed at 18 h (iFPS1, 2.0 µM) (n= 3 to 4).

(**D**) FSP1, NAT10, GPX4 or Actin protein levels in CIA-FLS after treated with anti-IL-1β (4.5 µg/mL) ± IL-1β (10.0 ng/mL) for 72 h (n=2).

(**E**) q-PCR analysis of FSP1, NAT10, GPX4, or GCLC mRNA as described in **D** (n=4).

(**F**) N4-acetylation on FSP1 mRNA in CIA- FLS with anti-IL-1β (4.5 µg/mL) ± IL-1β (10.0 ng/mL) for 72 h (n=3 to 4).

(**G**) After with IL1-β (10.0 ng/ml) ± Remodelin (25.0 µM) for 72 h, cell viability of CIA- FLS was tested in the presence of 2.0 µM iFPS1 at 18 h (n=3).

(**H**) Schematic diagram.

In **A** to **C**, **E** to **G**, means ± SD are shown, **A**, $^{*}p < 0.05$, by Two-way ANOVA; B, **C**, **E**, **F**, G, $^{**}p < 0.01$, $^{***}p < 0.001$ by One-way ANOVA, ns, none sense.

Supplementary Figure 9

Trained macrophages drives NAT10-mediated ac4C modification on FSP1 mRNA and ferroptotic resistance in CIA-FLS

(**A**) Immunohistochemical staining of NAT10 in the inflamed joint tissue of CIA rat, scale bars, 200 μm.

(**B**) Immunofluorescence assays of $NAT10^{+}VCAM^{+}$ cells in the joints of CIA rat with adoptive transfer of PBS-Mφ or β-glu-Mφ, scale bars, 50 μm.

(**C**) CIA-FLS transfected with sh-NAT10 or sh-Ctrl were co-cultured with β-glu-Mφ at a ratio of 1:1 for 72 h, level of NAT10 mRNA in CIA-FLS was analyzed (n=3).

(**D**) CIA-FLS transfected with sh-NAT10 or sh-Ctrl were co-cultured with PBS-Mφ or β-glu-Mφ at a ratio of 1:1 for 72 h, levels of ac4C FSP1 mRNA in CIA-FLS was examined (n = 3 to 4).

(**E**) Western blot analysis of FSP1, NAT10 or Actin expression, according to **D.**

(**F**) CIA-FLS transfected with sh-NAT10 or sh-Ctrl were co-cultured with PBS-Mφ or β-glu-Mφ at a ratio of 1:1 for 72 h, cell viability of CIA-FLS was examined After treatment with 2.0 μM iFPS1 at 18 h (n = 3).

In **C**, **D**, **F**, means ± SD are shown, $^{***}p < 0.001$ by paired Student's t-test (**C**, **D**); $^{***}p < 0.001$ **by** Two-way ANOVA (**F**).

Supplementary Figure 10

Therapeutic targeting of trained immunity, combination of Remodlin with ferroptosis inducer alleviated the RA symptom aggravated by trained immunity

(**A** to **D**) Panoramic hematoxylin and eosin (H&E) (**A**), toluidine blue O (**B**), and safranin O (**C**) staining of representative joints in different groups, Scale bars, 200 μm. (**D**) $^{*}p < 0.05$, by One-way ANOVA/Tukey’s multiple comparisons test, corresponding to **Figure 5 D**.

Supplementary Figure 11

Therapeutic targeting of trained immunity, combination of Remodlin with ferroptosis inducer triggered synovial fibroblast ferroptosis

(**A**) Tile scan showed the whole scene of the fluorescent staining for VCAM and 4-HNE in the

inflamed joint synovium of rats according to **Figure 5G**, scale bars, 50 μm.

(**B**) Tile scan showed the whole scene of the fluorescent staining of rats joint synovium labelled with anti-VCAM, anti-FSP1, and DAPI according to **Figure 5G**, scale bars, 50 μm.

Supplementary Figure 12

CAI-induced maladaptive inflammation enhances macrophages training

(**A**) IL-β production in BMDMs from CIA rat in response to 1 ng/mL LPS restimulation in vitro corresponding to **Figure 6A** (n= 4 to 6).

(**B**) Schematic representation of the metabolic programming during trained immunity.

(**C**) The levels of lactate, fumarate, glutamine, or α-KG in BMDM from different groups of CIA rat, corresponding to **Figure 6A** (n=4 to 6).

(**D**) The relative activity of histone methyltransferase in BMDMs from different groups of CIA rat, corresponding to **Figure 6A** (n= 3 to 4).

In **A**, **C**, **D**, means ± SD are shown, $^{*}p < 0.05$ by One-way ANOVA (A, **C**, **D**).

Supplementary Figure 13

CAI-induced maladaptive inflammation enhances macrophages training, contributing to aggravated severity of arthritis

(**A**) Panoramic photographs of morphology in different groups, corresponding to **Figure 6 D**, Scale bars, 200 μm.

(**B**, **C**) Panoramic H&E staining of representative knee (**B**) and ankle (**C**) in different groups, corresponding to **Figure 6E**, Scale bars, 200 μm.

(**D**, **E**) Panoramic representative immunofluorescence assays of IL-β^{+}VCAM^{+} or IL-β^{+}/F4/80^{+} cells in the joints of rats corresponding to **Figure 6G**, Scale bars, 50 μm.

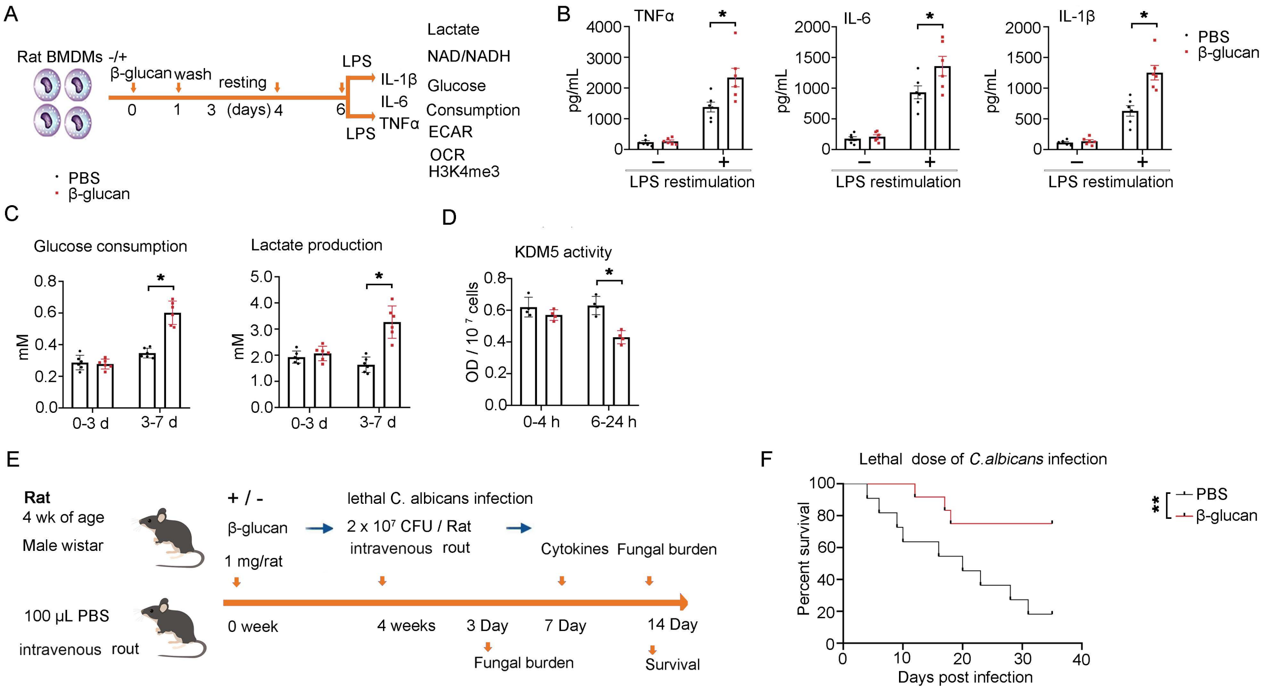

Supplementary Figure 1

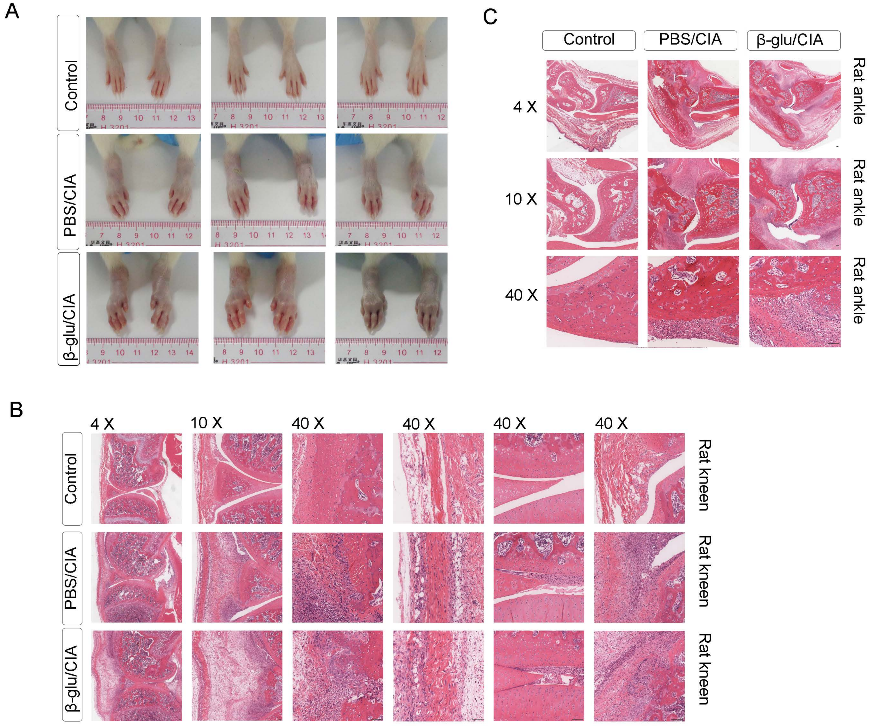

Supplementary Figure 2

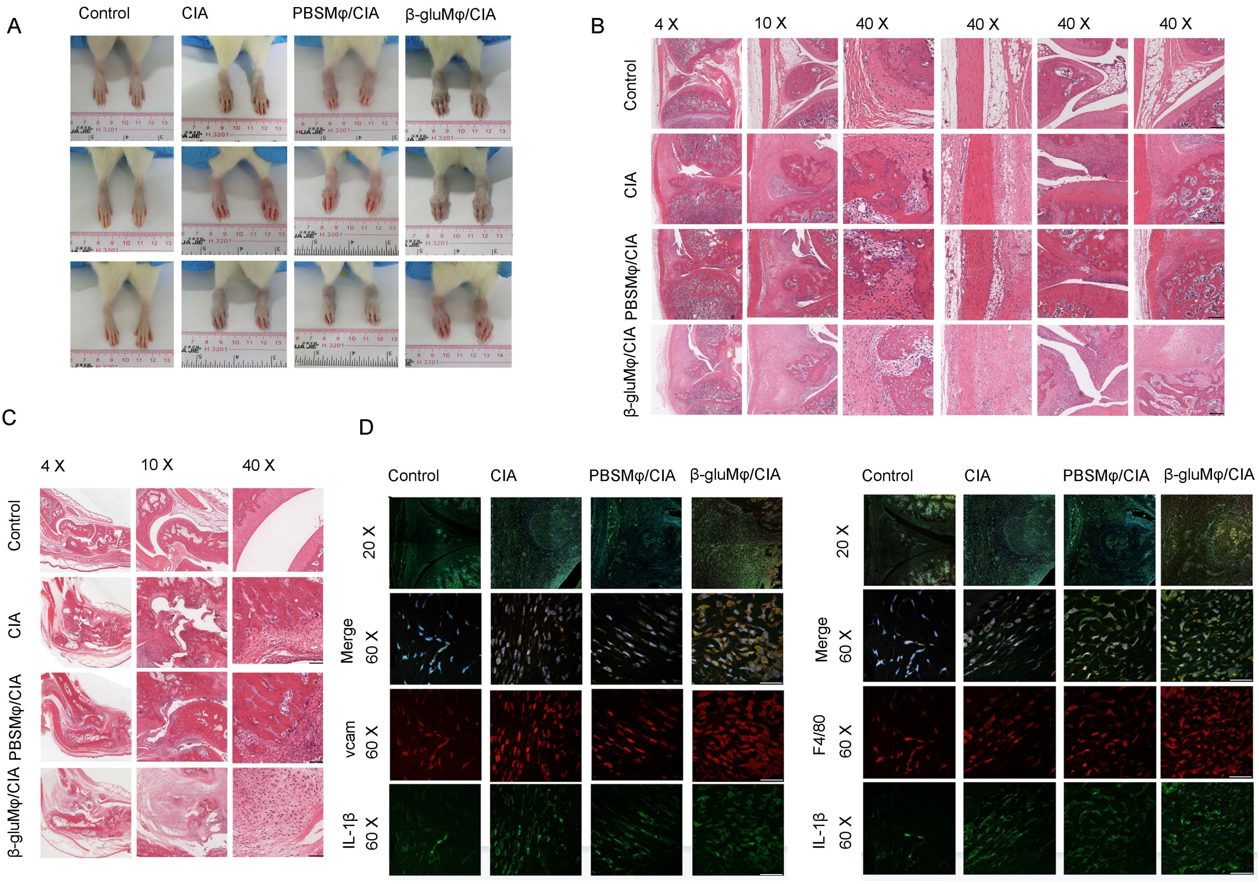

Supplementary Figure 3

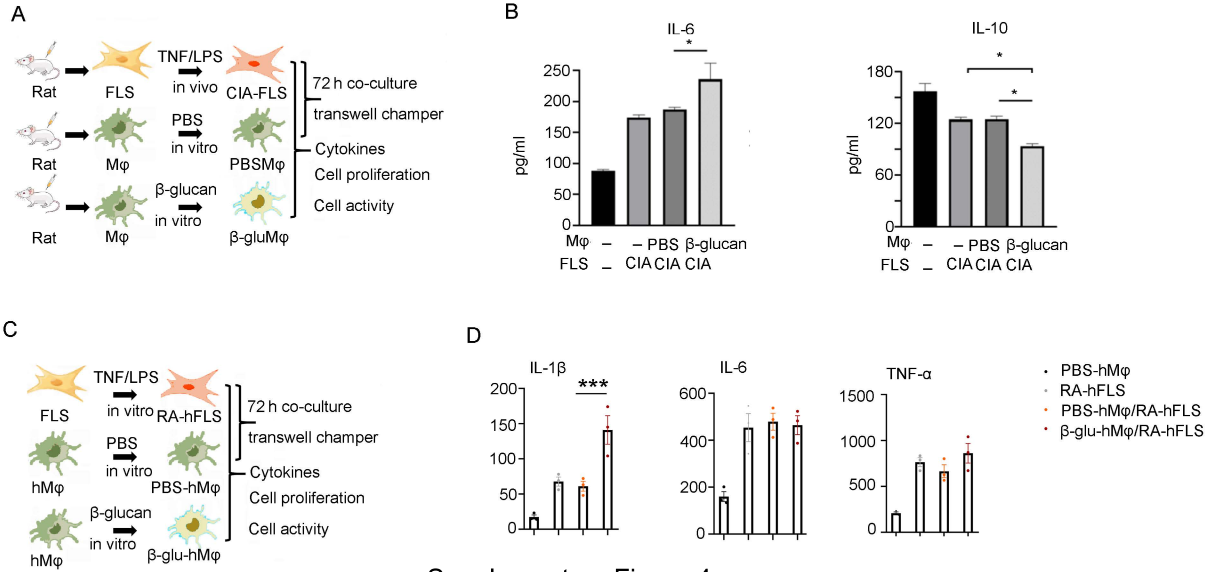

Supplementary Figure 4

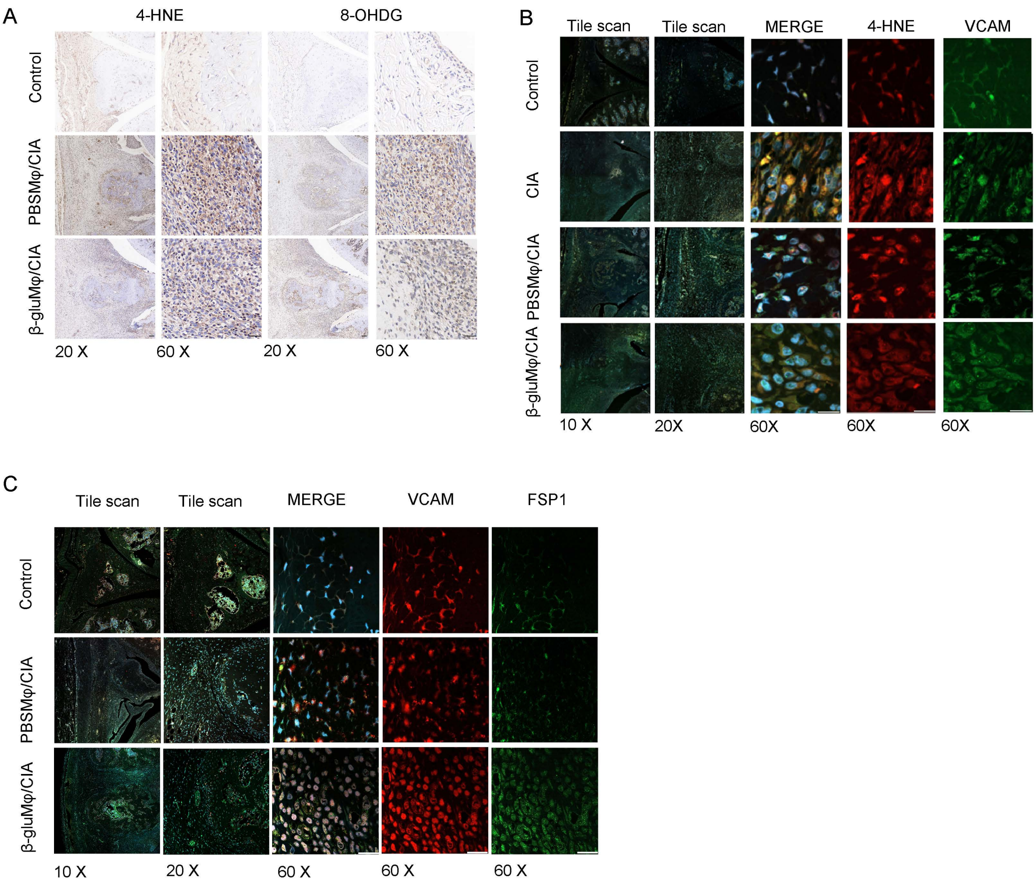

Supplementary Figure 5

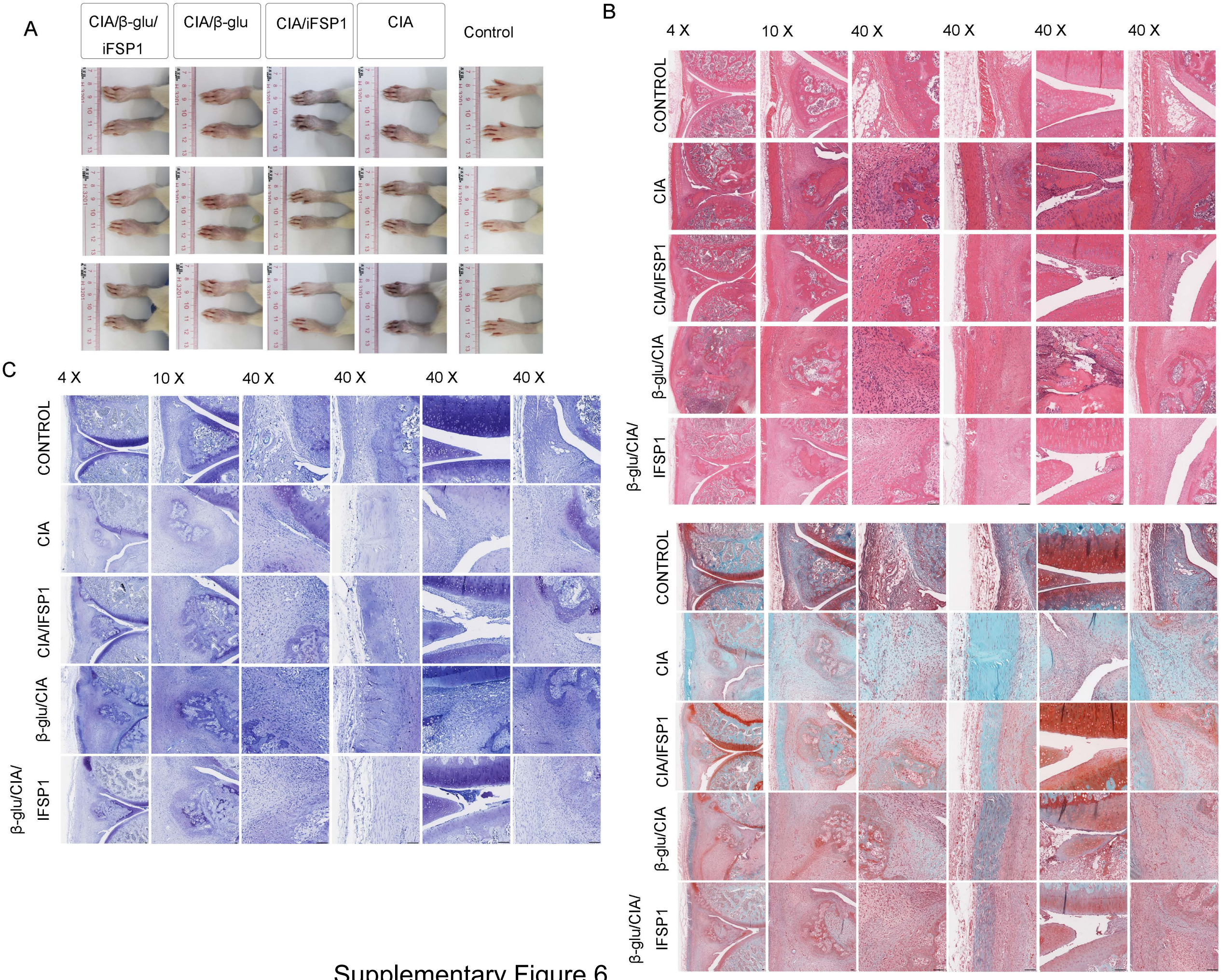

Supplementary Figure 6

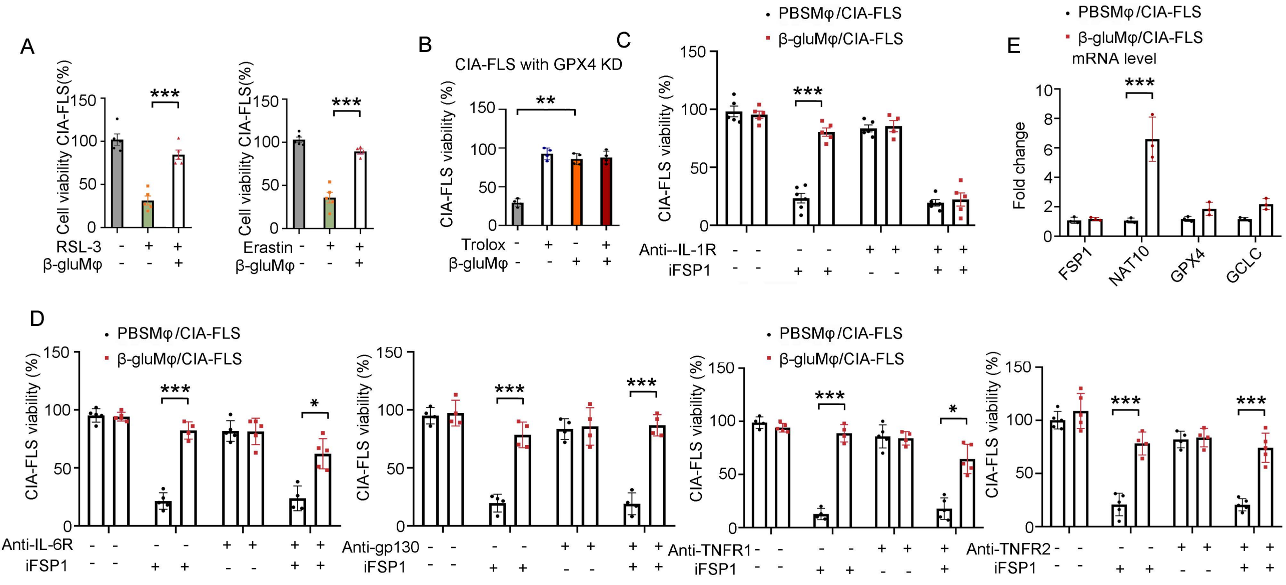

Supplementary Figure 7

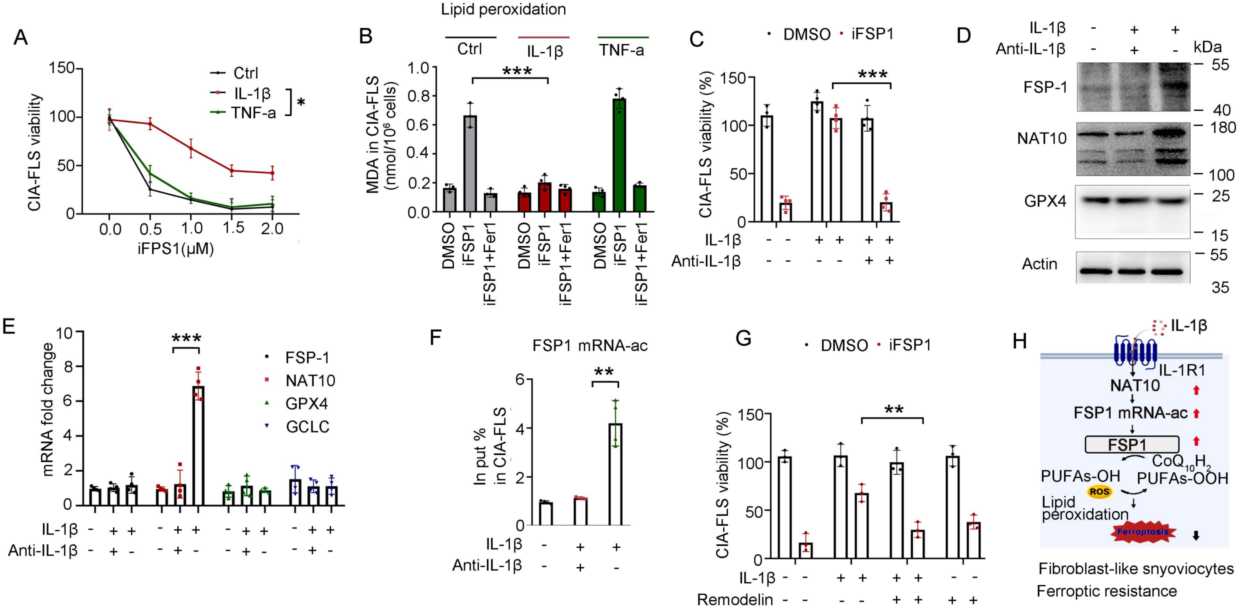

Supplementary Figure 8

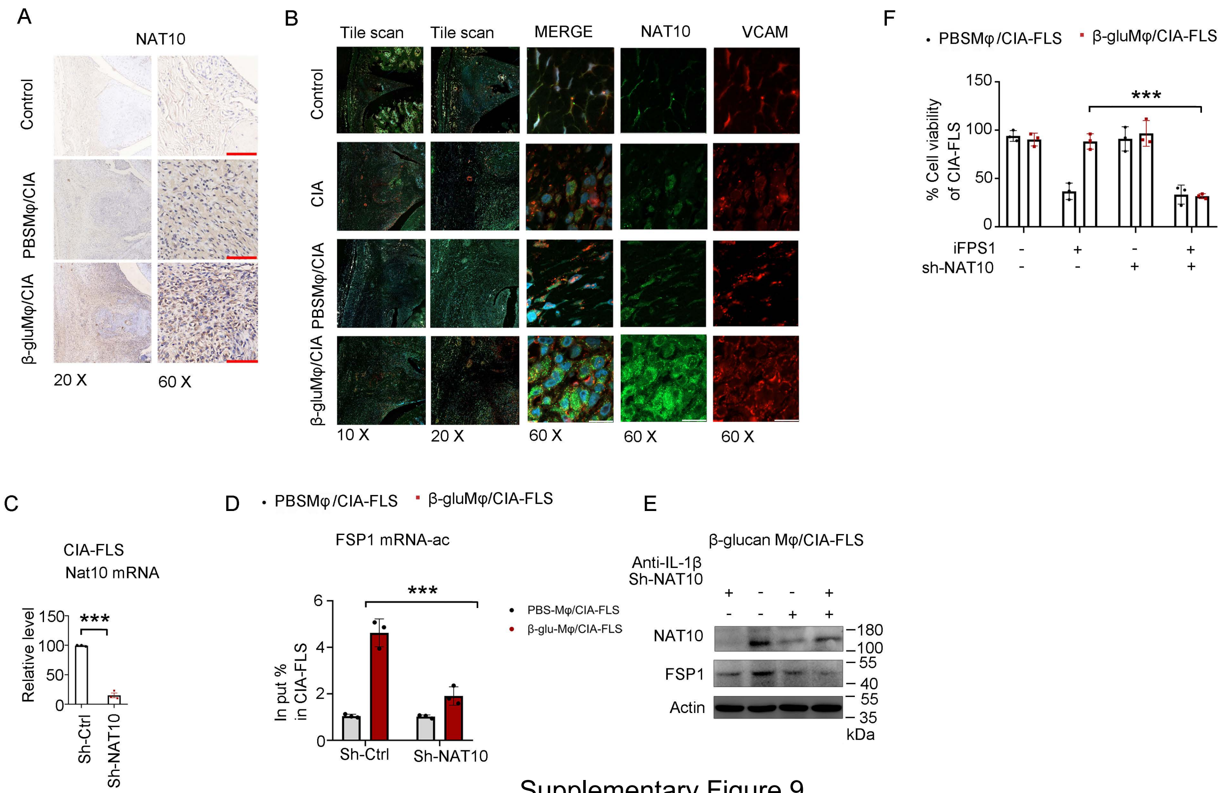

Supplementary Figure 9

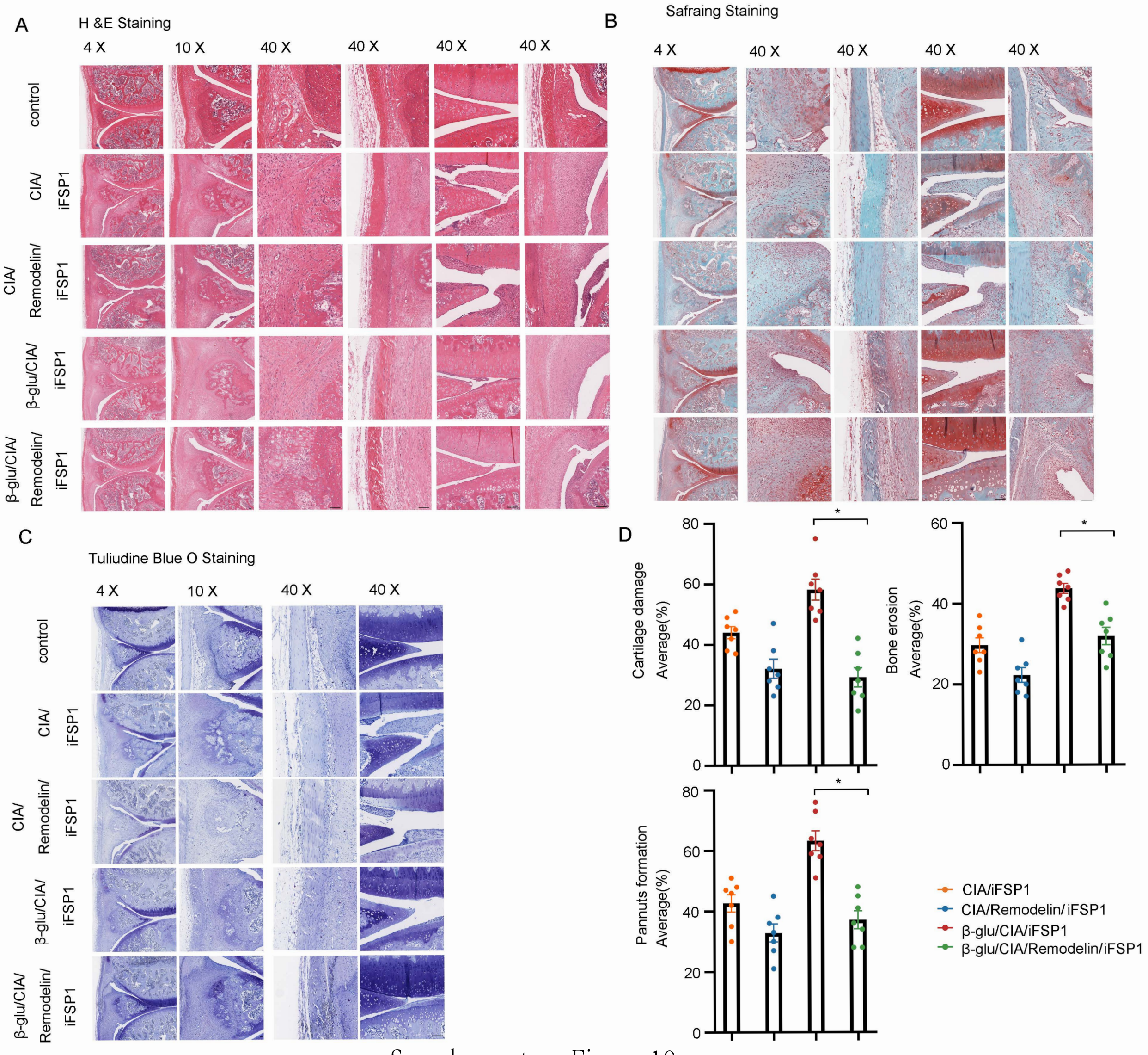

Supplementary Figure 10

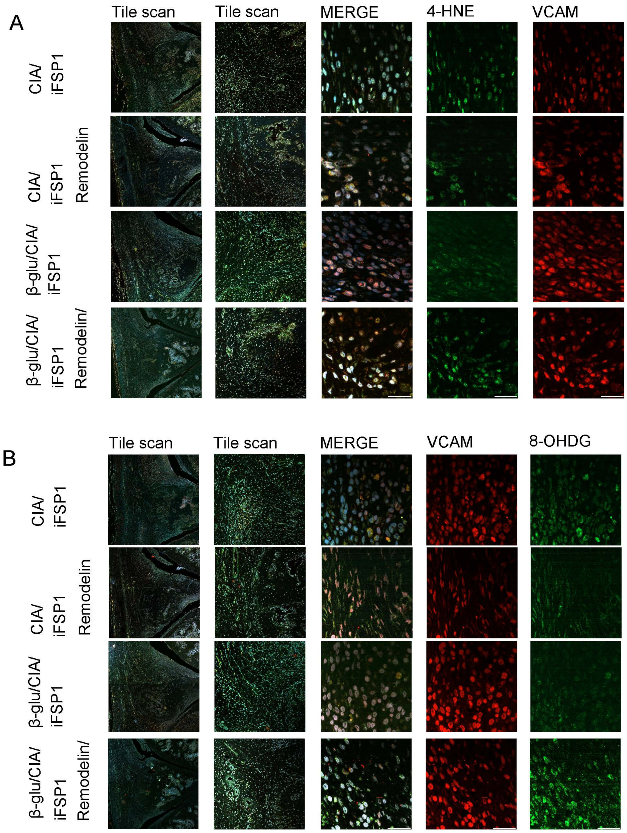

Supplementary Figure 11

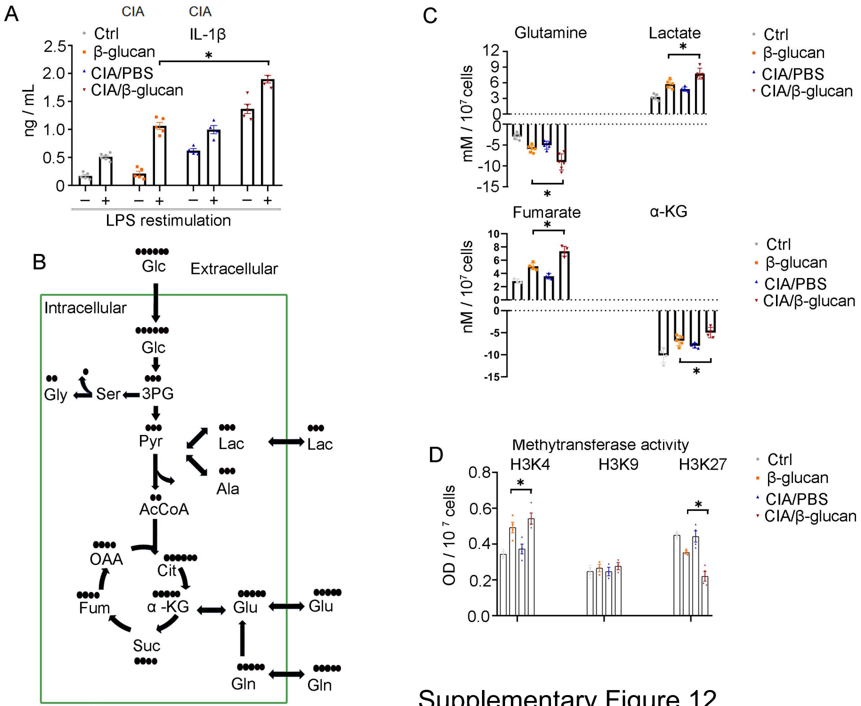

Supplementary Figure 12

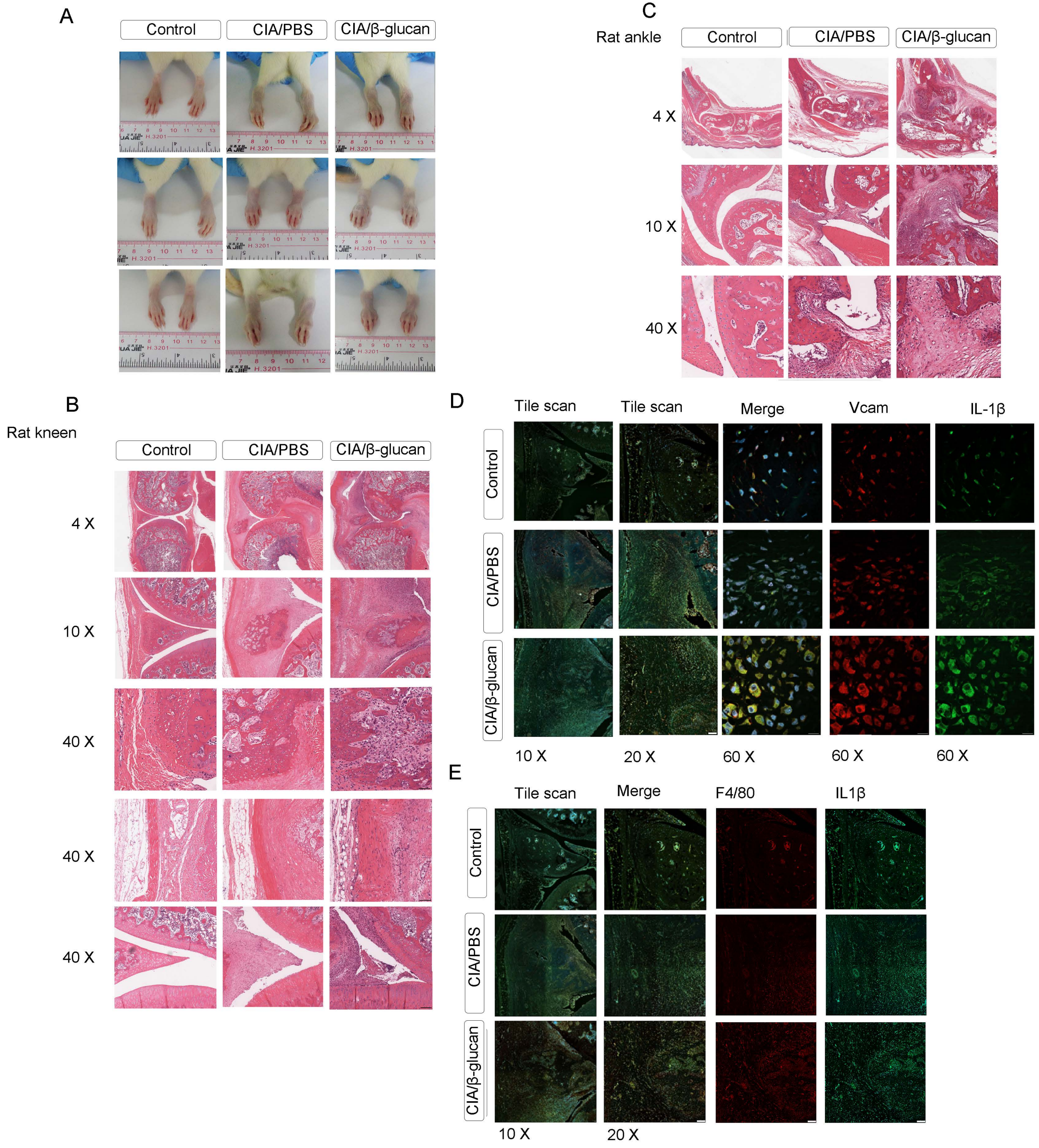

Supplementary Figure 13

广州医科大学实验动物福利伦理委员会审查报告

Committee review of animal experiments in Guangzhou Medical University

实验名称<br>(Title)	类风湿关节炎在卡介苗介导的训练免疫中调控甲羟戊酸代谢和 H3K4me3 表观遗传重编程的机制研究<br>（Mechanisms regulating mevalonate metabolism and H3K4me3 epigenetic reprogramming in BCG-mediated training immunity in rheumatoid arthritis）		
项目来源<br>(Project sources)	广医-广州生物院联合生科院<br>（Guangzhou Medical-Guangzhou Biological Institute Joint Academy of Biological Sciences）		
项目申请人<br>(Applicant)	粟海波<br>Su Haibo	受理编号<br>(Acceptance number)	GY2023-259
审查形式<br>(Auditing)	函审<br>Letter correspondence	审查时间<br>(Processing time)	2023-07-05

审查结果(Results of the review)：

该实验方案符合动物福利伦理要求，通过动物实验伦理审查，准予开展动物实验。

According to the rules of Committee on Animal Research and Ethics, this research project has been reviewed and approved to be appropriate and humane by institutional animal care and use committee.

广州医科大学实验动物福利伦理委员会

Institutional Animal Care and Use Committee of Guangzhou Medical University

签章(Signature)：

时间(time)： 2023-07-03

Ethic approval

Supplementary Figure 8D

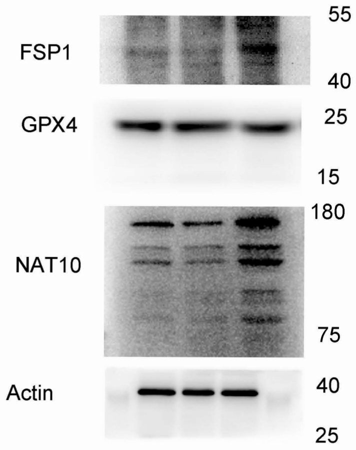

Supplementary Figure 9E

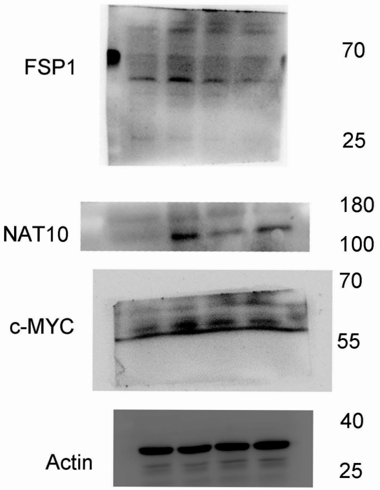

Figure 4F

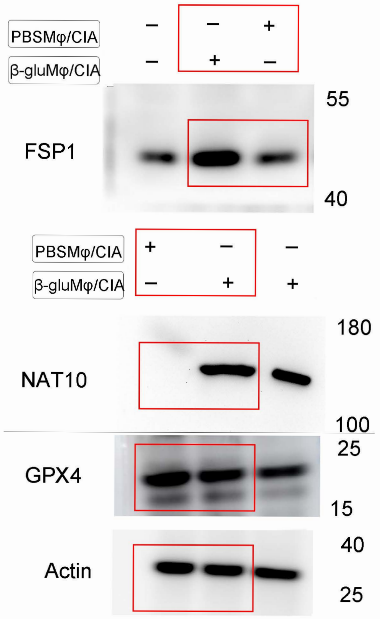

Figure 4I

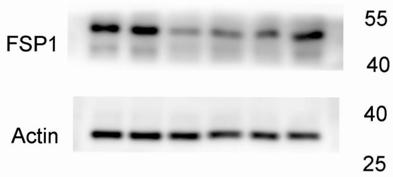

Raw data-Western blots

Fig1-B

Raw data-Paws microscope images for Figure 1B

CON

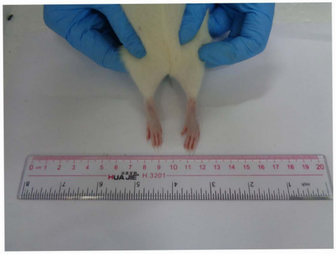

PBS/CIA

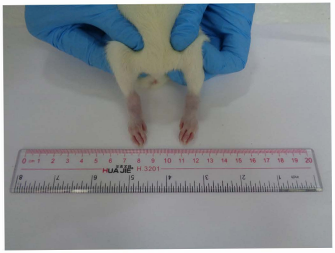

β-glu/CIA

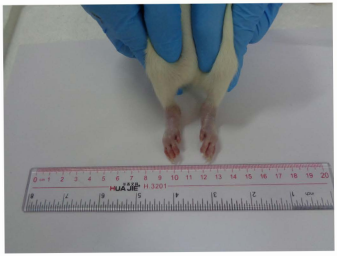

Fig2-C

Raw data-Paws microscope images for Figure 2C

CON

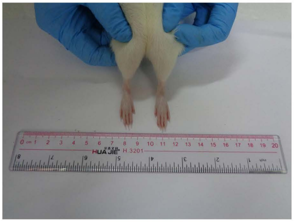

CIA

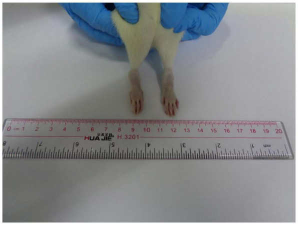

PBS M/CIA

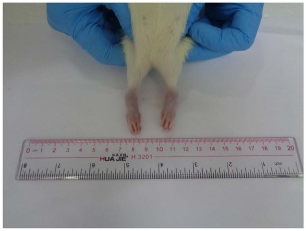

β-glu M/CIA

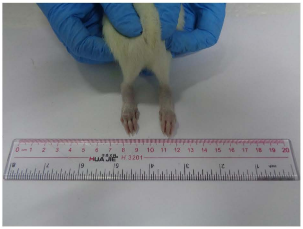

Fig3-C

Raw data-Paws microscope images for Figure 3C

CON

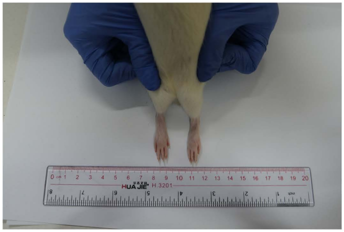

CIA

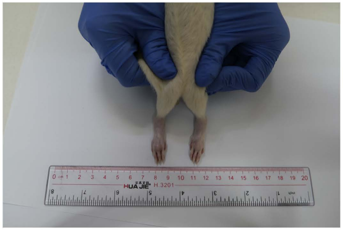

CIA/iFSP1

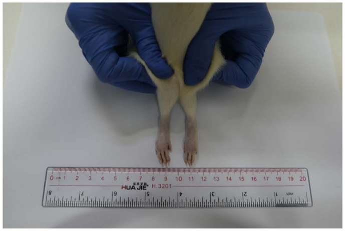

CIA/ β -glu

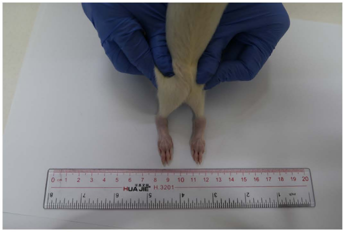

CIA/ β -glu/iFSP1

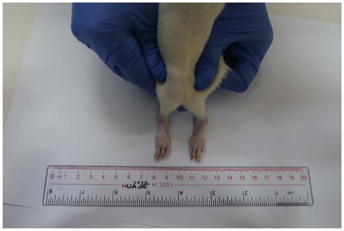

Fig5-C

Raw data-Paws microscope images for Figure 5C

CON

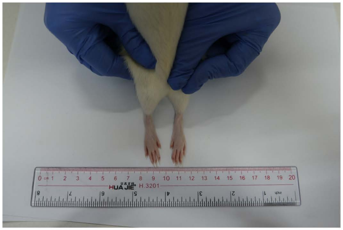

CIA/iFSP1

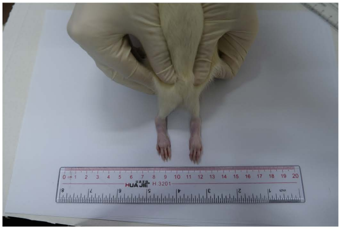

β-glu/CIA/
iFSP1

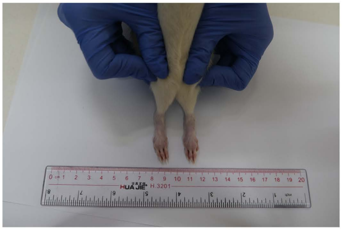

CIA/Remodelin/
iFSP1

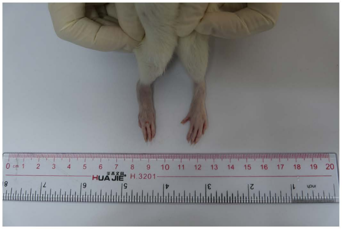

β-glu/CIA/
Remodelin/iFSP1

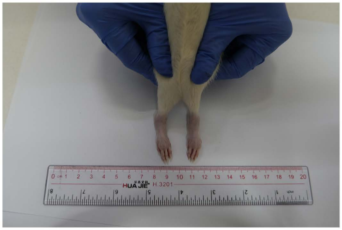

Fig6-D

Raw data-Paws microscope images for Figure 6D

CON

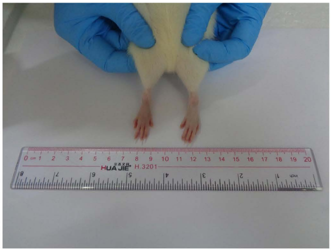

CIA/PBS

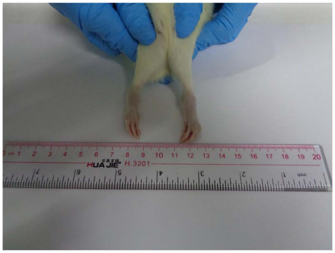

CIA/ β -glu

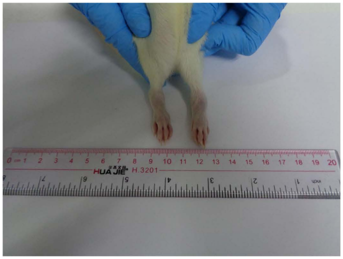

S-Fig2-1 A

Raw data-Paws microscope images for Supplementary Figure 2A

CON

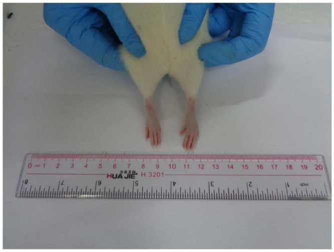
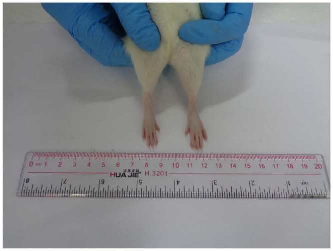
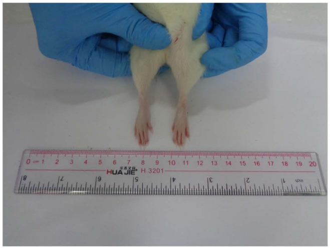

PBS/CIA

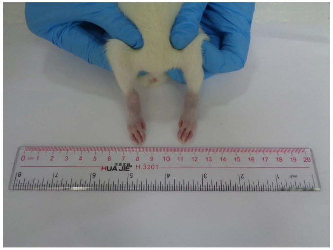
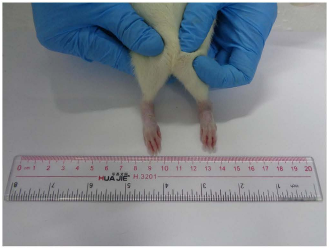
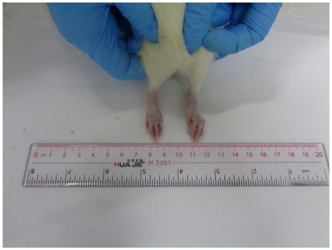

β-glu/CIA

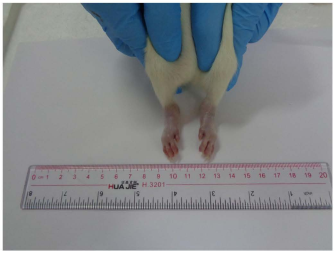
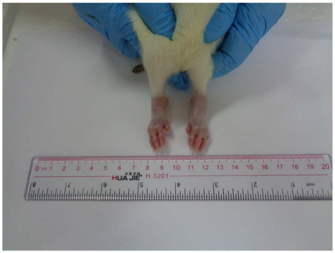
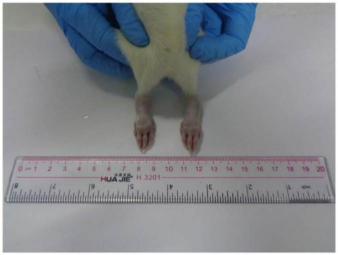

S-Fig3-1A

Raw data-Paws microscope images for Supplementary Figure 3A

CON	CIA	PBS M/CIA	β-glu M/CIA

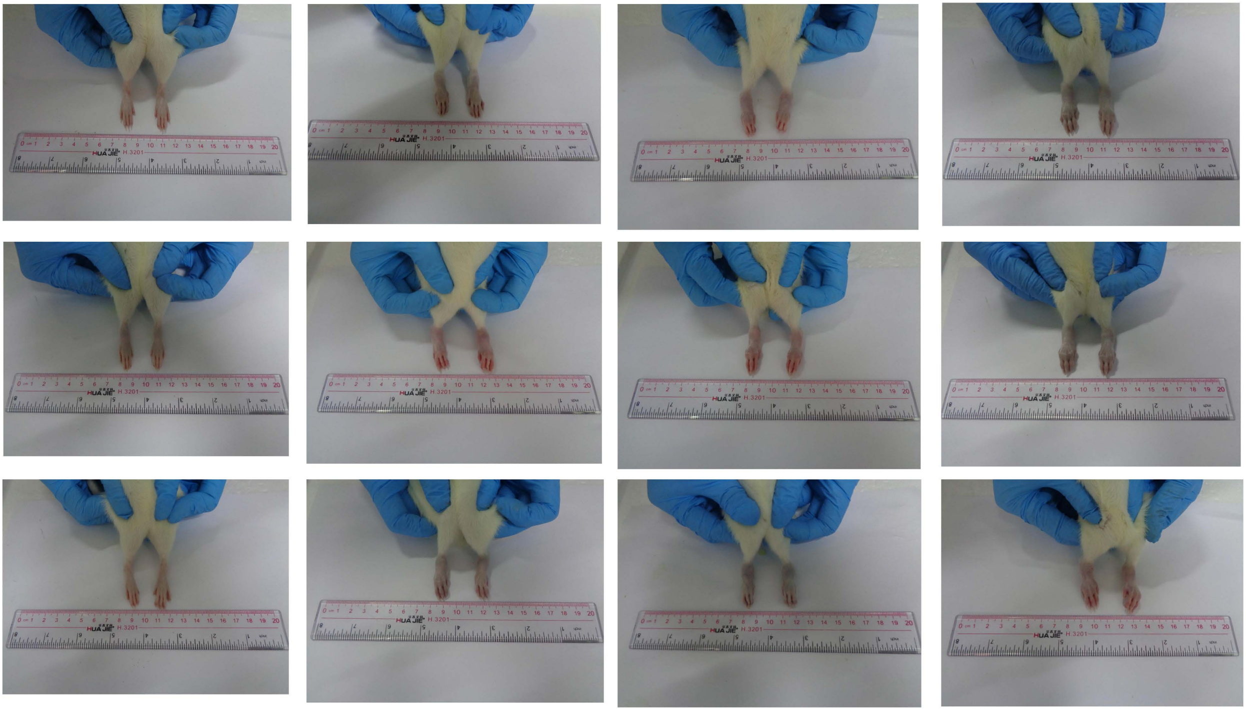

S-Fig6-1A

Raw data-Paws microscope images for Supplementary Figure 6A

CON

CIA

CIA/iFSP1

CIA/ β -glu

CIA/ β -glu/iFSP1

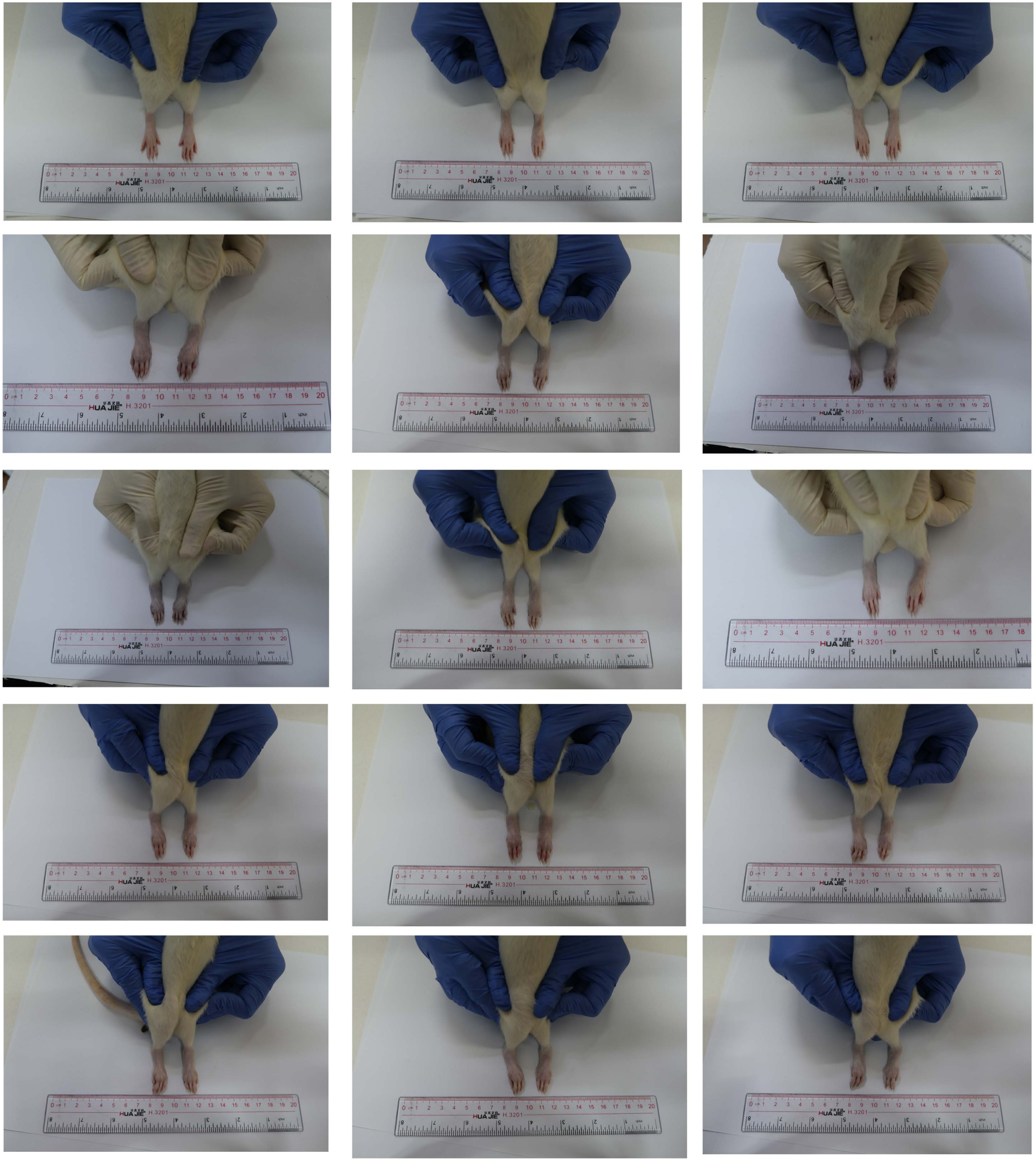

S-Fig13-1A

Raw data-Paws microscope images for Supplementary Figure 13A

CON | CIA/PBS | CIA/β-glu

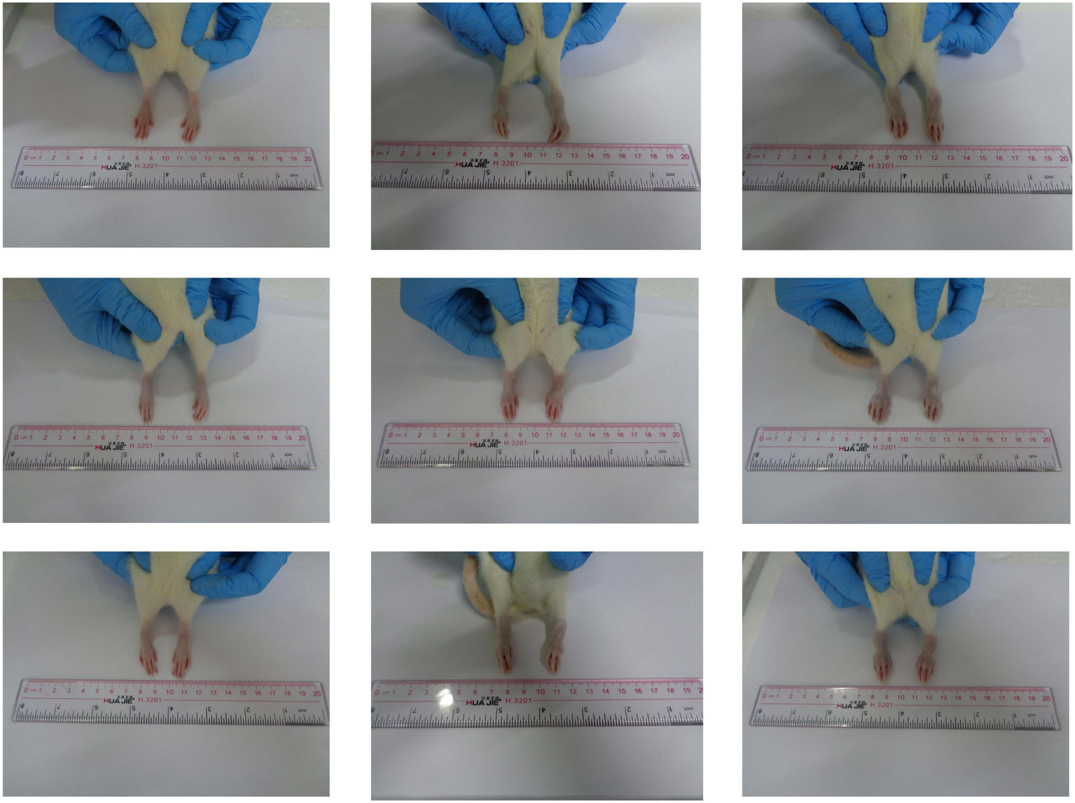

Raw data-H&E microscope images for Figure 1E

Fig1-E

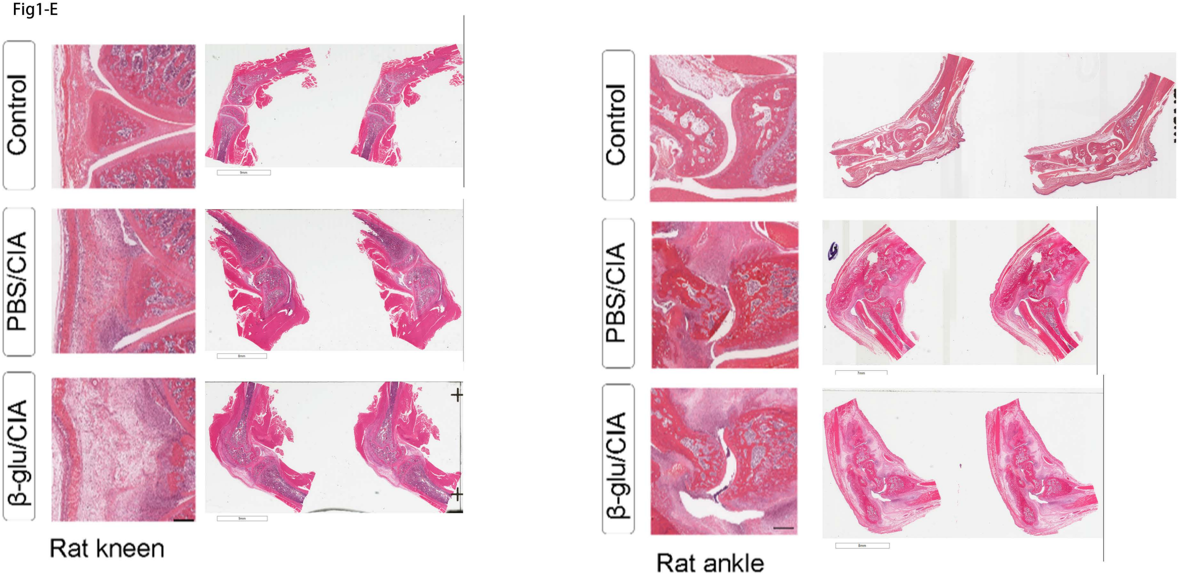

Fig2-D

Raw data-H&E microscope images for Figure 2D

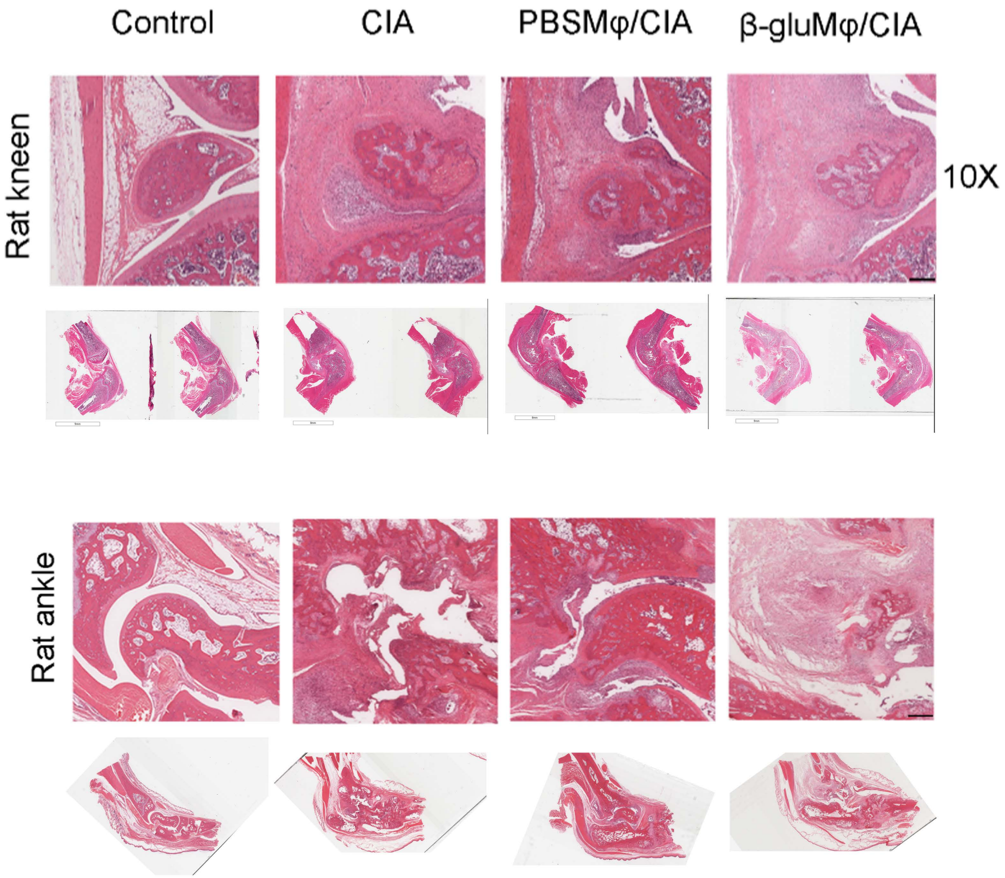

Fig3-D

Raw data-H&E microscope images for Figure 3D

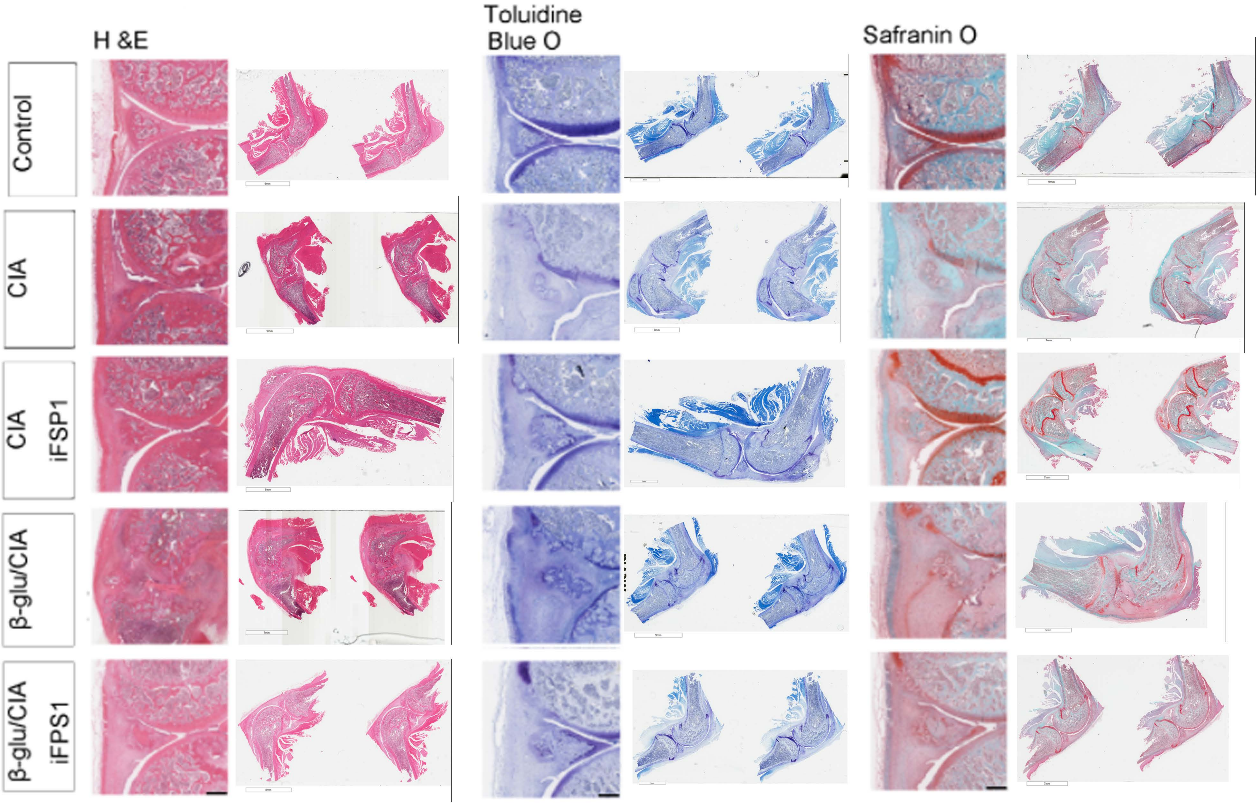

Fig5-D

Raw data-H&E microscope images for Figure 5D

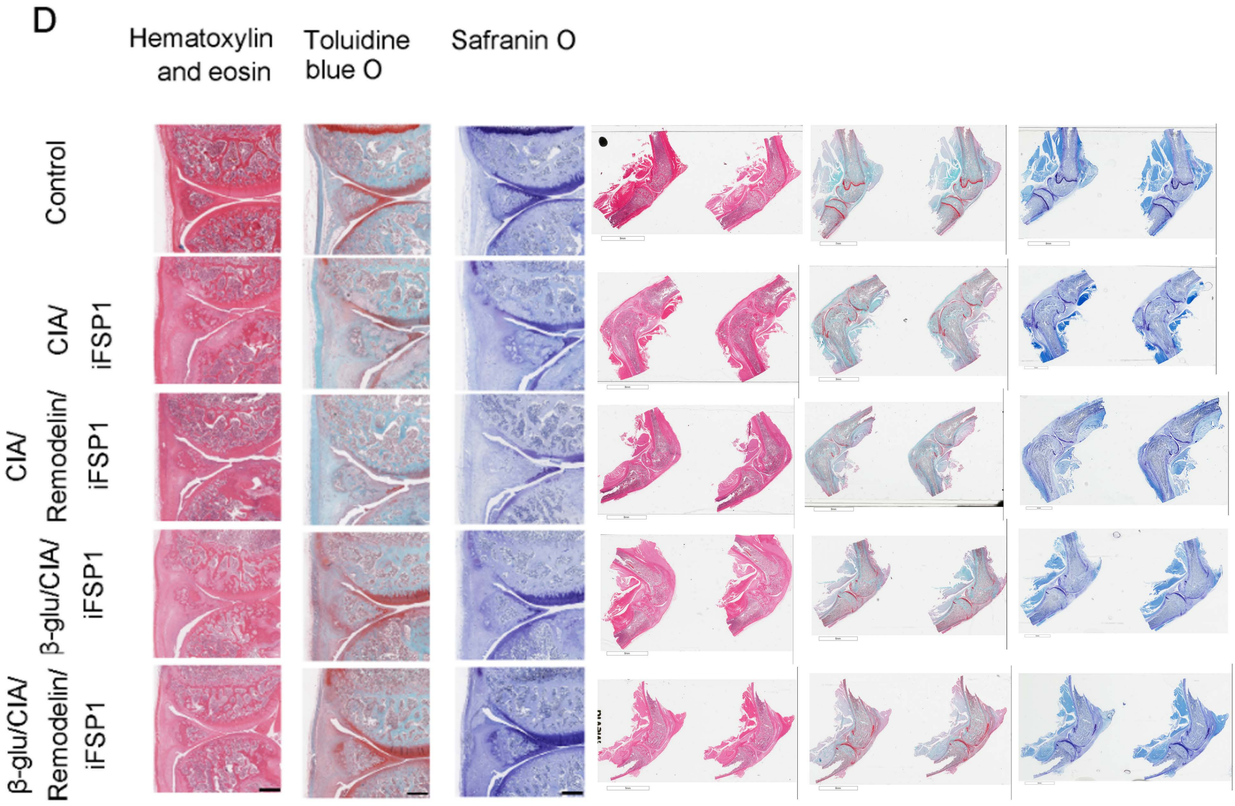

Fig6-E

Raw data-H&E microscope images for Figure 6E

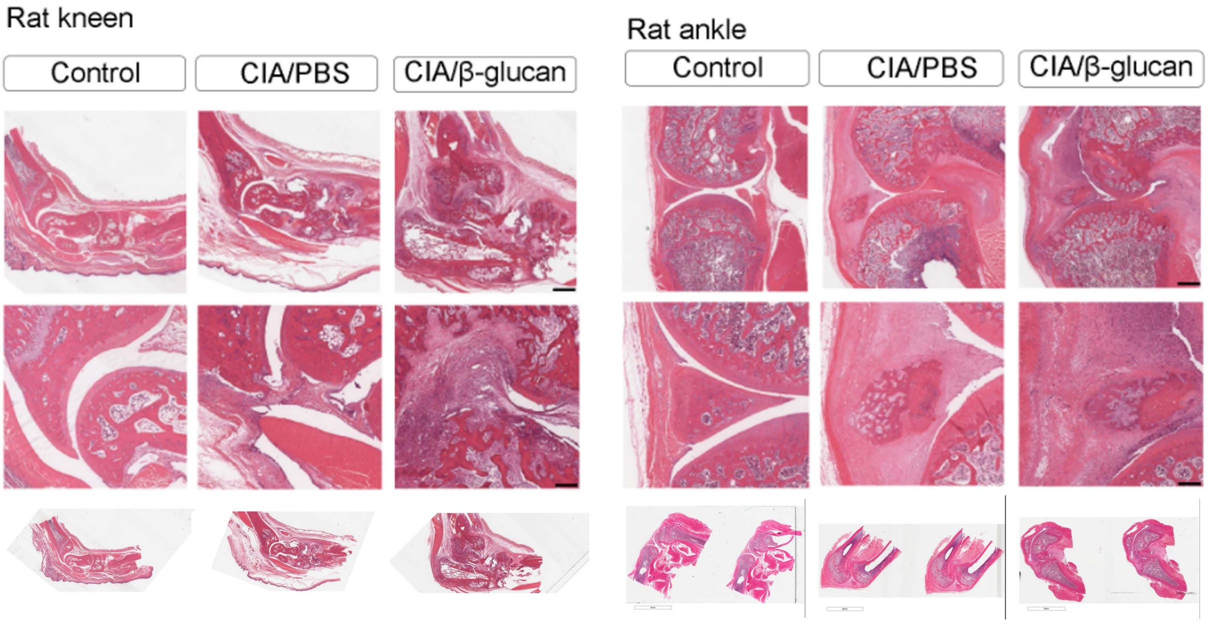

S-Fig2-1B

Raw data-H&E microscope images for Supplementary Figure 2B

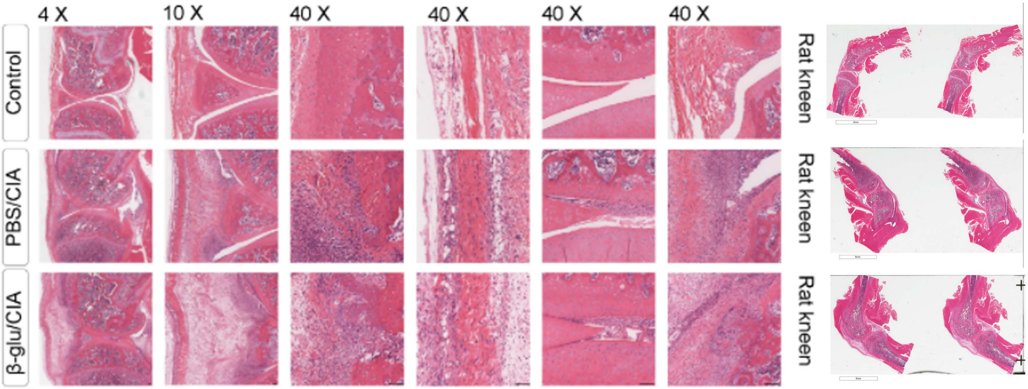

S-Fig2-1C

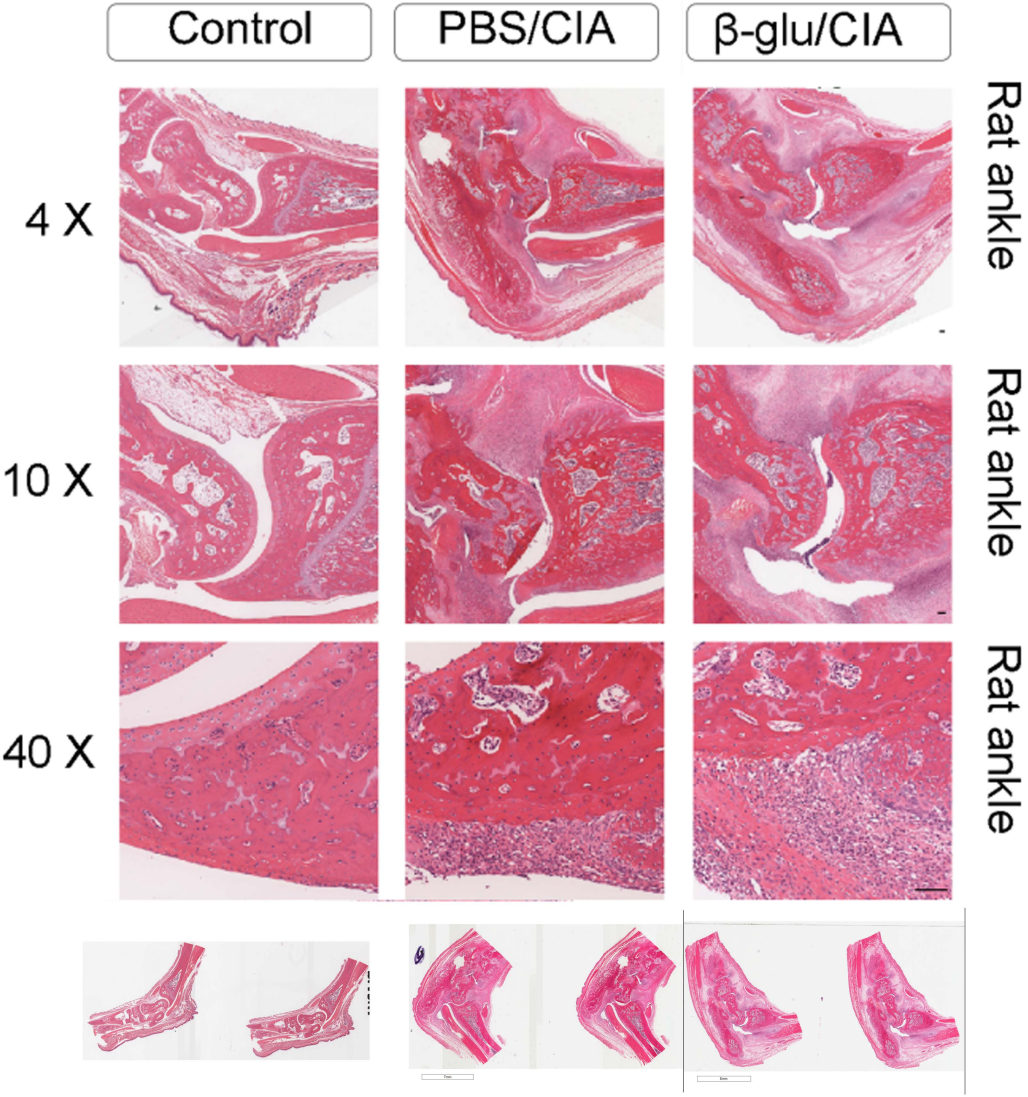

Raw data-H&E microscope images for Supplementary Figure 2C

S-Fig3-1B

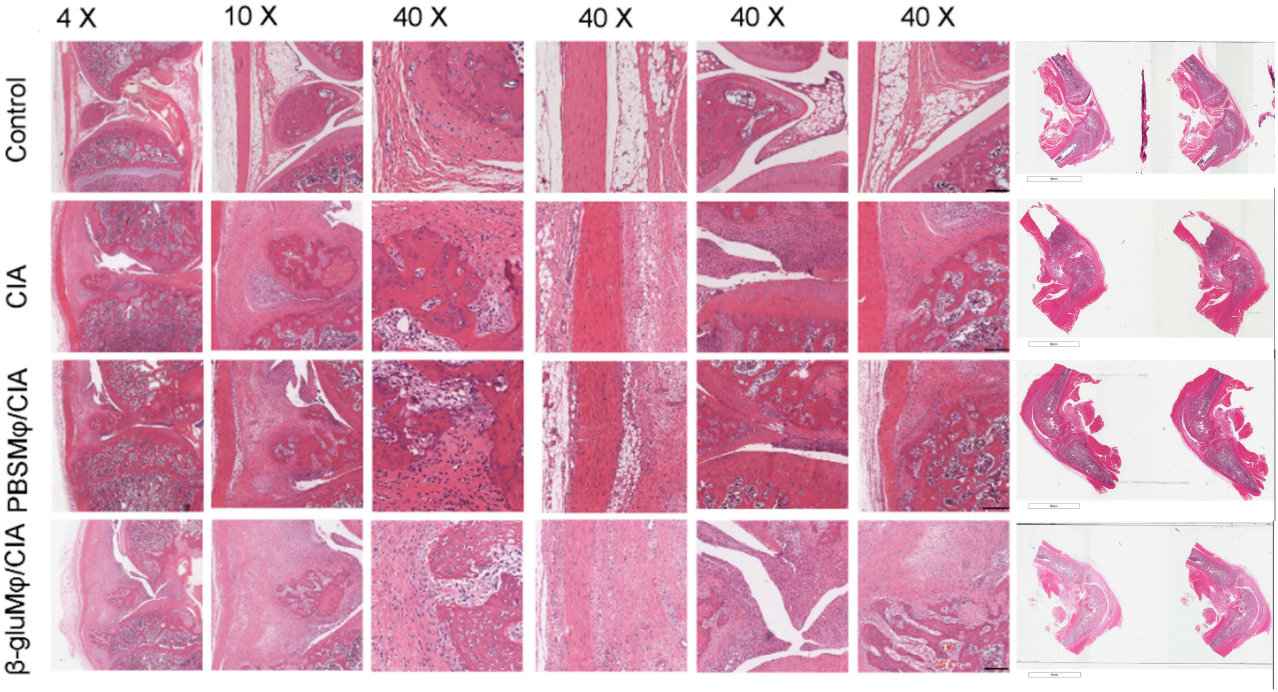

Raw data-H&E microscope images for Supplementary Figure 3B

S-Fig3-1C

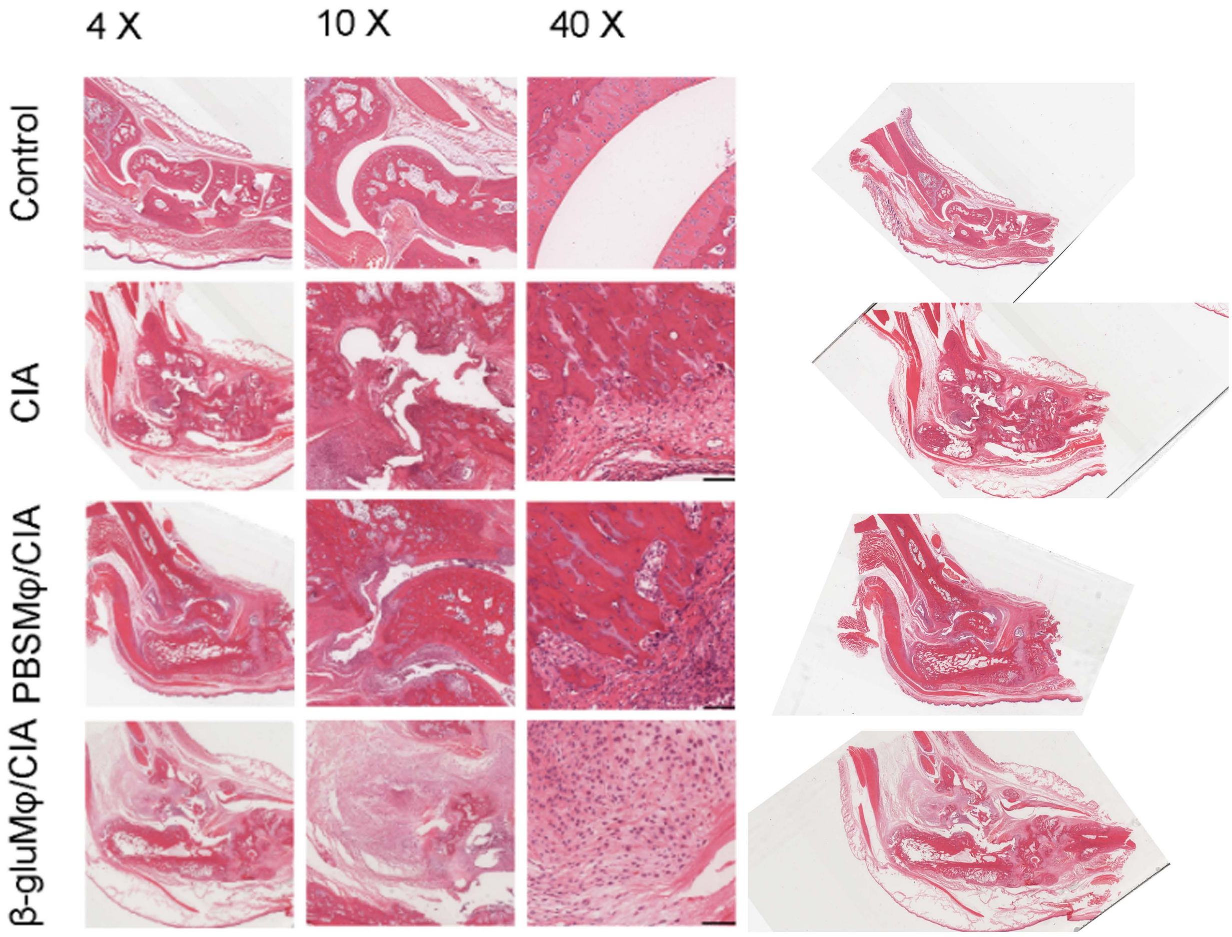

Raw data-H&E microscope images for Supplementary Figure 3C

S-Fig6-1B

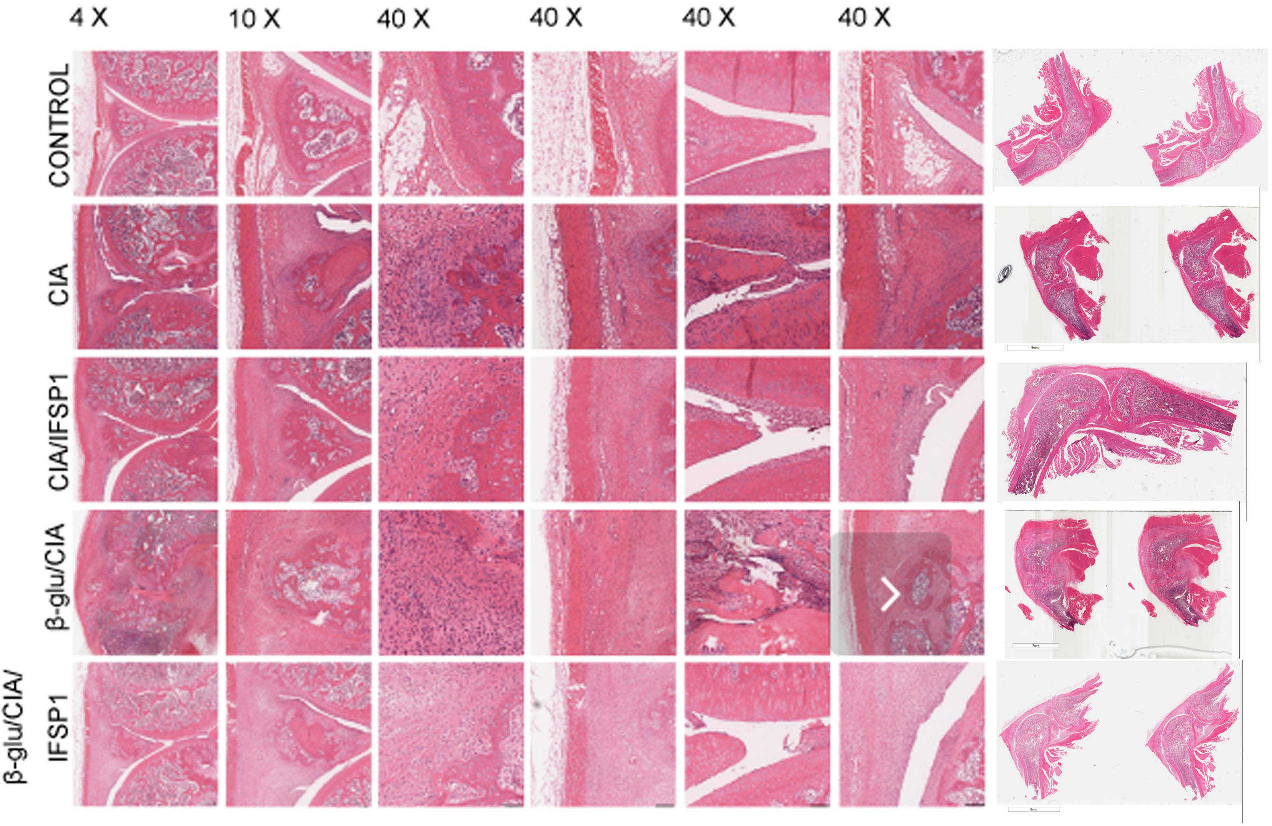

Raw data-H&E microscope images for Supplementary Figure 6B

S-Fig10-1A

Raw data-H&E microscope images for Supplementary Figure 10A

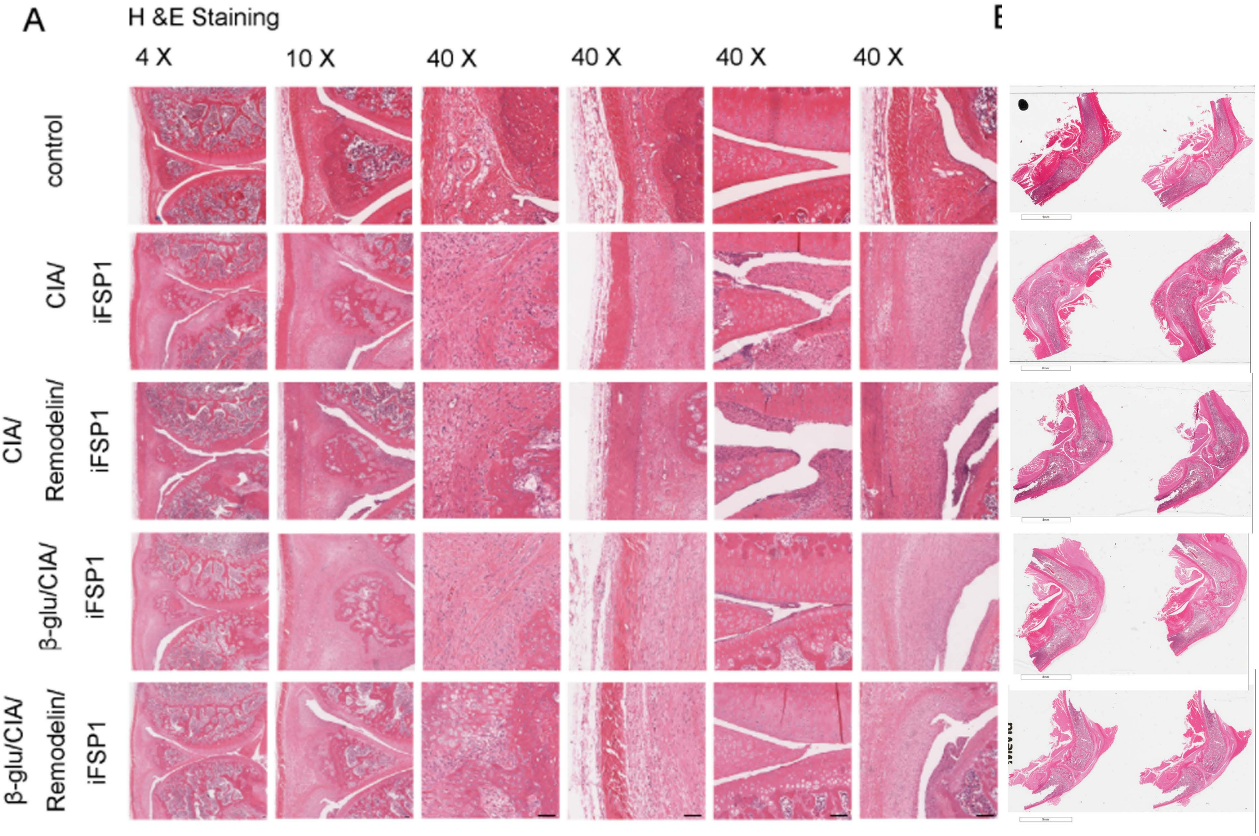

S-Fig13-1B

Raw data-H&E microscope images for Supplementary Figure 13B

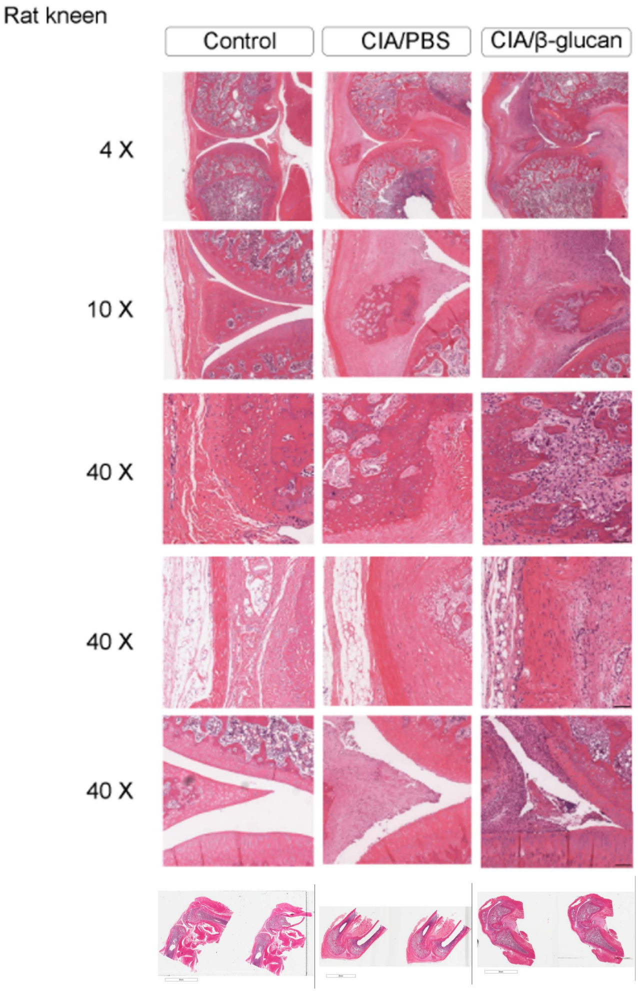

S-Fig13-1C

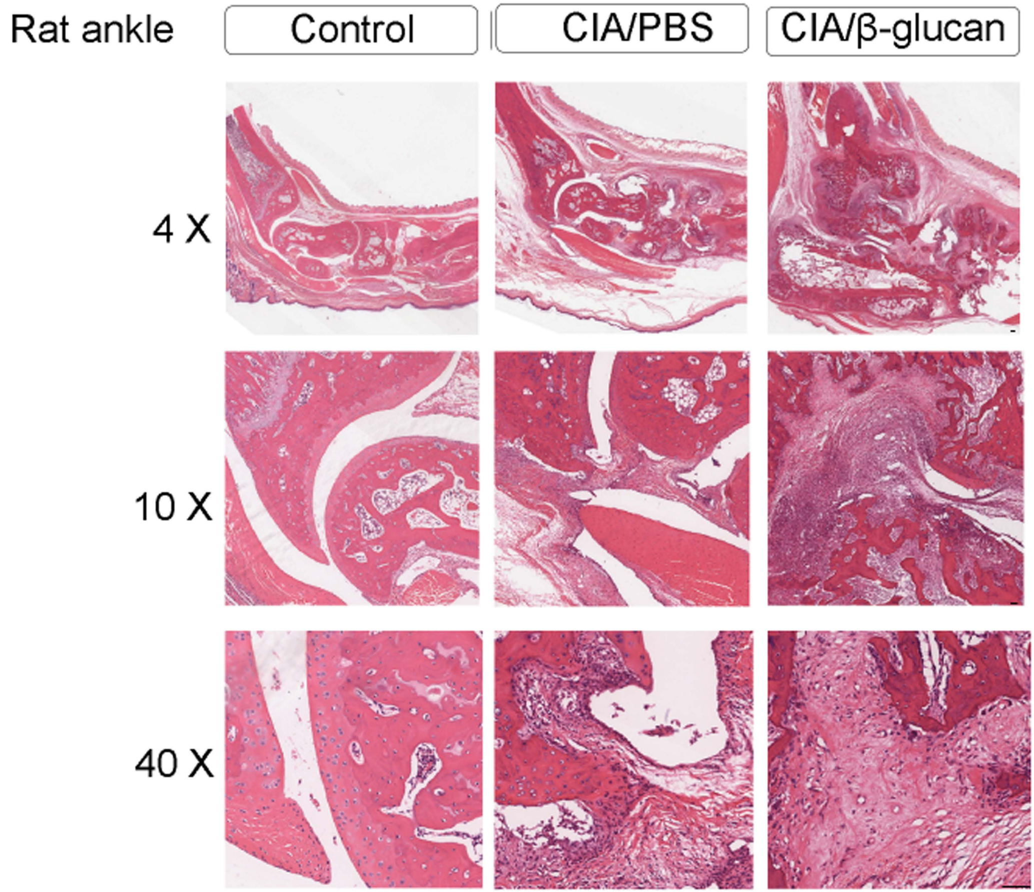

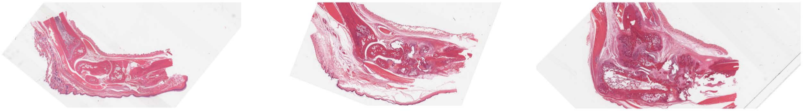

Raw data-H&E microscope images for Supplementary Figure 13C

S-Fig6-1B Safraing

Raw data-Safraing staining microscope images for Supplementary Figure 6B

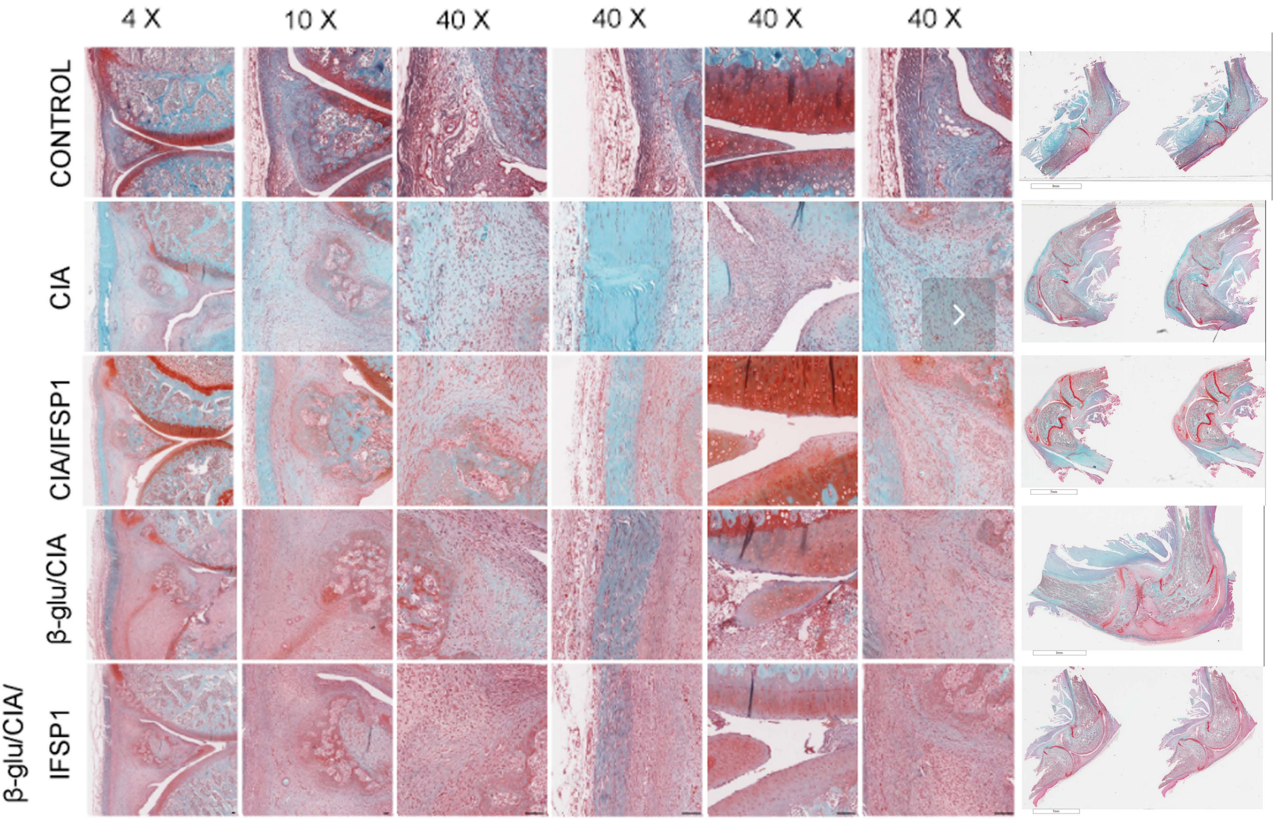

S-Fig10-1B

Raw data-Safraing staining microscope images for Supplementary Figure 10B

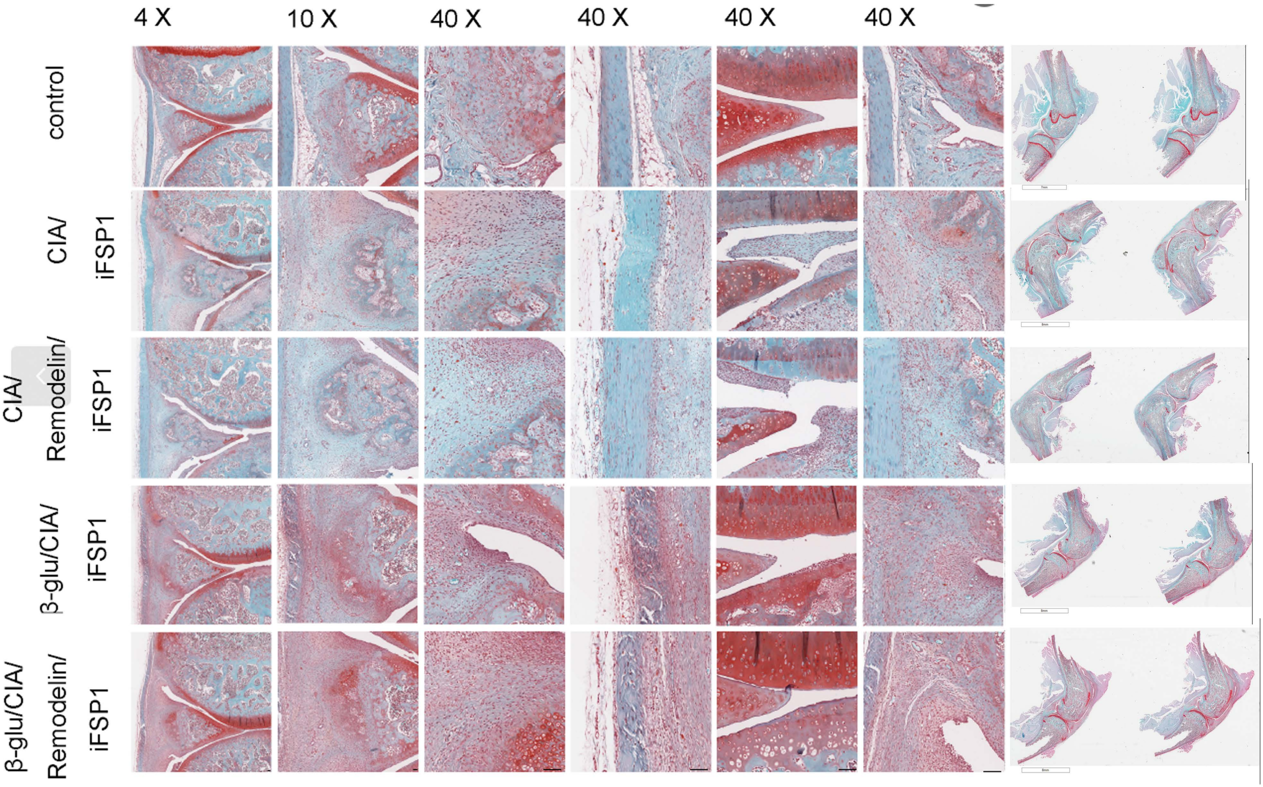

S-Fig6-1C

Raw data-Tuliudine Blue O staining microscope images for Supplementary Figure 6C

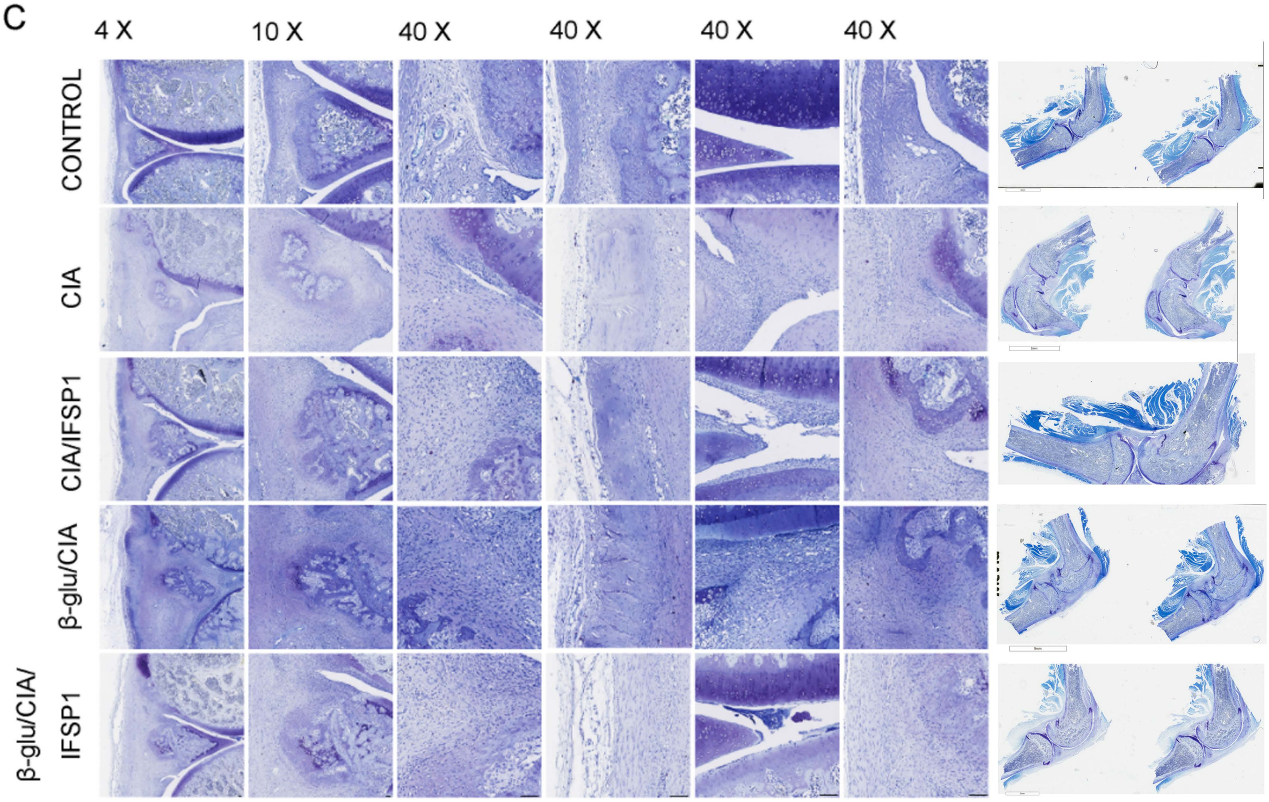

S-Fig10-1C

Raw data-Tuliudine Blue O staining microscope images for Supplementary Figure 10C

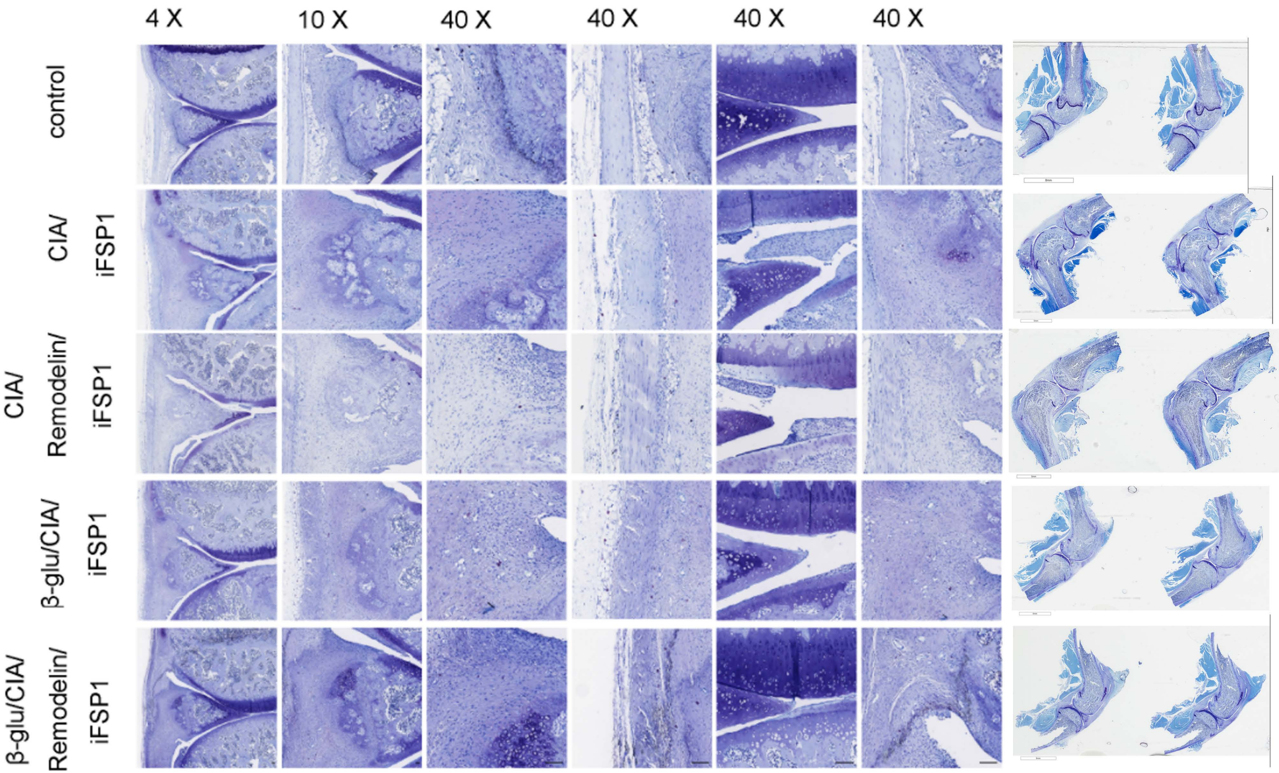

S-Fig5-1A

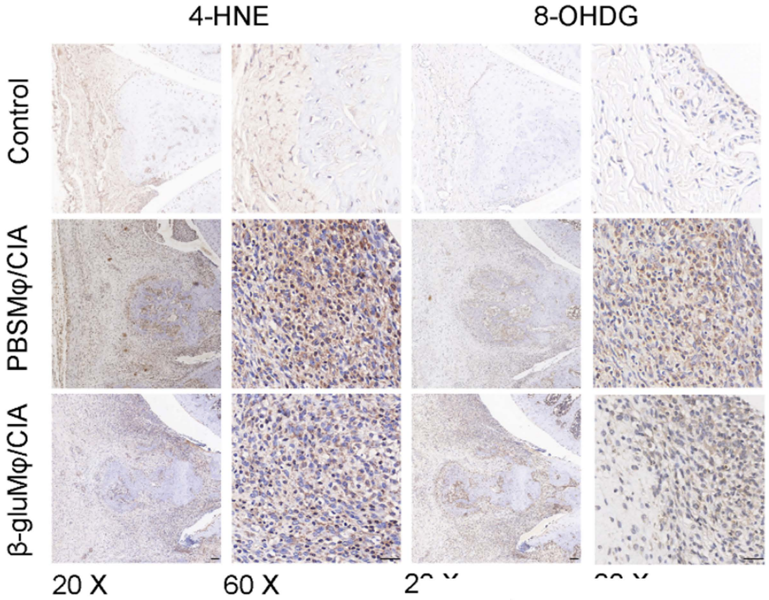

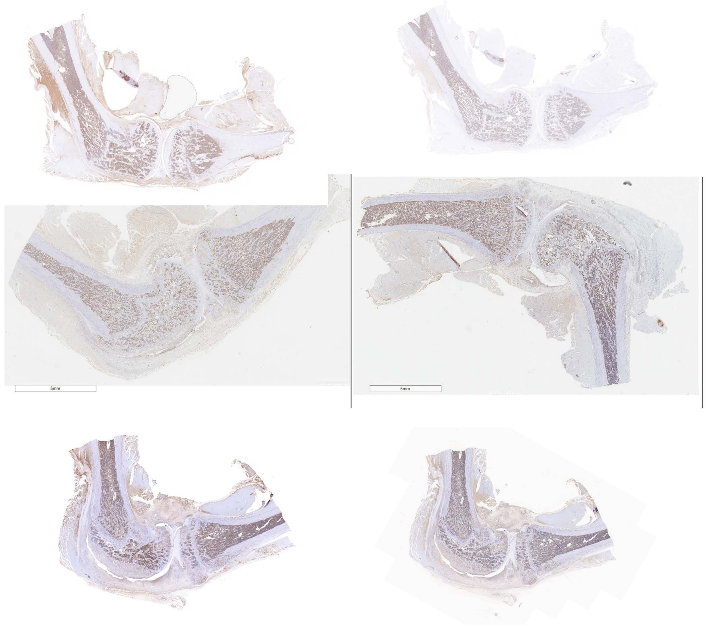

Raw data-Immunohistochemical microscope images for Supplementary Figure 5A

S-Fig9-1A

Raw data-Immunohistochemical microscope images for Supplementary Figure 9A

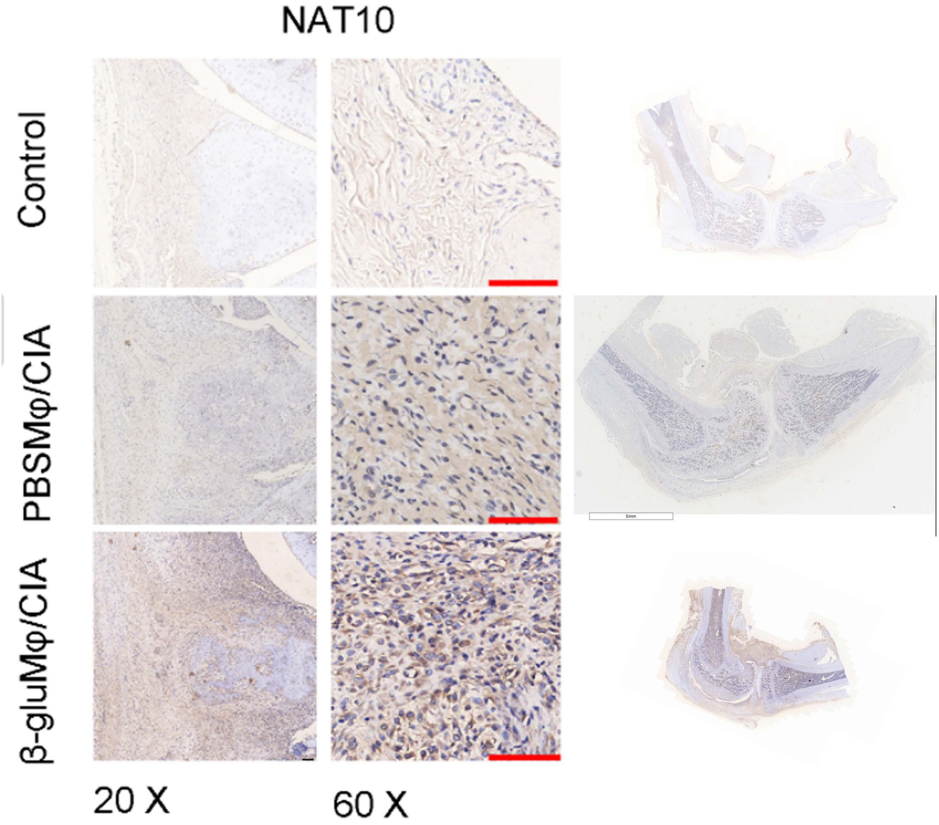

Fig1-H

Raw data- Immunofluorescent staining microscope images for Figure 1H

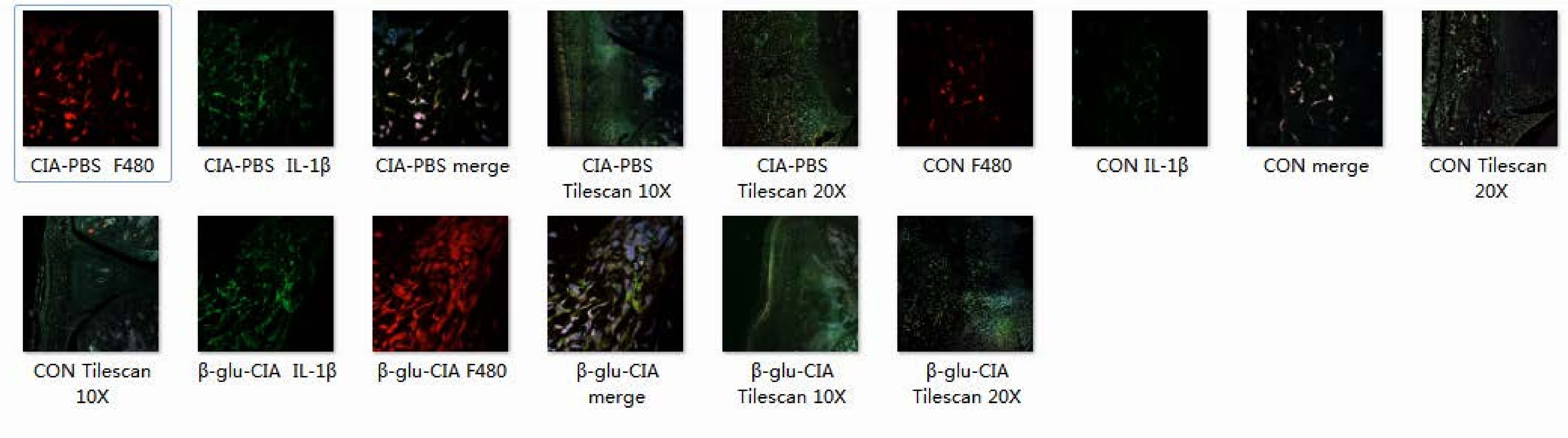

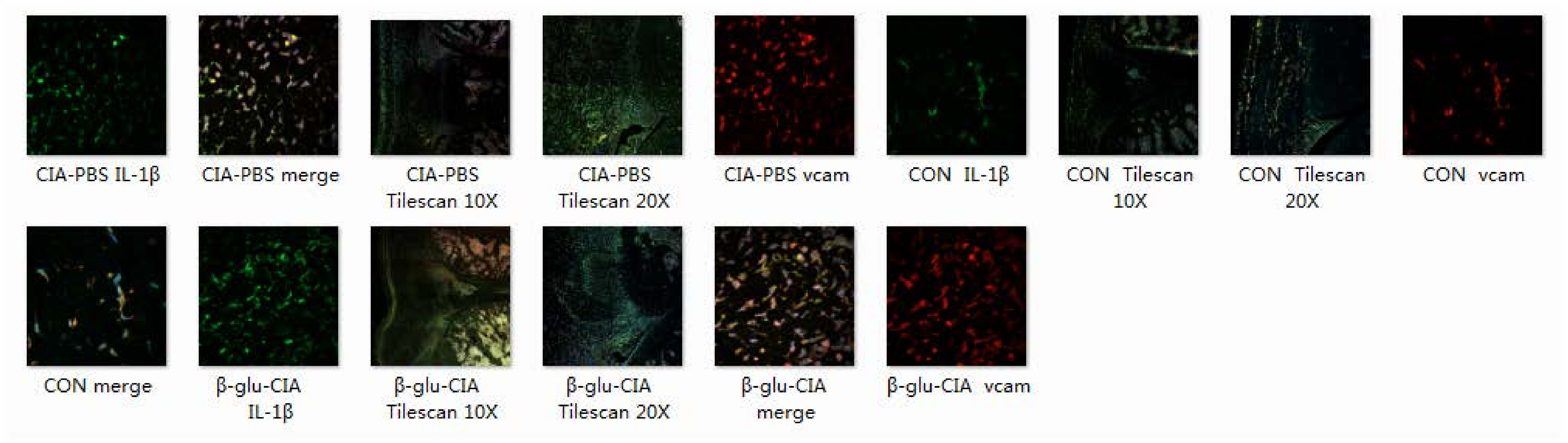

Fig-2G Raw data- Immunofluorescent staining microscope images for Figure 2G

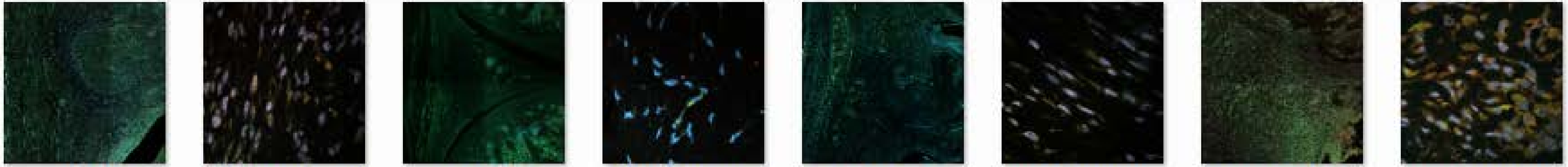

CIA 20X | CIA merge 60X | CON 20X | CON merge 60X | PBSMφ-CIA 20X | PBSMφ-CIA merge 60X | β-gluMφ-CIA 20X | β-gluMφ-CIA merge 60X

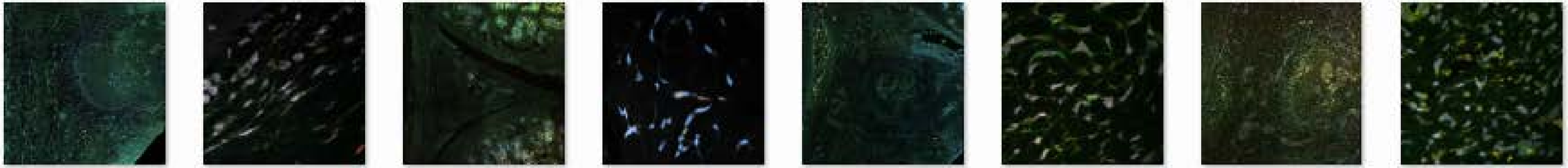

CIA 20X | CIA merge 60X | CON 20X | CON merge 60X | PBSMφ-CIA 20X | PBSMφ-CIA merge 60X | β-gluMφ-CIA 20X | β-gluMφ-CIA merge 60X

Fig3-F

Raw data- Immunofluorescent staining microscope images for Figure 3F

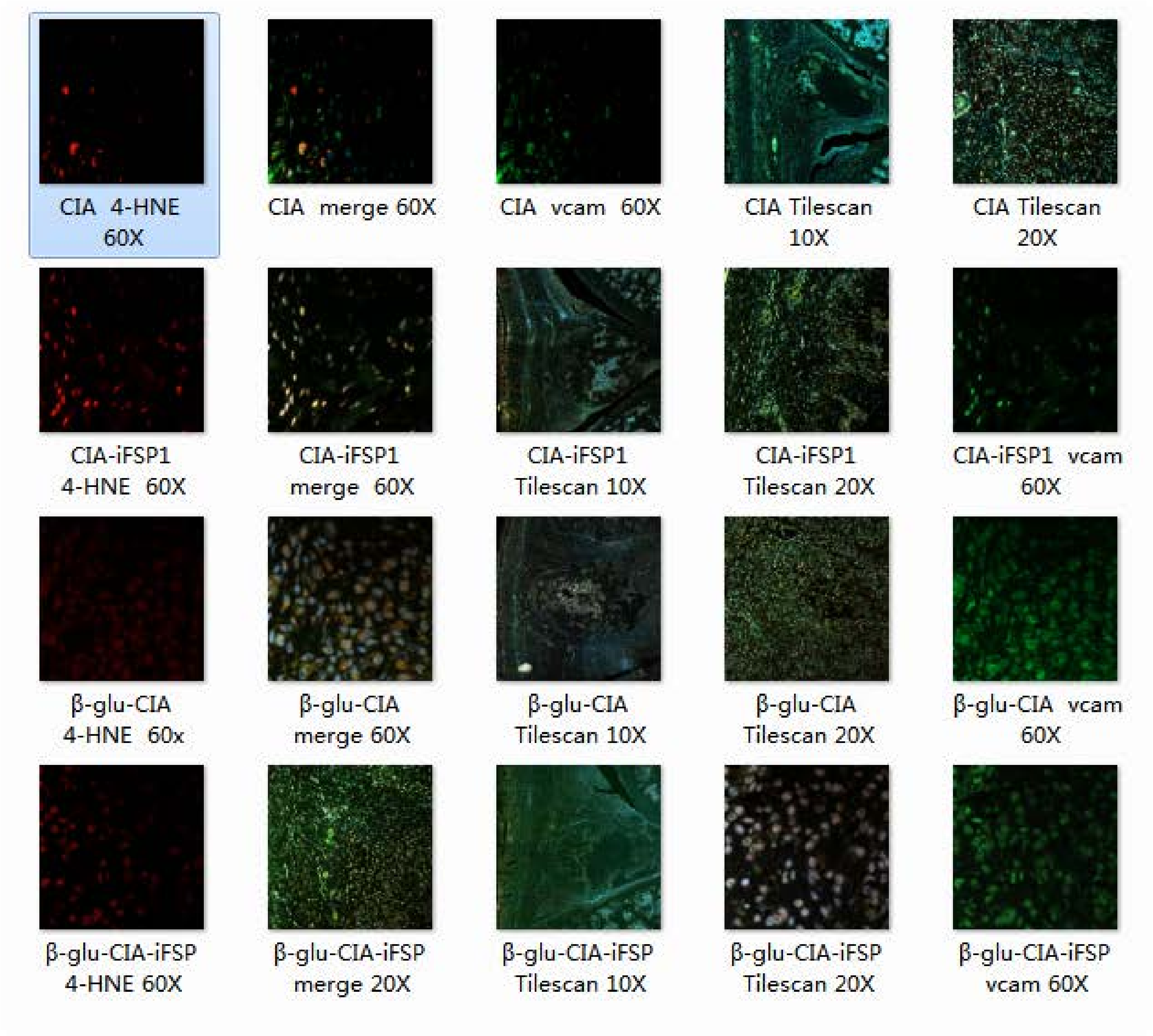

Fig-4D

Raw data- Immunofluorescent staining microscope images for Figure 4D

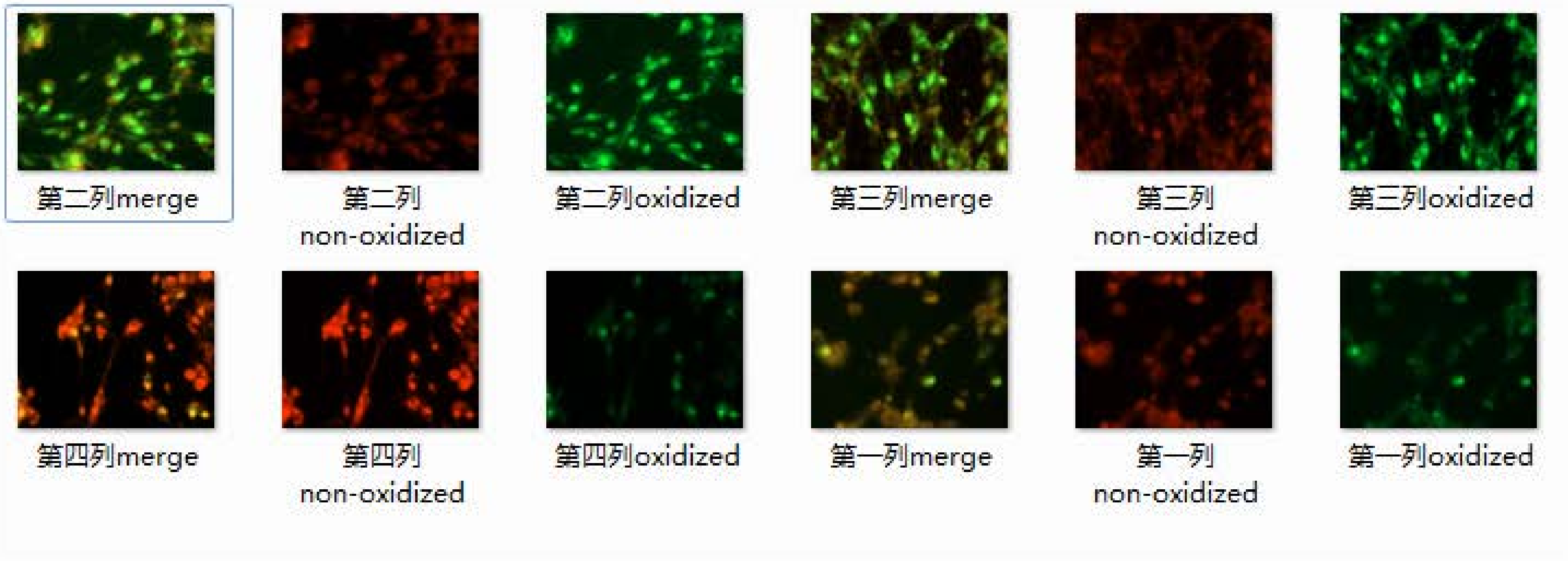

Fig-5G Raw data- Immunofluorescent staining microscope images for Figure 5G

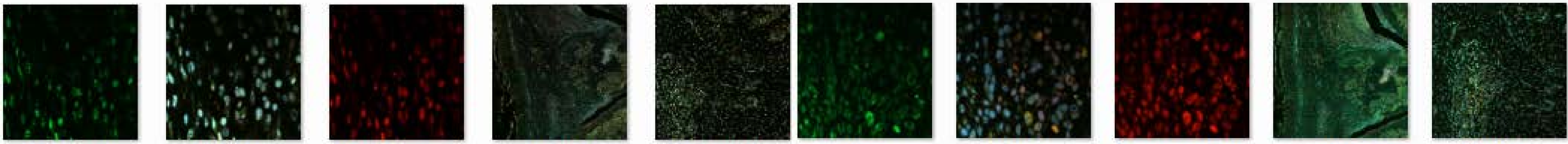

CIA-iFSP 4-HNE 60X
CIA-iFSP merge 60X
CIA-iFSP vcam 60X
CIA-iFSP Tilescan 10x
CIA-iFSP Tilescan 20x
CIA-iFSP 8-OHDG 60X
CIA-iFSP merge 60X
CIA-iFSP vcam 60X
CIA-iFSP Tilescan 10x
CIA-iFSP Tilescan 20x

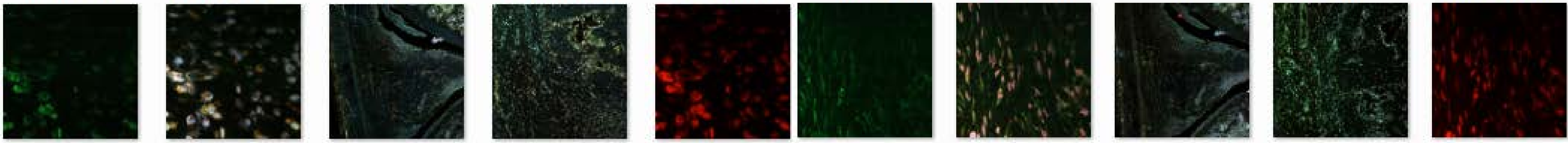

CIA-iFSP-Re 4-HNE 60X
CIA-iFSP-Re merge 60X
CIA-iFSP-Re Tilescan 10x
CIA-iFSP-Re Tilescan 20x
CIA-iFSP-Re vcam 60X
CIA-iFSP-Re 8-OHDG 60X
CIA-iFSP-Re merge 60X
CIA-iFSP-Re Tilescan 10x
CIA-iFSP-Re Tilescan 20x
CIA-iFSP-Re vcam 60X

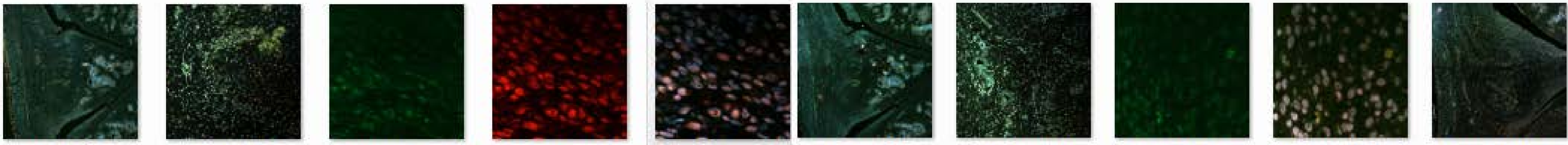

β-glu-CIA iFSP-Re Tilescan 10x
β-glu-CIA iFSP-Re Tilescan 20x
β-glu-CIA-iFSP 4-HNE 60X
β-glu-CIA-iFSP vcam 60X
β-glu-CIA-iFSP merge 60X
β-glu-CIA iFSP-Re Tilescan 10x
β-glu-CIA iFSP-Re Tilescan 20x
β-glu-CIA-iFSP 8-OHDG 60X
β-glu-CIA-iFSP merge 60X
β-glu-CIA-iFSP Tilescan 10x

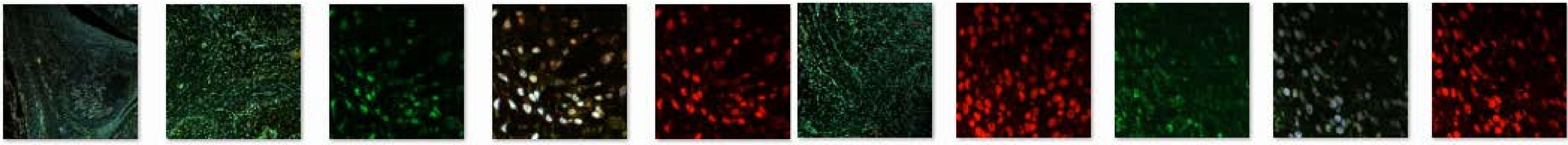

β-glu-CIA-iFSP Tilescan 10x
β-glu-CIA-iFSP Tilescan 20x
β -glu-CIA-iFSP-R e 4-HNE 60X
β -glu-CIA-iFSP-R e merge 60X
β -glu-CIA-iFSP-R e vcam 60X
β-glu-CIA-iFSP Tilescan 20x
β-glu-CIA-iFSP vacm 60X
β -glu-CIA-iFSP-R e 8-OHDG 60X
β -glu-CIA-iFSP-R e merge 60X
β -glu-CIA-iFSP-R e vcam 60X

Fig 6G

Raw data- Immunofluorescent staining microscope images for Figure 6G

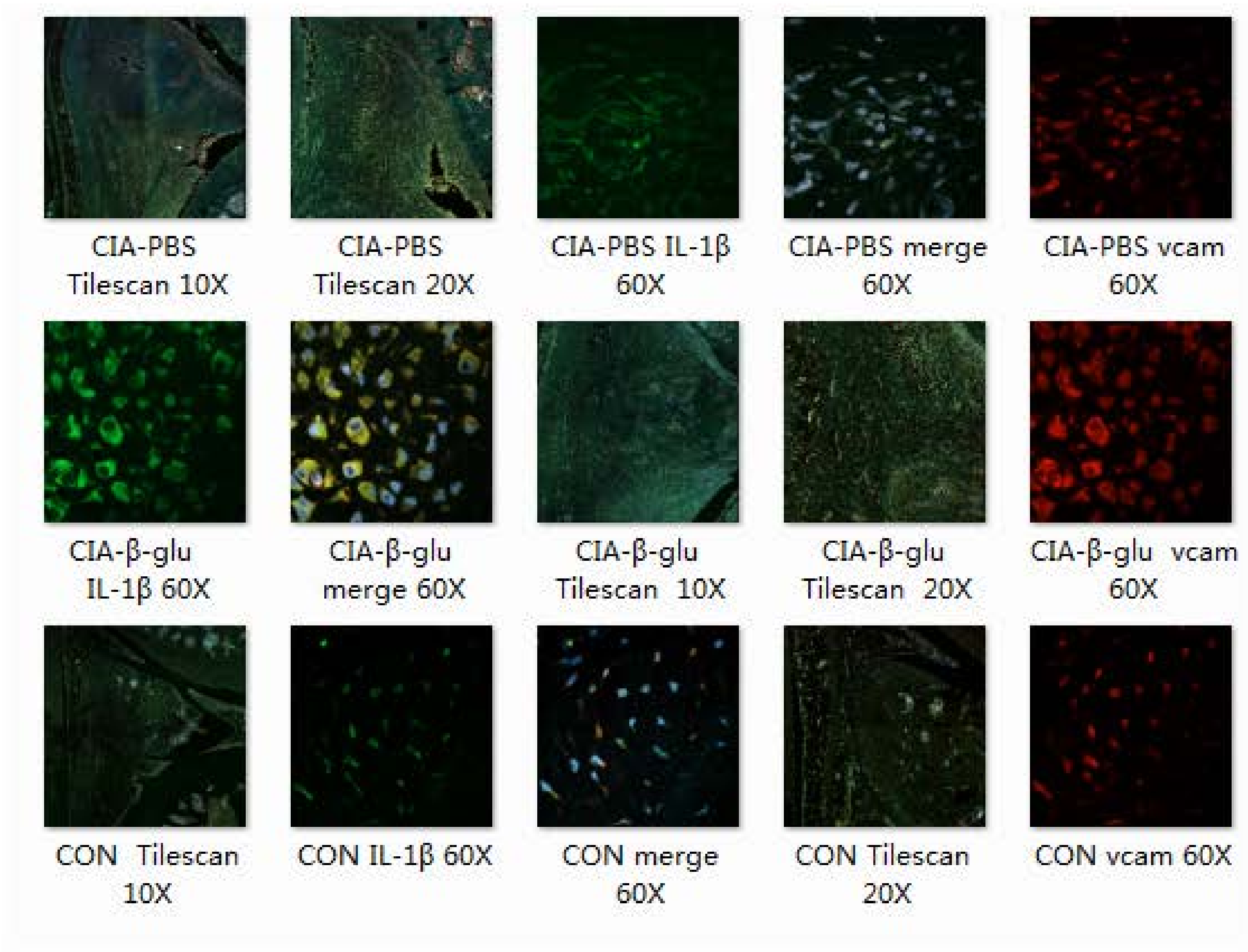

S-Fig3-1D Raw data- Immunofluorescent staining microscope images for Supplementary Figure 3D

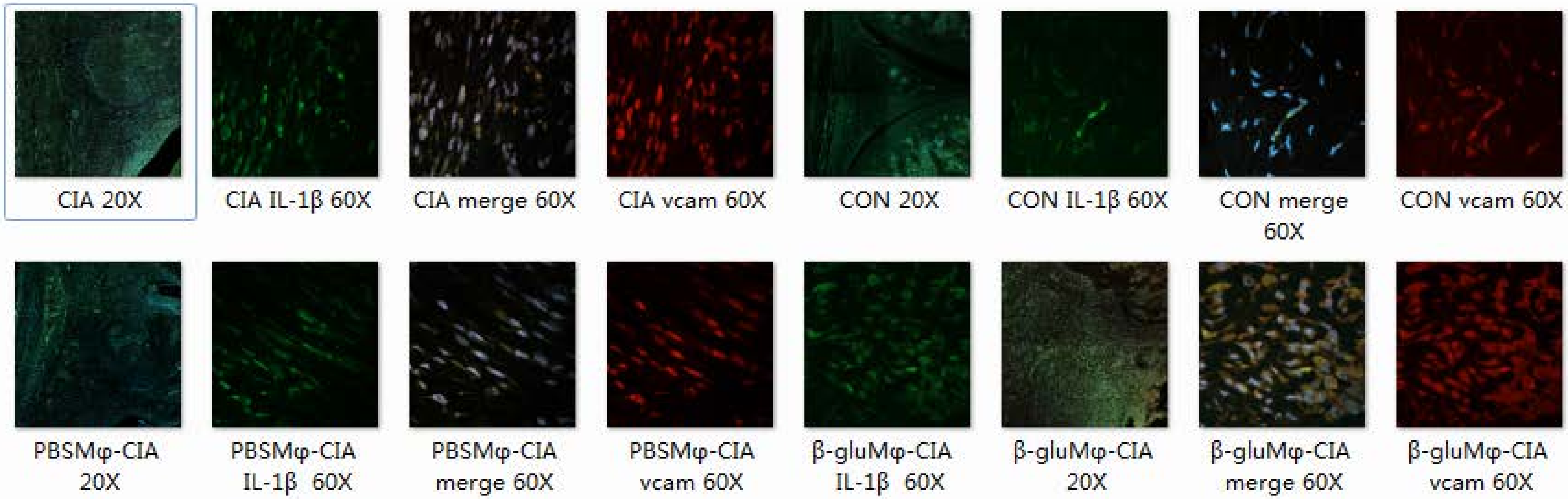

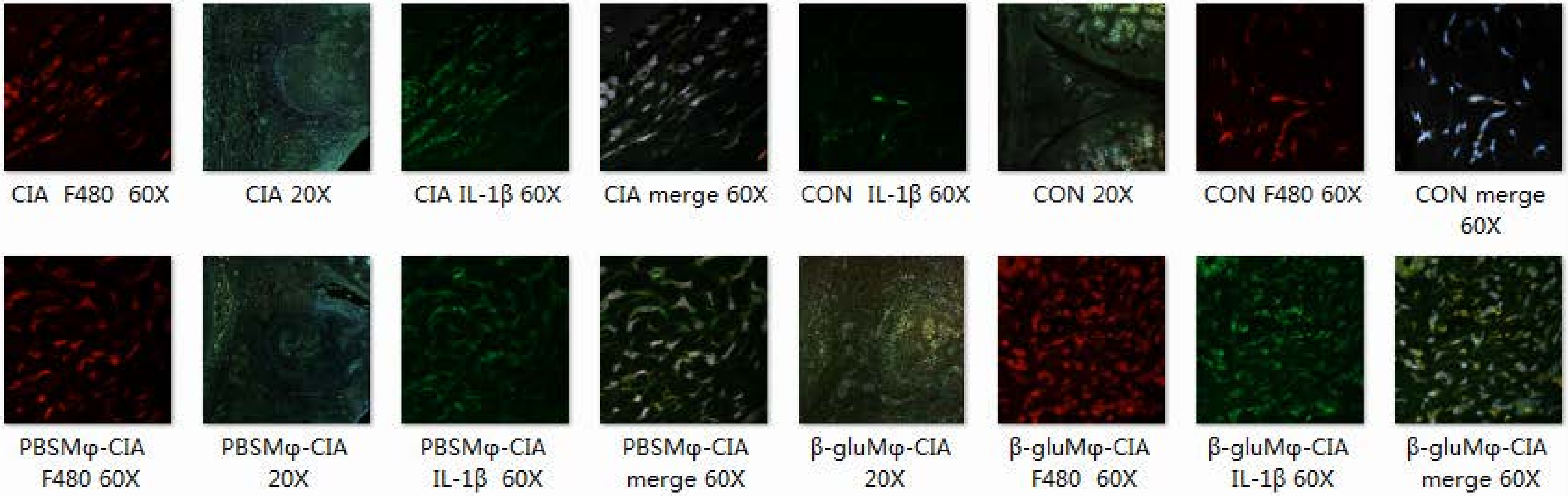

Raw data- Immunofluorescent staining microscope images for Supplementary Figure 5B

S-Fig5-1B

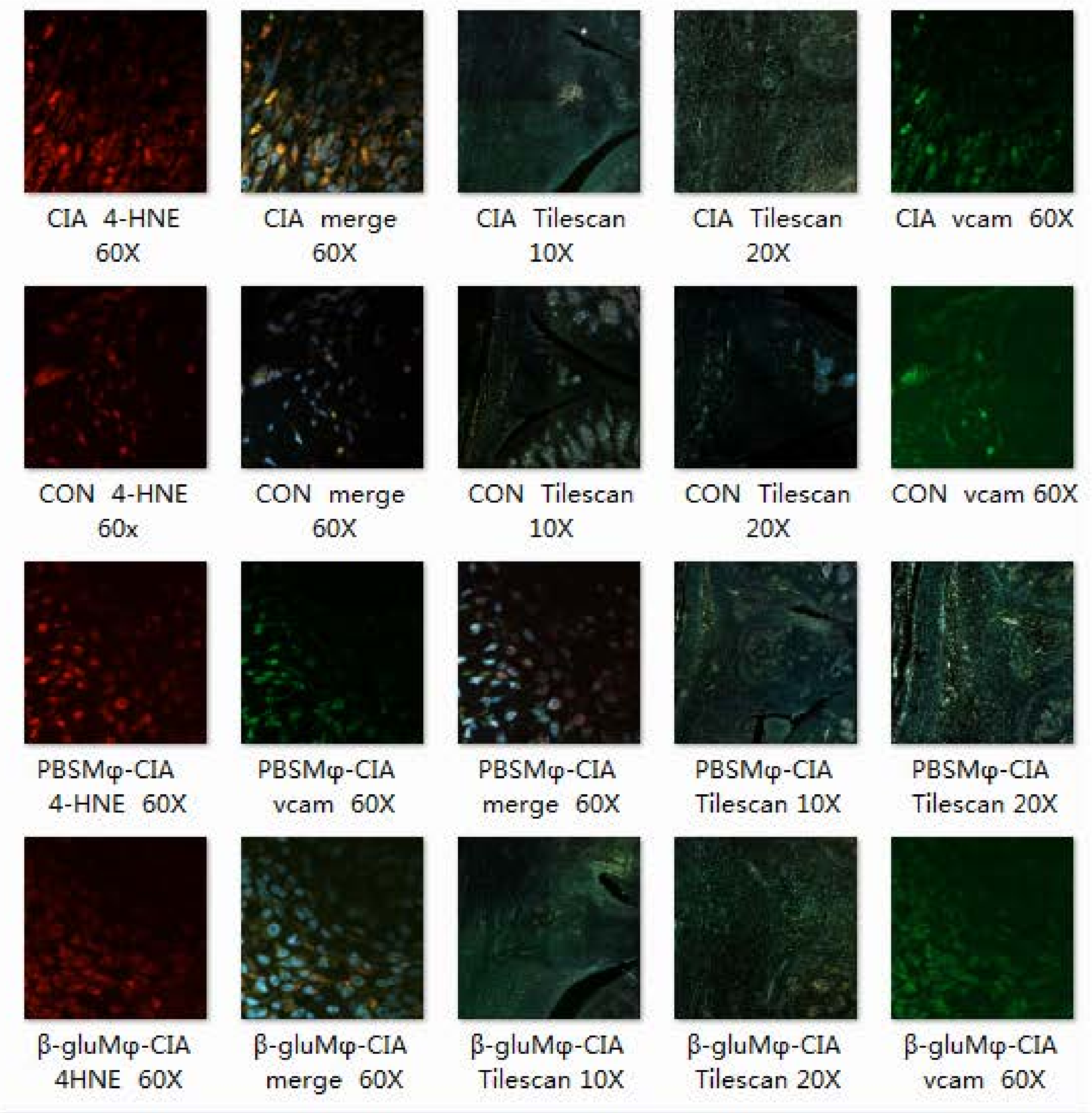

Raw data- Immunofluorescent staining microscope images for Supplementary Figure 5C

S-Fig5-1C

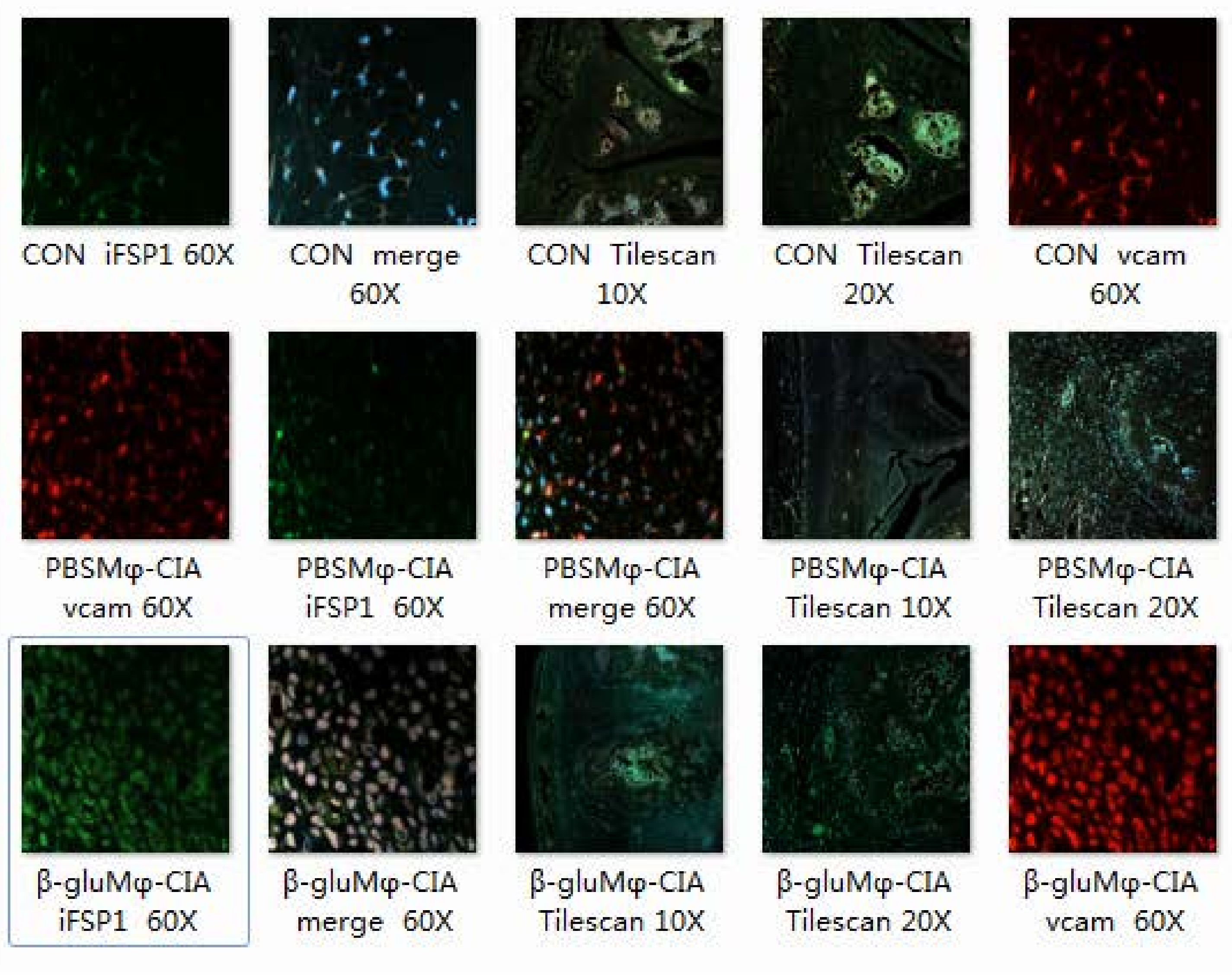

S-Fig9-1B

Raw data- Immunofluorescent staining microscope images for Supplementary Figure 9B

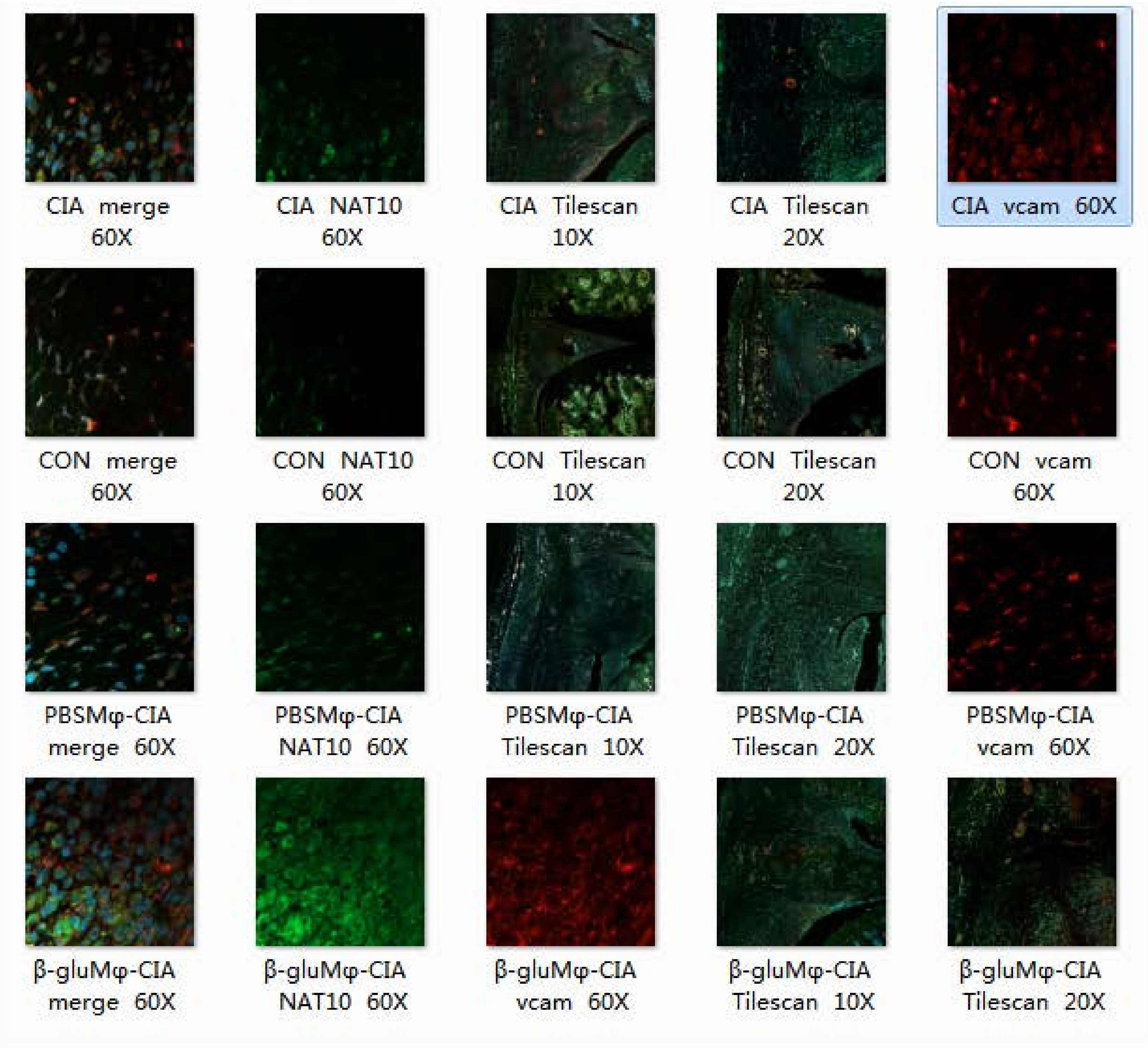

S-Fig11-1A

Raw data- Immunofluorescent staining microscope images for Supplementary Figure 11A

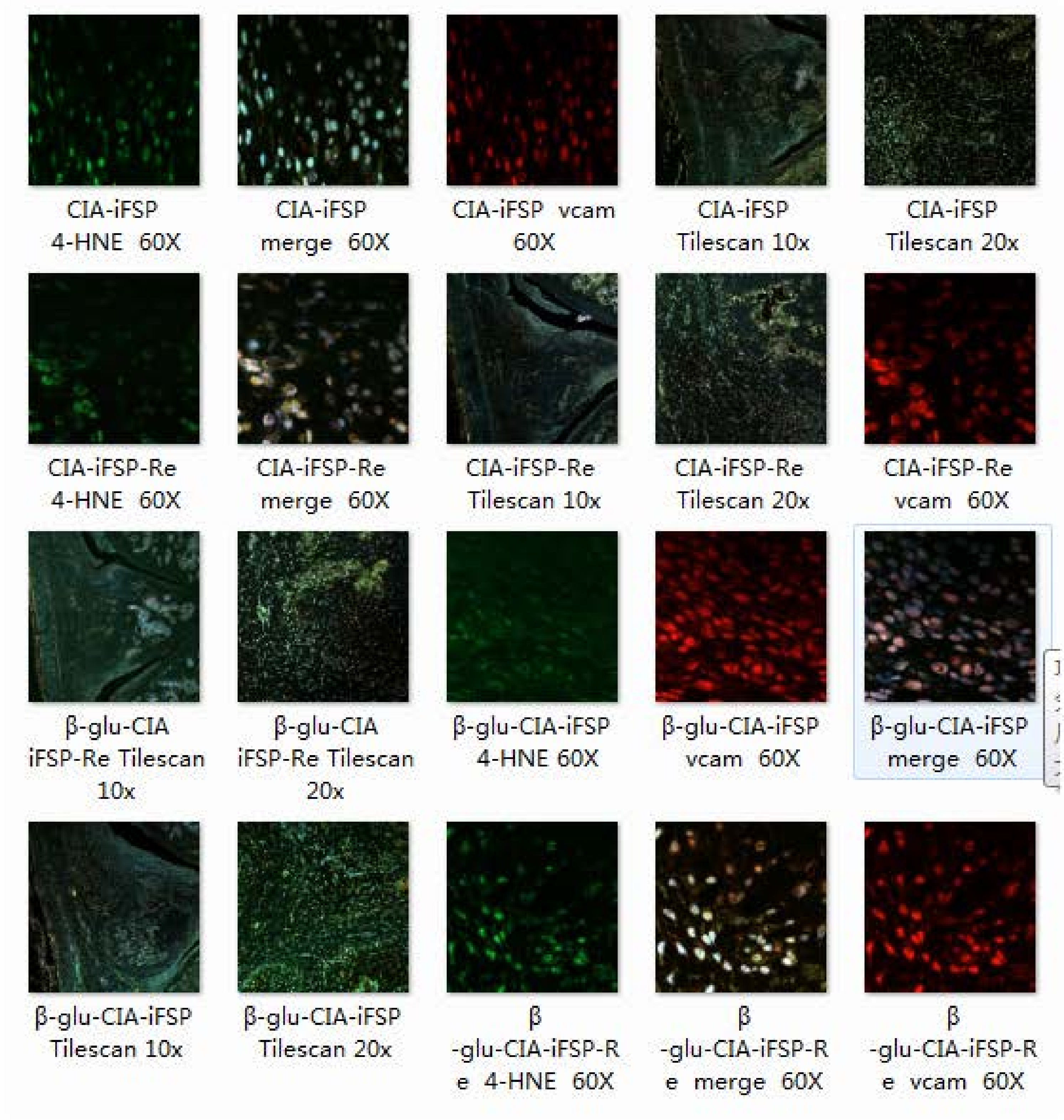

S-Fig11-1B

Raw data- Immunofluorescent staining microscope images for Supplementary Figure 11B

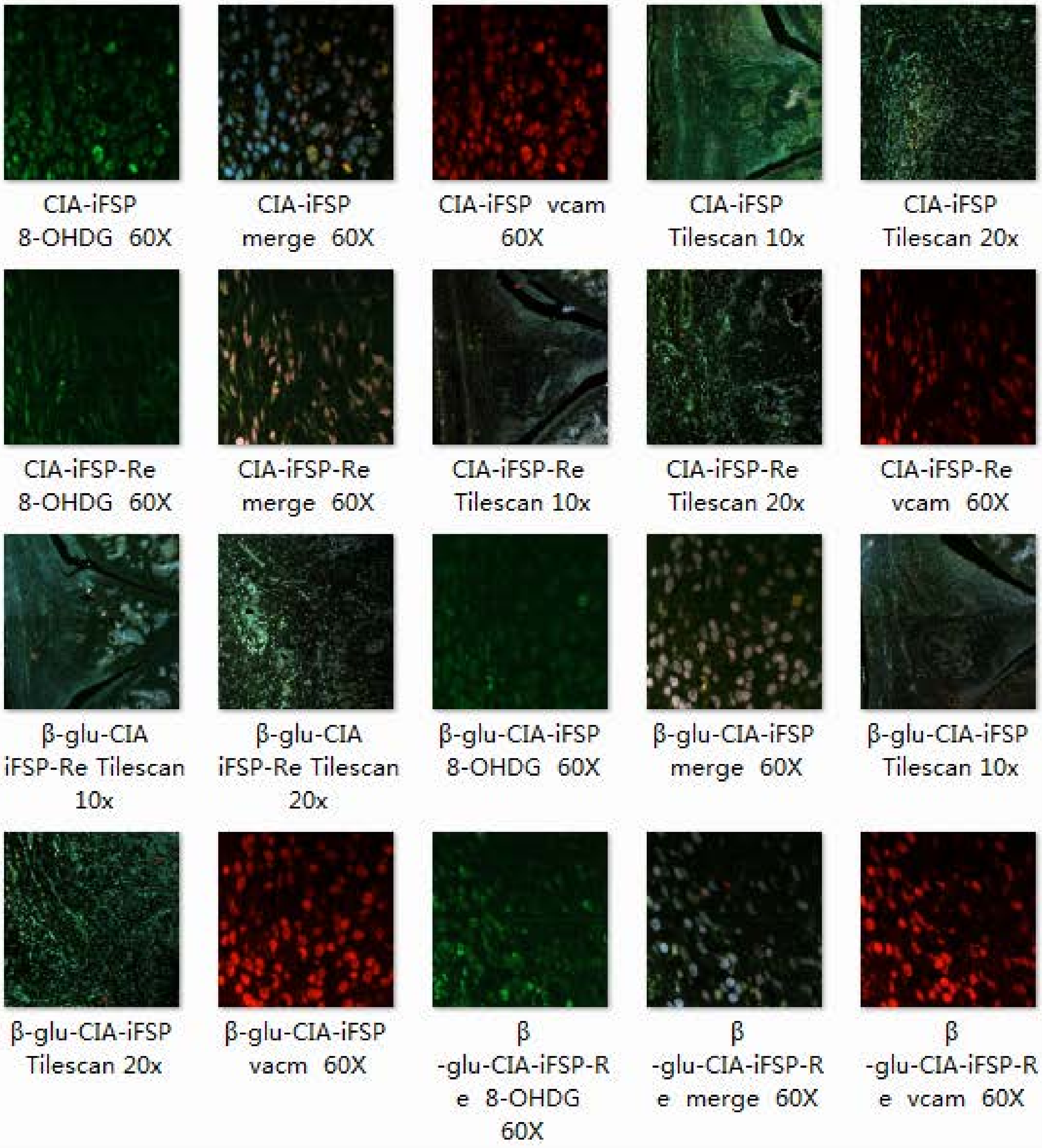

S-Fig13-1D

Raw data- Immunofluorescent staining microscope images for Supplementary Figure 13D

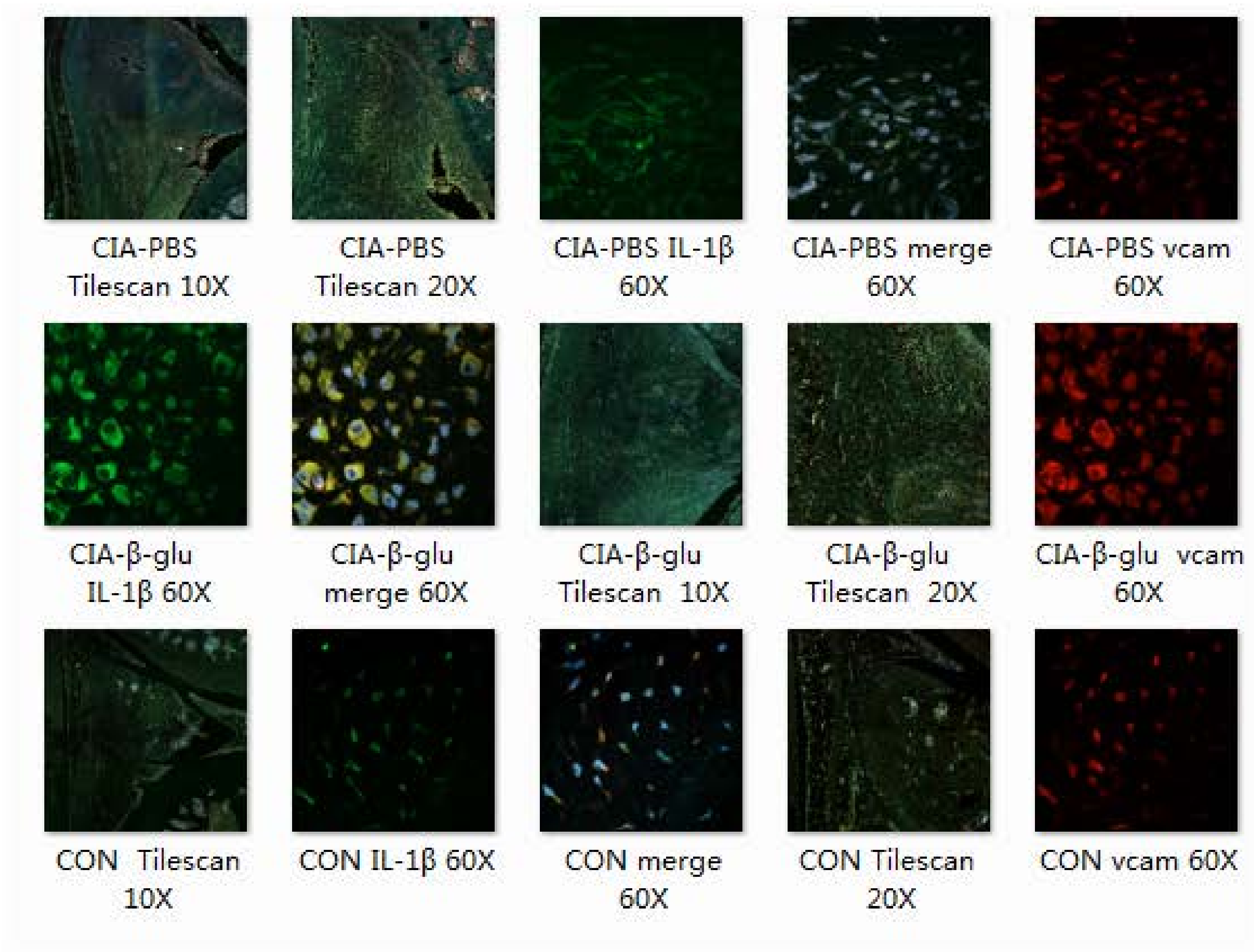

S-Fig13-1E

Raw data- Immunofluorescent staining microscope images for Supplementary Figure 13E

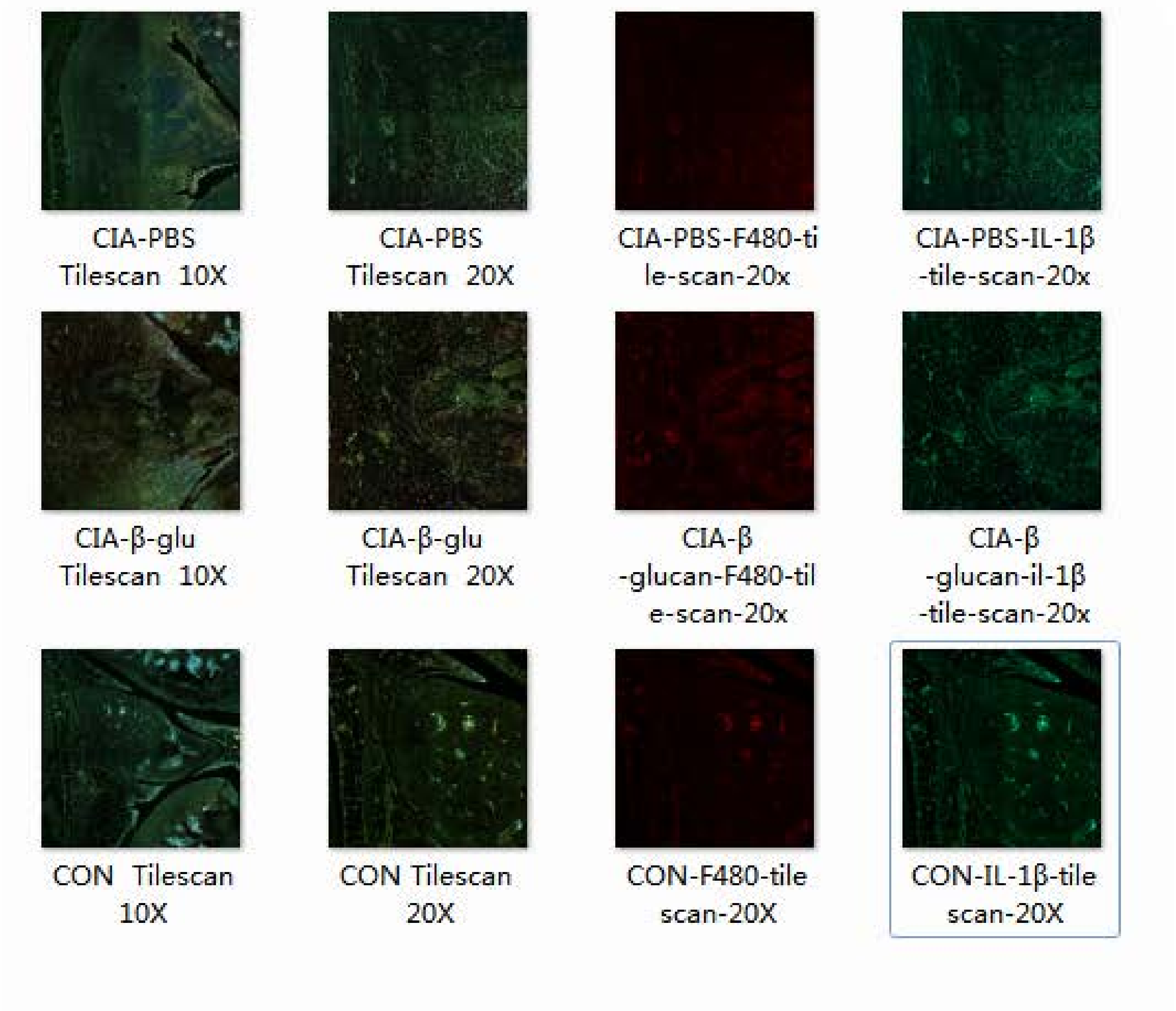

Supplement: Supplementary file 1 — Supporting Information [file ADVS-12-e04245-s001.pdf]
